# Imiquimod has strain-dependent effects in mice and does not uniquely model human psoriasis

**DOI:** 10.1186/s13073-017-0415-3

**Published:** 2017-03-09

**Authors:** William R. Swindell, Kellie A. Michaels, Andrew J. Sutter, Doina Diaconu, Yi Fritz, Xianying Xing, Mrinal K. Sarkar, Yun Liang, Alex Tsoi, Johann E. Gudjonsson, Nicole L. Ward

**Affiliations:** 1 Ohio University, Heritage College of Osteopathic Medicine, Athens, OH 45701-2979 USA; 2 Department of Dermatology, University of Michigan, Ann Arbor, MI 48109-2200 USA; 3 Department of Dermatology, Case Western Reserve University, 10900 Euclid Ave, Cleveland, OH 44106 USA; 4 The Murdough Family Center for Psoriasis, Case Western Reserve University, Cleveland, OH USA

**Keywords:** Aldara, Differentiation, Epidermis, Interferon, IL-17A, Keratinocyte, RNA-seq, Toll-like receptor

## Abstract

**Background:**

Imiquimod (IMQ) produces a cutaneous phenotype in mice frequently studied as an acute model of human psoriasis. Whether this phenotype depends on strain or sex has never been systematically investigated on a large scale. Such effects, however, could lead to conflicts among studies, while further impacting study outcomes and efforts to translate research findings.

**Methods:**

RNA-seq was used to evaluate the psoriasiform phenotype elicited by 6 days of Aldara (5% IMQ) treatment in both sexes of seven mouse strains (C57BL/6 J (B6), BALB/cJ, CD1, DBA/1 J, FVB/NJ, 129X1/SvJ, and MOLF/EiJ).

**Results:**

In most strains, IMQ altered gene expression in a manner consistent with human psoriasis, partly due to innate immune activation and decreased homeostatic gene expression. The response of MOLF males was aberrant, however, with decreased expression of differentiation-associated genes (elevated in other strains). Key aspects of the IMQ response differed between the two most commonly studied strains (BALB/c and B6). Compared with BALB/c, the B6 phenotype showed increased expression of genes associated with DNA replication, IL-17A stimulation, and activated CD8+ T cells, but decreased expression of genes associated with interferon signaling and CD4+ T cells. Although IMQ-induced expression shifts mirrored psoriasis, responses in BALB/c, 129/SvJ, DBA, and MOLF mice were more consistent with other human skin conditions (e.g., wounds or infections). IMQ responses in B6 mice were most consistent with human psoriasis and best replicated expression patterns specific to psoriasis lesions.

**Conclusions:**

These findings demonstrate strain-dependent aspects of IMQ dermatitis in mice. We have shown that IMQ does not uniquely model psoriasis but in fact triggers a core set of pathways active in diverse skin diseases. Nonetheless, our findings suggest that B6 mice provide a better background than other strains for modeling psoriasis disease mechanisms.

**Electronic supplementary material:**

The online version of this article (doi:10.1186/s13073-017-0415-3) contains supplementary material, which is available to authorized users.

## Background

The study of psoriasis has been hindered by the lack of a mouse phenotype that fully recapitulates the complex features of lesions in psoriasis patients [1, 2]. In recent years, topical application of imiquimod (IMQ) cream (Aldara™) to murine skin has increasingly been used as an acute psoriasiform murine model, in part due to its convenience and ability to elicit a dermatitis resembling some aspects of psoriasis [3, 4]. IMQ is a Toll-like receptor (TLR7/8) agonist that can be applied to mouse skin to elicit erythema, scaling, keratinocyte (KC) proliferation with acanthosis, altered KC differentiation (parakeratosis), and a dermal infiltrate that includes T cells [3]. The phenotype additionally involves induction of IL-17/IL-23 axis cytokines (e.g., IL-17A, IL-17 F, IL-22 and IL-23) and is partially dependent upon IL-23, IL-17 family cytokines, and the presence of T lymphocytes [3, 5, 6]. All of these features are consistent with human psoriasis lesions and accepted models of cutaneous lesion development [1, 2]. IMQ has thus been viewed as a convenient and easy-to-use mouse model of acute inflammatory response, which has been applied to facilitate mechanistic studies that would otherwise prove difficult or impossible to perform in humans.

Psoriasis is a complex genetic disease that depends upon interactions among environmental influences and disease variants at multiple loci [7]. Given this genetic basis, it is reasonable to expect that any realistic mouse model of psoriasis would be sensitive to genetic background, with certain aspects of the psoriasiform phenotype varying among mouse strains. Most studies of IMQ-induced lesions in mouse are performed using one strain (often B6 or BALB/c) and commonly only one sex has been investigated (frequently females) [4, 8]. It is not unusual for either the strain or sex of experimental animals to go unreported in published research [8], and the question of whether mechanistic findings are sensitive to genetic background or sex is seldom addressed. Those few studies that have analyzed multiple mouse strains, however, suggest that IMQ responses can be strain-specific [4, 9]. For example, BALB/c lesions develop more rapidly than B6 lesions following IMQ application [3, 4]. B6 mice may also have less severe systemic responses, which include dehydration leading to weight loss and decreased activity levels [3]. Even the more closely related C57BL/6 J and C57BL/6 N substrains were recently shown to exhibit differential IMQ responses, with C57BL/6 J mice exhibiting increased erythema, desquamation, epidermal proliferation, and skin inflammation [9]. It has been suggested that differences in IMQ response among these genotypes (C57BL/6 J, C57BL/6 N, and BALB/c) may be related to cutaneous IL-22 activation [3, 9], although other possibilities have not been investigated systematically.

The practical significance of strain-specific IMQ responses is that studies utilizing different strains may reach conflicting conclusions regarding the cell types, mediators, and pathways required for the psoriasiform phenotype [10]. This has been observed for other inflammatory skin phenotypes in mice [11] and can diminish the apparent repeatability of experimental findings, potentially leading to misdirected research effort and mechanistic conclusions that cannot be generalized [12, 13]. Examples illustrating genetic background as a possible pitfall in mechanistic studies are abundant and can be drawn from numerous contexts and model organisms [14–17]. Addressing these issues requires systematic investigation of IMQ responses in multiple mouse strains to better understand the degree to which the psoriasiform phenotype is sex- and strain-dependent. At the molecular level, this can lead to identification of pathways that respond similarly to IMQ regardless of genotype, which may in turn represent the most reliable center points for mechanistic investigations. At the same time, those responses that are strain-dependent may point towards allelic variants that shape innate immune responses, suggesting new avenues for studies of gene function [18]. Ultimately, such investigation should help refine the IMQ model by clarifying its strengths and limitations relative to human psoriasis, which should improve the chance that new findings are successfully translated to clinical studies with patients [12].

This study used RNA-seq to investigate the psoriasiform phenotype that develops following topical Aldara (5% IMQ) treatment in male and female mice of seven laboratory mouse strains (C57BL/6 J (B6), BALB/cJ, CD1, DBA/1 J, FVB/NJ, 129X1/SvJ, and MOLF/EiJ) [19]. The IMQ psoriasiform phenotype has previously been evaluated using microarrays [2, 20–22], but to our knowledge has not yet been evaluated by RNA-seq. Previously, we used microarrays to compare cutaneous phenotypes of five psoriasis mouse models and to score the similarities and differences of such models relative to human psoriatic plaques [2]. Following the same principle, the current analysis uses RNA-seq to evaluate the IMQ psoriasiform phenotype that manifests in males and females from seven strains. These strains include one representative from each major group identified from genetic analysis of existing mouse diversity [23]. Six of seven strains are inbred, but CD1 is an outbred strain maintained with minimal inbreeding to retain heterozygosity and genetic diversity [24]. Our goals were to evaluate the degree to which transcriptome-level IMQ responses depend on strain and sex and to distinguish shared responses from those that are strain- or sex-specific. We additionally identify genes, pathways, and cell types similarly altered in mouse phenotypes and human psoriasis, as well as discordant patterns representing aspects of human psoriasis not recapitulated by IMQ treatment.

## Methods

### Laboratory mice

Male (*n* = 5/strain) and female (*n* = 5/strain) mice were purchased at 8 weeks of age and allowed 14 days to acclimate prior to starting experiments. Most strains were purchased from the Jackson Laboratory (C57BL/6 J (B6), catalog number 000664; BALB/cJ, catalog number 000651; DBA/1 J, catalog number 000670; FVB/NJ, catalog number 001800; 129X1/SvJ, catalog number 000691; MOLF/EiJ, catalog number 000550). CD1 (ICR) mice were purchased from Charles River laboratories (catalog number 022). Mice were maintained within the Case Western Reserve University animal vivarium under specific-pathogen-free conditions and provided standard rodent chow diet (4.5% fat by weight, 14% kcal, Lab Diet P3000).

### Imiquimod protocol

A topical dose of 62.5 mg IMQ cream (5% Aldara; 3 M Pharmaceuticals) was applied daily to the shaved back region of mice. Control (CTL) animals were treated similarly with a non-toxic lanolin-derived occlusion cream (VWR, catalog number 56614-414). Mice in the CTL group did not exhibit macroscopic indications of skin irritation or inflammation (e.g., erythema, scaling, or induration). IMQ and CTL treatments were carried out for 5 consecutive days and mice were sacrificed on day 6 following injection with euthanasia solution (ketamine, 16.5 mg/mL; xylazine, 1.65 mg/mL) followed by cervical dislocation. Mice were weighed daily and provided saline (i.p.) as needed to supplement fluid loss associated with IMQ treatment, a well-known side effect [25]. This limited weight loss in most strains, although we note that one of five MOLF males and one of five MOLF females died prior to day 6. Samples from these mice were excluded from analyses, but for all other mice back skin and spleens were collected immediately upon sacrifice on day 6, flash frozen, and stored at −80 °C prior to isolation of total RNA. Skin adjacent to that collected for RNA was placed into formalin and processed for histology. For all strains, tissues were collected at approximately the same time of day to limit gene expression variation associated with circadian rhythms [26].

### Body weight, epidermal thickness, and spleen weight

**Fig. 1:**
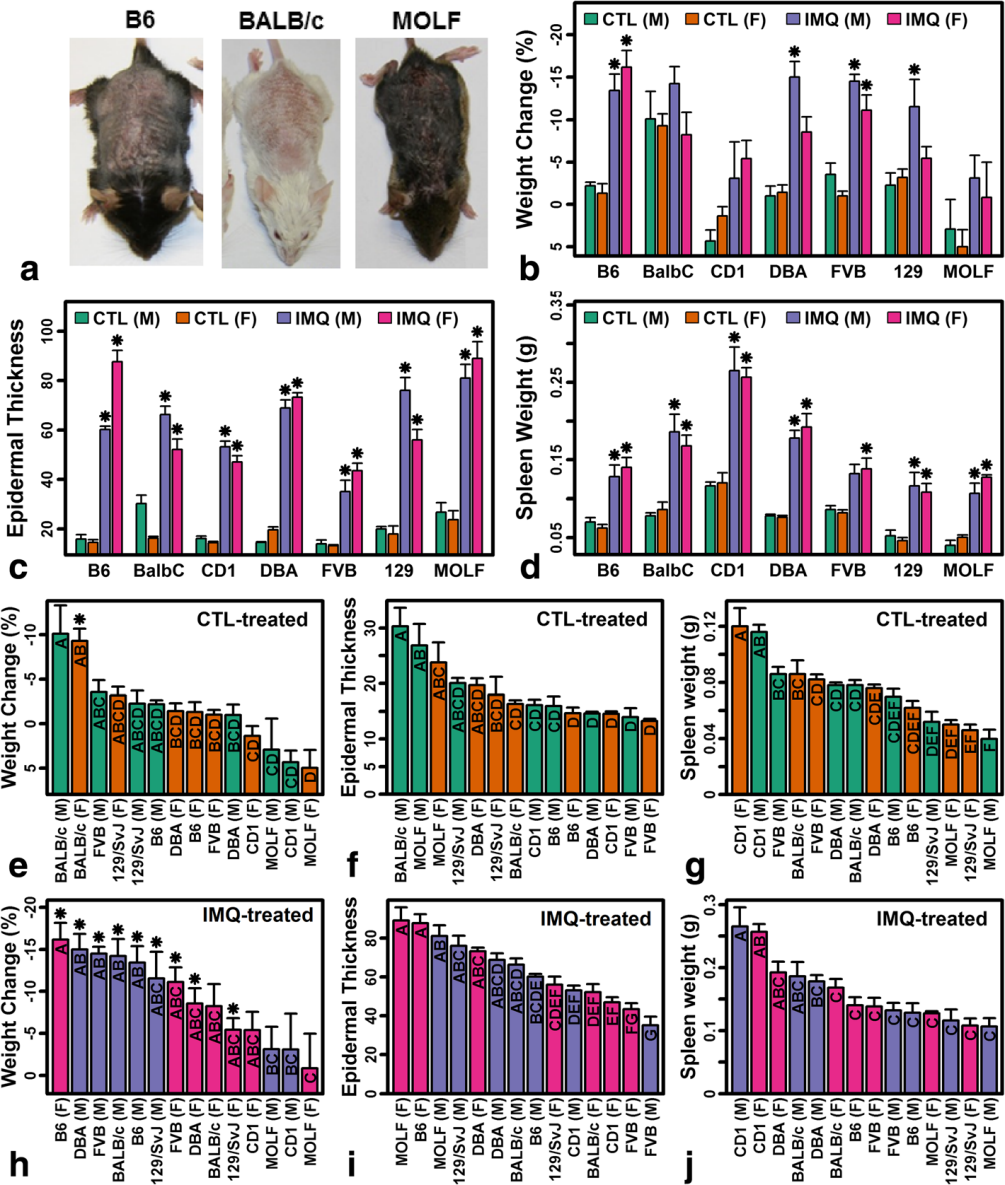
Cutaneous and systemic IMQ responses. **a** IMQ dermatitis in B6, BALB/c, and MOLF mice. **b** Weight loss following IMQ treatment (*P < 0.05, IMQ versus sex-matched control). **c** Epidermal thickness (*P < 0.05, IMQ versus sex-matched control; measured using microscopy). **d** Spleen weight (*P < 0.05, IMQ versus sex-matched control). **e**–**g** CTL-treated samples. **h**–**j** IMQ-treated samples. In **e**–**j**, groups without the same letter differ significantly (*P* < 0.05, Tukey honest significant difference). In **b**–**j**, three to five mice were evaluated for each strain, sex, and treatment (*error bars* represent standard error of the mean). *F* female, *M* male

Body weight was measured in all mice before onset of treatments and prior to sacrifice. Spleens from each mouse were removed and weighed immediately following sacrifice. Formalin-fixed paraffin-embedded skin was sectioned and stained with H&E. Epidermal thickness measurements were completed blind to treatment, background strain, and sex as described previously [27, 28]. For each strain, CTL and IMQ groups were compared with respect to body weight change (post-treatment versus pre-treatment), epidermal thickness, and spleen weight using linear models with simultaneous evaluation of contrasts (IMQ versus CTL) based upon the exact multivariate t-distribution (R package, multcomp; function, glht; Fig. 1b–d). Comparisons among the 14 strain–sex groups for each strain and treatment were carried out using Tukey’s honest significance test with an experiment-wise type I error rate of 0.05 (R package, agricolae; R function, HSD.test). Epidermal thickness required log_10_-transformation to improve normality of the distribution, whereas body weight change and spleen weight were analyzed without transformation (Additional file 1).

### RNA-seq expression profiling

RNA-seq expression profiling was completed on randomly chosen mice (*n* = 2 for each sex, strain, and treatment). Total RNA was extracted from flash-frozen skin samples using the RNeasy Fibrous Tissue Mini Kit (Qiagen, catalog number 74704) with on-column DNase digestion (Qiagen, catalog number 79254). RNA concentrations were evaluated using the NanoDrop spectrophotometer and RNA integrity was assessed using the Agilent Bioanalyzer. RNA was further assessed for quality using the TapeStation (Agilent, Santa Clara, CA, USA) with the manufacturer’s recommended protocols. All samples had RNA integrity numbers of 8 or greater.

Samples were prepped using the Illumina TruSeq mRNA Sample Prep v2 kit (catalog numbers RS-122-2001 and RS-122-2002; Illumina, San Diego, CA, USA) following the manufacturer’s recommended protocols, with 0.1–3.0 μg of total RNA converted to mRNA by poly(A) purification. mRNA was then fragmented and copied into first strand cDNA using reverse transcriptase and random primers. The 3′ ends of cDNA were then adenylated and adapters were ligated. One adapter had a six-nucleotide barcode unique to each sample, which allowed multiple samples to be sequenced in each lane of a HiSeq flow cell (Illumina). Products were purified and enriched by PCR to create the final cDNA library. Final libraries were checked for quality and quantity by TapeStation (Agilent) and quantitative PCR using Kapa’s library quantification kit for Illumina Sequencing platforms (catalog number KK4835; Kapa Biosystems, Wilmington, MA, USA). They were then clustered on the cBot (Illumina) and sequenced six samples per lane on a 50 cycle single end HiSeq 2000 (Illumina) in high output mode (version 3 reagents).

An average of 35.5 million 50-bp reads was generated from the 56 samples (Additional file 2). The adaptor sequence was removed using the cutadapt algorithm with 5% maximum error rate (-e option) and 20-bp minimum read length (-m option) [29]. Low quality reads were removed using the cutadapt running sum quality filter with Phred33 quality threshold of 25 (-q option) and 20-bp minimum read length (-m option) [29, 30]. Reads were also quality filtered using the window-based algorithm within the FASTX-Toolkit (fastq_quality_filter), which excluded reads without phred33 score greater than 25 for at least 50% of the read length (options -q 25 -p 50) [30, 31]. The FASTX-Toolkit artifacts filter (fastx_artifacts_filter) was applied [31]. Read statistics and quality prior to mapping were assessed using the “fastx_quality_stats” utility [31] and FastQC [32].

Reads were mapped to the mouse genome (GRCm38/mm10) using TopHat2 [33] along with UCSC Genome Browser gene model annotations and Bowtie index files [34]. The TopHat2 coverage based search for junctions was disabled (--no-coverage-search) as well as mapping of reads to multiple locations (option -g 1) [33]. Bam files generated by TopHat2 were sorted, indexed, and further analyzed using samtools [35]. The number of reads mapping to each annotated gene was tabulated using HTSeq (function, htseq-count) and only reads completely and unambiguously overlapping a gene locus were included in tabulations (-m intersection-strict) [36]. Reads with alignment quality scores less than 10 were excluded (-a 10) [36]. Cufflinks was used to calculate fragments per kilobase of transcript per million mapped reads (FPKM) and associated FPKM confidence intervals [37]. Mapping quality assessments including the percentage of total mapped reads and percentage of exonic reads were generated using RSeQC [38] and RNA-SeQC [39].

### Differential expression analysis

Differential expression analysis was performed to identify genes with normalized read counts differing between IMQ- and CTL-treated samples. For each sex–strain combination, this comparison involved four samples (two IMQ-treated and two CTL-treated) and considered only genes with expression detected in at least one of the four samples. Genes were considered to have detected expression in a sample if the count per million mapped reads (cpm) was greater than 0.25 and if the FPKM lower 95% confidence interval was greater than 0. Tests for differential expression were performed using negative binomial generalized log-linear models as implemented within the R Bioconductor package edgeR [40]. To correct for library size differences among samples, gene counts were normalized using the trimmed mean of M-values (TMM) method (edgeR function, calcNormFactors) [41]. Within the negative binomial framework, dispersion parameters are estimated for each gene to quantify inflation of gene-specific variances relative to the Poisson probability distribution model [42]. These dispersions were estimated using the Cox-Reid Adjusted Profile Likelihood method (edgeR functions, estimateGLMTrendedDisp and estimateGLMTagwiseDisp) [40, 42]. Negative binomial models were then fit for each gene (edgeR function, glmFit) and *p* values for differential expression were obtained by likelihood ratio tests (edgeR function, glmLRT) [40]. Raw *p* values were adjusted for multiple hypothesis testing using the Benjamini–Hochberg method [43]. The same differential expression analysis methods were applied to identify genes differing significantly in pairwise comparisons of CTL samples from different strains. To identify genes with sex-dependent IMQ responses, negative binomial models with likelihood ratio tests were used (edgeR function, glmLRT), with full models for each gene formulated to include covariates corresponding to sex, treatment, and sex × treatment interaction, and reduced models including covariates corresponding to sex and treatment only.

### Mouse–human homology mapping

Vertebrate homology maps were downloaded from the Mouse Genome Database (http://www.informatics.jax.org) [44]. Homology maps included 16757 homologene identifiers uniquely associated with a mouse and human gene symbol. This allowed matching of mouse and human expression data based upon gene symbols sharing a common homologene identifier.

### Additional gene expression datasets

Gene expression in human psoriasis was evaluated using results from an RNA-seq meta-analysis comparing gene expression in psoriasis lesions to uninvolved skin from 44 patients (Gene Expression Omnibus (GEO) series identifiers GSE41745, GSE54456/GSE63979, GSE66511) [45]. Additional datasets incorporated into analyses include those from prior microarray studies of IMQ dermatitis in mice (GSE27628, GSE47607, GSE60804, GSE63684, GSE78057) [2, 20–22], human KCs treated with poly(I:C) (GSE21260) [46], human KCs treated with IL-17A (GSE12109, GSE24767, GSE27533, GSE32620, GSE36287, GSE52361, GSE53751), the Immunological Genome Project (IGP; GSE15907) [47, 48], macroscopically normal human skin from control subjects without skin disease (*n* = 90 subjects; GSE54456; Additional file 2), laser capture microdissected human suprabasal and basal epidermis (GSE42114) [49], regenerated human epidermis organotypic cultures (GSE52953) [50], Mediterranean spotted fever eschars (GSE32993), burn wound margins 0–3 days post-injury (GSE8056) [51], and *Leishmania braziliensis*-infected cutaneous lesions (GSE55664) [52]. Comparisons to 35 human skin diseases were made using a database of 124 expression signatures compiled and described in a previous publication [45]. IGP data were processed and analyzed to identify cell type-specific genes as described previously [47]. For all other datasets, normalized expression data were downloaded as series matrix files, with the exception of Affymetrix datasets for which raw CEL files were downloaded and normalized using robust multichip average (RMA) [53]. Tests for differential expression were performed using empirical Bayes linear models (R package, limma) [54] with raw *p* values corrected using the Benjamini–Hochberg algorithm [43].

### Real time quantitative reverse transcription PCR

The 7900HT Fast Real-time PCR system with Fast 384-well Block Module was used to carry out RT-PCR reactions (Applied Biosystems, catalog number 4309849). Pre-designed TaqMan® primer assays were purchased from Thermo Fisher Scientific (*Stat1*, Mm00439531_m1; *Irf1*, Mm01288580_m1; *Epgn*, Mm00504344_m1; *Cd3g*, Mm00438095_m1; *Cd4*, Mm00442754_m1; *Cd8a*, Mm01182107_g1; *Cyp2g1*, Mm01168480_m1; *Fosb*, Mm00500401_m1; *Aldh1l2*, Mm00463499_m1; *Atp13a5*, Mm00558722_m1; *P2rx1*, Mm00435460_m1; *Il17a*, Mm00439618_m1; *Il17b*, Mm01258783_m1; *Il17c*, Mm00521397_m1; *Il17d*, Mm01313472_m1; *Il17e*, Mm00499822_m1; *Il17f*, Mm00521423_m1; *Il1f5*, Mm00497802_m1; *Krt1*, Mm00492992_g1). 18S ribosomal RNA (*Rn18s*) was used as an endogenous control gene to normalize cycle threshold values (Applied Biosystems, catalog number Mm04277571_s1). The 2^-∆∆Ct^ method was used to calculate fold changes and estimate relative expression among samples [55]. Statistical analyses were performed using log2-scaled estimates of relative gene expression (i.e., *Rn18s* CT − Target gene CT). Group differences in relative expression were evaluated by Tukey’s honest significance test with an experiment-wise type I error rate of 0.05 (R package, agricolae; R function, HSD.test). Differential IMQ responses between strains were evaluated using linear models with coefficients corresponding to strain, sex, treatment, and strain-by-treatment interaction terms (R function, lm).

## Results

### Mouse strains differ in their baseline characteristics but macroscopic IMQ responses are consistent

Mouse back skin was treated with 62.5 mg Aldara™ (5% IMQ) or a non-toxic lanolin-derived occlusion cream (CTL) once per day for five consecutive days. Superficial erythema and scaling was similar among strains (Fig. 1a) with consistent splenomegaly (Fig. 1d) and increased epidermal thickness (acanthosis) (Fig. 1c). IMQ reduced body weight in most strains, but this was significant only in B6, DBA, FVB, and 129/SvJ mice (Fig. 1b). Unexpectedly, CTL treatment of BALB/c mice decreased body weight significantly (Fig. 1e), and consequently there was no difference between effects of CTL and IMQ on body weight in this strain (Fig. 1b). Otherwise, with or without IMQ treatment, strains varied continuously with regard to epidermal thickness, weight loss, and spleen weight (Figs. 1e–j). MOLF mice were the most disparate, with high epidermal thickness, low spleen weight, and no significant weight loss following IMQ treatment (Fig. 1e–j). Thickness of CTL-treated skin of MOLF males, for example, was increased twofold relative to FVB males (Fig. 1f).

### Genome-wide expression responses to IMQ differ prominently in MOLF males

RNA-seq was used to profile transcriptomes of 56 CTL- and IMQ-treated skin samples (7 strains × 2 sexes × 2 treatments; *n* = 2 samples per strain/sex/treatment combination). An average of 34.9 million quality-filtered reads was generated from the 56 samples, and of these 97.2% on average mapped to the mouse genome (UCSC GRCm38/mm10; Additional file 2). As expected, the proportion of mapped reads differed among strains, although at least 95% of reads on average were mapped for all strains (Additional file 2). Of mapped reads, an average of 87.6% mapped to annotated exons, and this percentage differed only slightly among strains and was no less than 86% for any genotype (Additional file 2). For each strain, expression of genes in CTL skin was correlated with that of homologous genes in normal human skin (*r*_s_ ≥ 0.816; Additional file 3a–h), and genes most highly expressed in human skin likewise had robust expression in mouse CTL skin (e.g., *Krt1*, *Krt14*, *Rpl37a*; Additional file 3i).

**Fig. 2:**
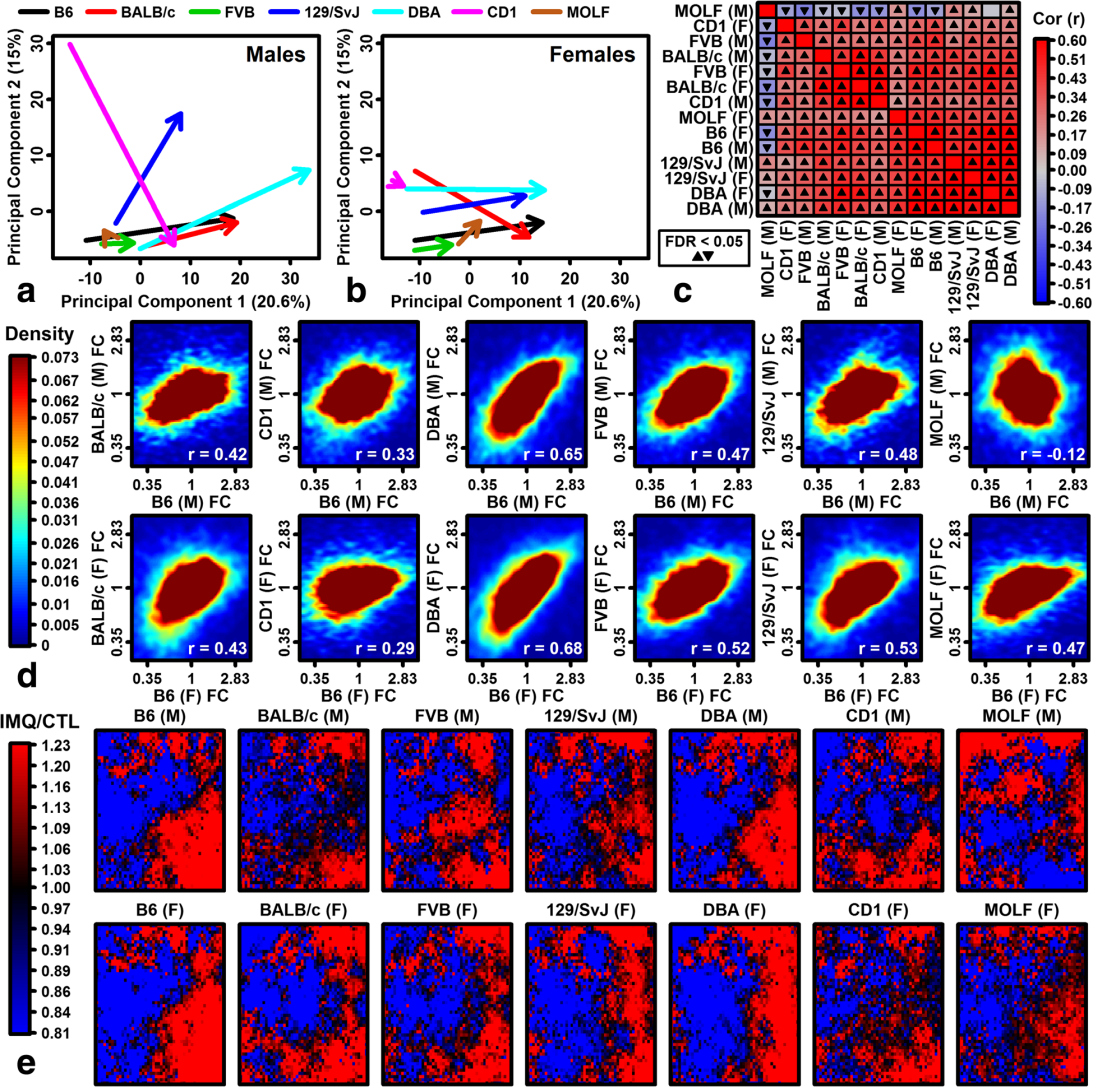
Genome-wide expression responses to IMQ. **a**, **b** Imiquimod principal component response vectors for **a** males and **b** females (*arrow start*, CTL sample average; *arrow end*, IMQ sample average). **c** Spearman correlations between fold change (FC) estimates (IMQ/CTL). **d** Comparison of FC estimates (IMQ/CTL) of each strain to B6 mice of the same sex (*top*, males; *bottom*, females; *white font*, Spearman correlation coefficient). *Colors* reflect gene density (see scale). **e** Self-organizing maps (SOMs). Genes are plotted on the same SOM for each sex/strain and the color scale denotes FC (IMQ/CTL). *F* female, *M* male

Cluster analysis of protein-coding gene expression profiles grouped IMQ samples apart from CTL samples (Additional file 4). Most expression variation was explained by mouse strain (Additional file 4e), with expression profiles of B6, BALB/c, DBA, and 129/SvJ genotypes differing from MOLF, CD1, and FVB (Additional file 4). Consistent with this, strain variation was prominent among CTL samples, with differential expression analysis suggesting that baseline expression differed most in B6 and MOLF strains (false discovery rate (FDR) <0.10; Additional file 3j). Despite such baseline expression differences, IMQ response vectors of MOLF and CD1 mice were aberrant within two-dimensional principal component space, particularly among males (Fig. 2a, b). MOLF males were most atypical and IMQ-induced expression shifts were negatively correlated with other strains (Fig. 2c, d). Consistent with this, we identified self-organizing map regions with opposite IMQ responses in MOLF males (Fig. 2e).

### Sex-specific IMQ responses differ among strains and are strongest in MOLF and FVB mice

In relative terms, most expression variation was explained by strain and treatment (IMQ versus CTL), with less variation explained by sex (Additional file 4e). Consistent with this, IMQ expression responses were positively correlated between sexes for each strain (Additional file 5). The magnitude of such correlations, however, varied considerably (0.152 ≤ *r*_s_ ≤ 0.799), with weaker correlations in MOLF and FVB strains (*r*_s_ = 0.152 and 0.267, respectively; Additional file 5). In line with this, we identified 350 and 422 genes with significant sex-by-treatment interaction effects in MOLF and FVB mice, respectively, but few or no genes with such effects in other strains (FDR <0.10; Additional file 5I). Genes with significant interaction effects in MOLF and FVB mice were largely distinct with little overlap (Additional file 5k). By applying a less stringent significance criterion (*P* < 0.05), it was possible to identify some genes with the same sex-by-treatment interaction pattern in as many as three or four strains (e.g., *Cyp2g1*, *Aldh1l2*; Additional file 5l–p). Of these, the most consistent trend was observed for a cytochrome P450-encoding gene (*Cyp2g1*), which in five of seven strains was decreased by IMQ in males but increased by IMQ in females (*P* < 0.05 for each of five strains; Additional file 5l). Consistent sex-specific IMQ responses were thus observed for some genes, although no gene showed a consistent sex-specific pattern across all seven strains (Additional file 5).

### IMQ activates immune and inflammatory gene expression in all strains but decreases expression of homeostatic genes

**Fig. 3:**
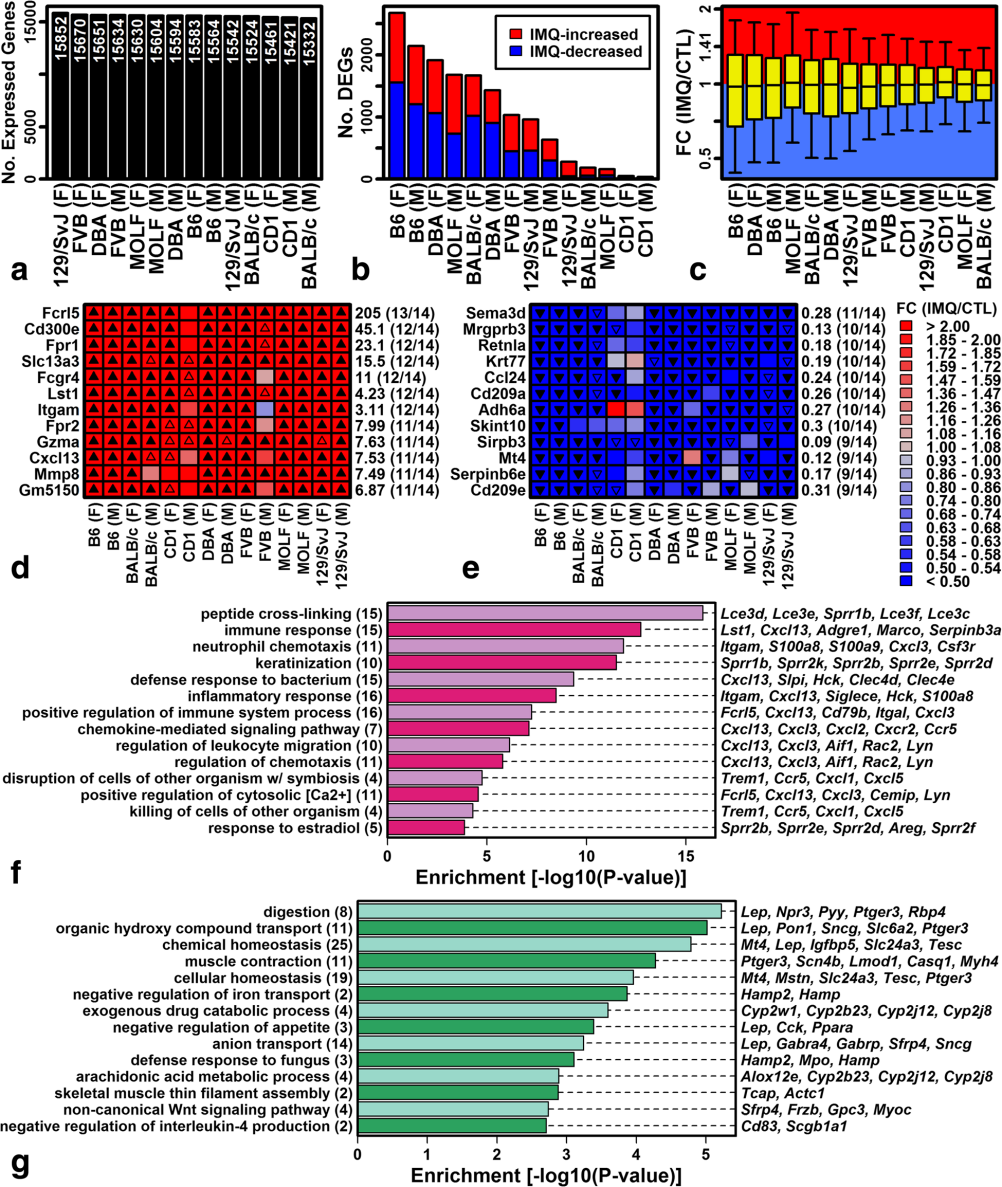
Differential expression with IMQ treatment. **a** Number of expressed protein-coding genes. **b** Number of differentially expressed genes (*DEGs*; IMQ-increased, fold change (FC) >2.0 with FDR <0.10; IMQ-decreased, FC <0.50 with FDR <0.10). **c** FC distributions (IMQ/CTL). *Boxes* outline the middle 50% of FC estimates (*whiskers*, 10th and 90th percentiles). **d**, **e** Genes most frequently increased (**d**) or decreased (**e**) by IMQ treatment. **f**, **g** Gene Ontology biological process terms significantly overrepresented among genes most frequently increased (**f**) and decreased (**g**) by IMQ. Values in *parentheses* (*left margin*) indicate the total number of IMQ-increased (**f**) or IMQ-decreased (**g**) genes associated with each term. The *right margin* lists example IMQ-increased/decreased genes associated with each term. *F* female, *M* male

The number of genes with detectable expression was similar in all strains (Fig. 3a), but the number of differentially expressed genes (DEGs) between CTL and IMQ skin varied, ranging from 35 (CD1 males) to 2676 (B6 females) (Fig. 3b). This reflected IMQ effect size differences, since the distribution of fold change estimates (IMQ/CTL) was compressed in strains with few DEGs (e.g., CD1 mice, BALB/c males, MOLF females; Fig. 3c). Among the 14 strain–sex combinations, the number of DEGs identified was negatively correlated with baseline (CTL) body weight and spleen weight (*r*_s_ = −0.42 and −0.37, respectively), although this trend was not statistically significant (*P* ≥ 0.141; Additional file 6). Genes consistently elevated by IMQ were associated with peptide cross-linking, inflammation, and immune/defense response (e.g., *Fcrl5*, *Cd300e*, *Fpr1*; Fig. 3d, f). Genes consistently decreased by IMQ (e.g., *Sema3d*, *Mrgprb3*, *Retnla*) were associated with homeostasis and transport (Fig. 3e, g).

Fold changes (IMQ/CTL) estimated by RNA-seq were usually correlated with those from previous microarray studies [2, 20–22], but the strength of such correlations varied by strain and among array experiments (Additional file 7a–d). Correspondence was weaker relative to one microarray study utilizing ear skin, rather than back skin, suggesting that site of application is influential (Additional file 7b). Although RNA-seq/microarray FCs were correlated, we nonetheless identified 719 DEGs induced by IMQ in B6 and/or BALB/c mice (the previously studied strains), which had not reached the same significance thresholds in microarray experiments (fold change >2.0 with FDR <0.10; Additional file 7c). Similarly, we identified 1407 IMQ-repressed DEGS not identified previously by microarray (B6 and/or BALB/c; fold change <0.50 with FDR <0.10; Additional file 7c). Key cytokine-encoding genes were among those identified by RNA-seq but not in prior microarray experiments (e.g., *Il17c*, *Il21r*, *Il18*, *Il20*; B6 and/or BALB/c; Additional file 7e, f).

### Correspondence with human psoriasis varies among mouse strains and is non-significant for genes decreased by IMQ in MOLF males

Genes altered by IMQ in B6 mice were previously shown to exhibit similar expression shifts in human psoriasis lesions (*r* = 0.21) [2]. For 12 of 14 sex–strain combinations, fold changes (IMQ/CTL) were even more strongly correlated with psoriasis (lesional/uninvolved skin) than estimated previously [2], with an estimated correlation of 0.45 in B6 males (Additional file 8a). The correlation magnitude, however, varied considerably and ranged from 0.05 (MOLF males) to 0.45 (B6 males) (Additional file 8a). There was no significant tendency for psoriasis-decreased DEGs to be similarly repressed by IMQ in MOLF males, although this was the case for each of the 13 other sex–strain combinations (Additional file 8c). Of the 50 genes most strongly elevated in psoriasis lesions, none were significantly induced by IMQ in all strains, although we could identify some induced in most except MOLF males (e.g., *Serpin3a*, *Serpin3d*, *Klk9*, *Klk13*; Additional file 8d). Similarly, none of the 50 genes most strongly repressed in psoriasis lesions were significantly repressed by IMQ in all strains (Additional file 8e). Notably, some genes strongly decreased in psoriasis lesions were elevated by IMQ in most strains (*Syt8*, *Cdhr1*, *Cntn2*; Additional file 8e). The IMQ/psoriasis correspondence, therefore, was better than estimated previously using microarray [2], but nonetheless imperfect and dependent upon strain and sex.

### Anti-viral and mitotic pathways activated in psoriasis lesions have strain-dependent responses to IMQ in mice

**Fig. 4:**
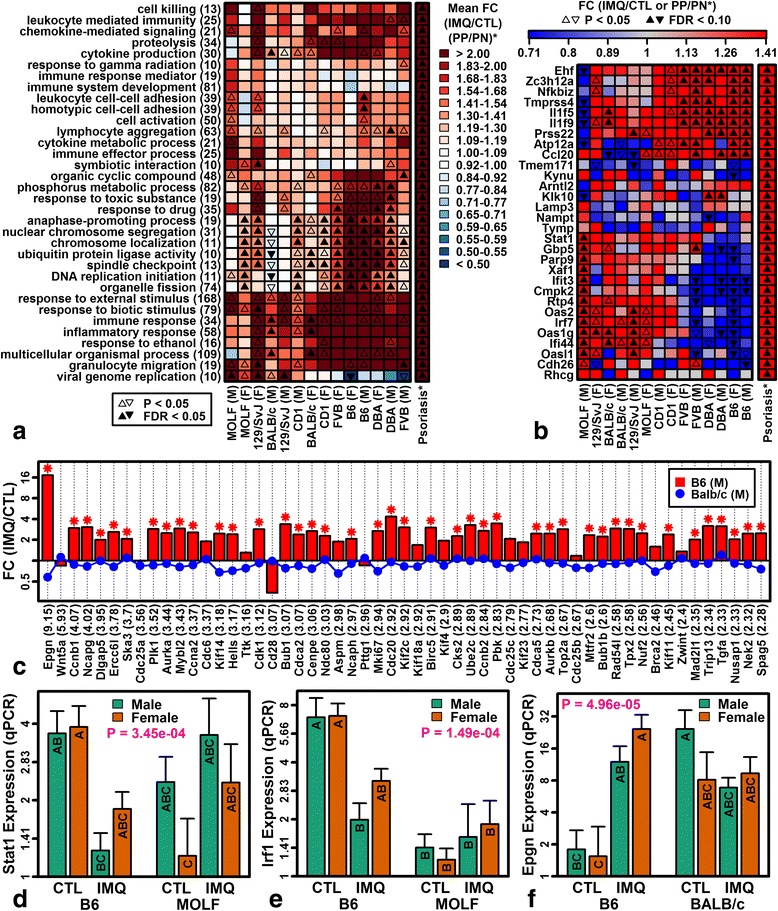
Gene Ontology (GO) biological process (BP) terms enriched among psoriasis-increased DEGs and correspondence with IMQ responses. **a** Psoriasis-enriched GO BP terms. The heatmap shows average IMQ responses of ten representative mouse homologs for each GO BP term. Chosen genes are homologous to the ten human genes associated with the listed term for which expression was most strongly elevated in psoriasis lesions (i.e., lowest *p* value). **b** Psoriasis-increased DEGs induced by poly(I:C) in cultured human KCs. **c** Genes associated with “organelle fission” (GO:0048285) (*FDR <0.10, IMQ versus CTL). **d**–**f** RT-PCR analysis of *Stat1* (**d**), *Irf1* (**e**), and *Epgn* (**f**) expression (*n* ≥ 4 per strain/sex/treatment group; *n* = 38–40 mice total). Groups without the same letter differ significantly (*P* < 0.05, Tukey honest significant difference. *Error bars* represent standard error of the mean; *p* values strain-by-treatment interaction effect). *Rn18s* was used as an endogenous control to estimate relative gene expression. *F* female, *FC* fold change, *M* male, *PN* uninvolved skin from psoriasis patients, *PP* lesional skin from psoriasis patients, *qPCR* quantitative PCR

We identified Gene Ontology (GO) biological process (BP) terms significantly overrepresented among genes with elevated expression in psoriasis lesions and evaluated whether corresponding mouse genes were similarly altered by IMQ (Fig. 4a). In most strains, psoriasis-increased genes associated with DNA replication and mitosis were induced by IMQ (Fig. 4a). BALB/c males, however, were an exception and in many cases such genes were not altered or decreased (e.g., *Ccnb1*, *Ncapg*; Fig. 4c). Consistent with this, there was a trend towards increased expression of epithelial mitogen (*Epgn*) with IMQ treatment in B6 but not BALB/c mice (Fig. 4f; *P* = 4.96 × 10^−5^, strain-by-treatment interaction).

IMQ induced expression of psoriasis-increased genes associated with immune and inflammatory response (Fig. 4a). However, psoriasis-increased genes associated with viral genome replication were induced by IMQ in MOLF, 129/SvJ, and BALB/c strains but not B6, DBA, and FVB (Fig. 4a). We thus examined genes elevated in psoriasis lesions and induced in human KCs following treatment with poly(I:C) (e.g., *Ehf*, *Nfkbiz*, *Zc3h12a*; Fig. 4b) [46]. These genes were variably altered by IMQ in mouse strains, with opposite IMQ responses depending on strain (Fig. 4b), and several encoded transcription factors mediating response to viral RNA and interferon (e.g., *Stat1* and *Irf1*; Fig. 4d, e).

Genes with decreased expression in psoriasis lesions were frequently associated with homeostasis, transport, metabolism, and development (Additional file 9a), partially consistent with the core set of genes downregulated by IMQ across mouse strains (Fig. 3g). Many homeostatic and transport genes most strongly decreased in psoriasis were similarly repressed by IMQ, although a number of these were increased in MOLF males (Additional file 9b, c).

### IMQ induces stronger activation of the IL-17A pathway in B6 compared to BALB/c mice

**Fig. 5:**
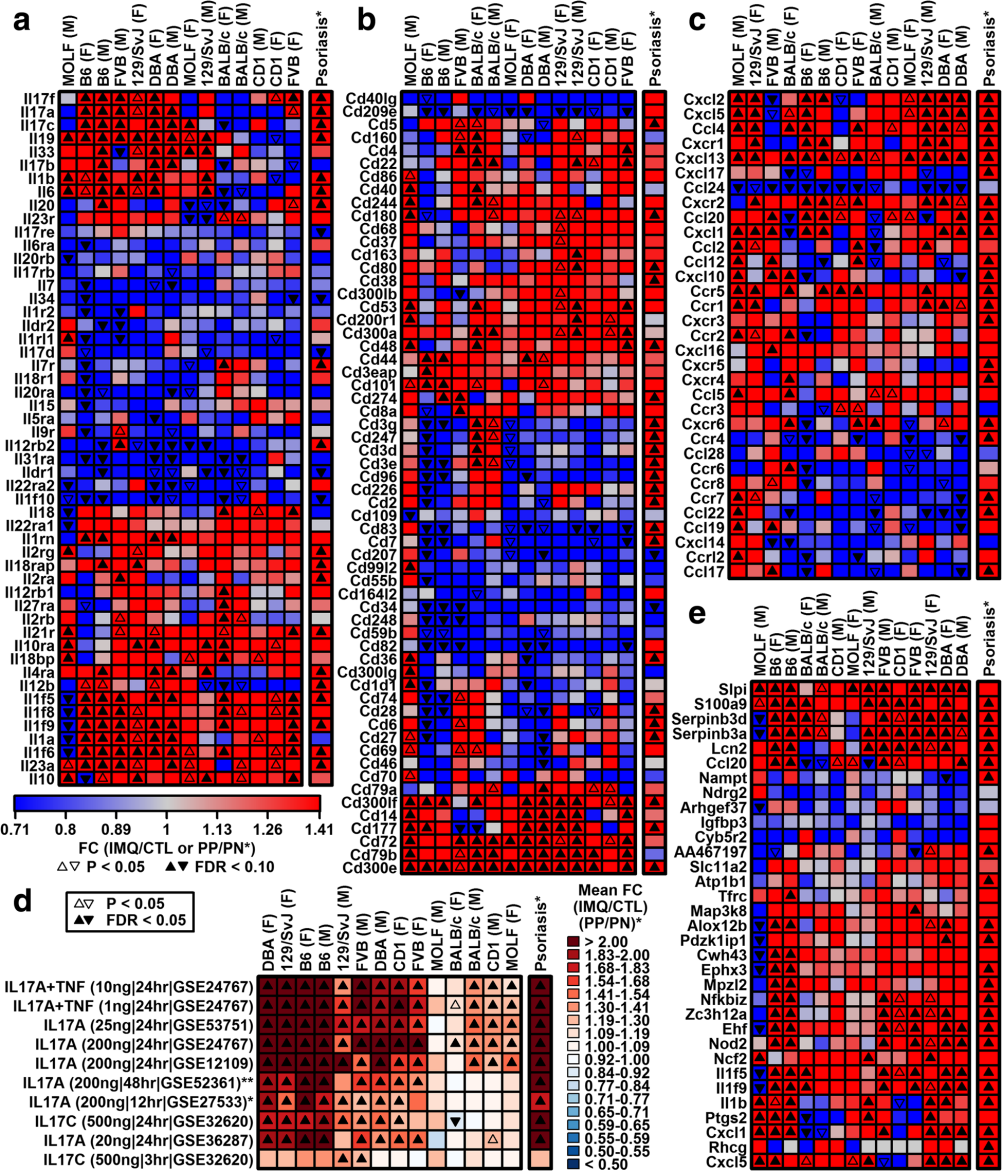
Cytokine, chemokine, and cluster of differentiation gene expression. **a**–**c** Genes encoding cytokines (**a**), cluster of differentiation proteins (**b**), and chemokines (**c**). **d** Genes induced by IL-17 family cytokines (human KCs). Heatmap colors depict average IMQ response of 50 genes most strongly induced in each experiment (*left margin*; *HaCaT; **reconstituted epidermis; cytokine concentration and exposure time shown in *parentheses*). **e** Genes most frequently induced in six experiments from **d** in which KCs were treated with IL-17A alone. *F* female, *FC* fold change, *M* male, *PN* uninvolved skin from psoriasis patients, *PP* lesional skin from psoriasis patients

Psoriasis is a cytokine-driven disease and manipulation of cytokine-encoding genes impacts the mouse IMQ phenotype [4]. We thus evaluated expression of genes encoding cytokines and chemokines, as well as genes associated with cluster of differentiation proteins on the surface of immune cells (Fig. 5). Cytokines and chemokines with increased expression in human psoriasis were often similarly elevated by IMQ, but there was no case in which such genes were similarly and significantly altered in both sexes and all strains (Fig. 5a, c). Expression of *Il1f5*, *Il1f6*, *Il1f8*, and *Il1f9* was elevated in human psoriasis and most strains except MOLF males (Fig. 5a). Expression of IL-17 family cytokines, including *Il17a*, *Il17b*, *Il17c*, and *Il17f*, was differentially altered by IMQ among strains, with IMQ-increased expression in B6 mice and IMQ-decreased (or unaltered) expression in BALB/c mice (Fig. 5a). These trends were confirmed by RT-PCR (*n* = 5 per strain/sex/treatment group; *P* ≤ 0.037, strain-by-treatment interaction; Additional file 10). Consistent with this, genes induced by IL-17A in cultured human KCs were more strongly elevated by IMQ in B6 relative to BALB/c mice (Fig. 5d). Of 33 genes induced by IL-17A in human KCs, 21 were induced by IMQ in B6 mice, but only six were induced by IMQ in BALB/c mice (Fig. 5e).

### Innate immune responses to IMQ are consistent among strains but T lymphocyte expression differs by strain

**Fig. 6:**
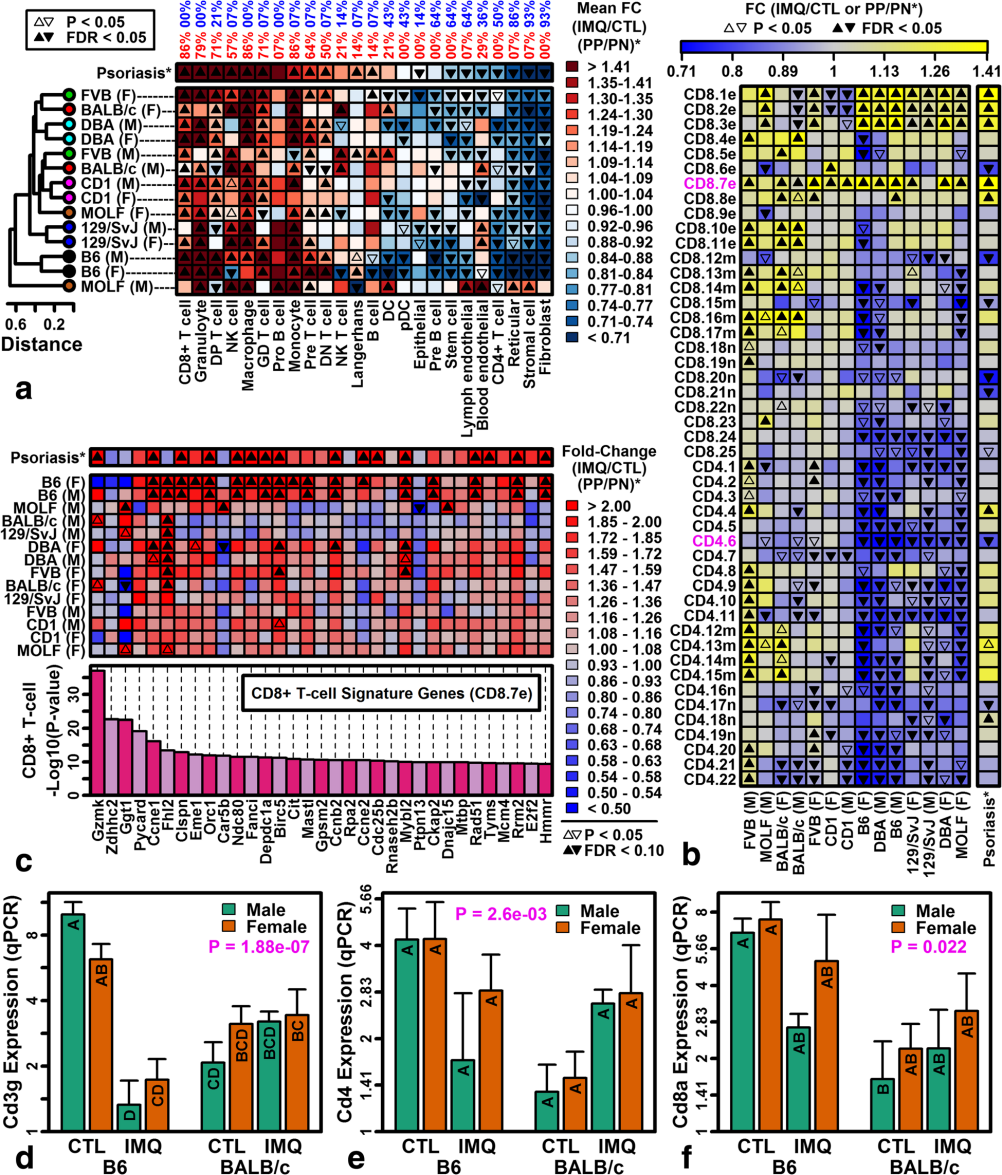
Effects of IMQ on the expression of cell type-specific genes (Immunological Genome Project). **a** Heatmap colors depict average IMQ responses of 100 signature genes specifically expressed by each cell population (*bottom margin*; percentage of sex–strain combinations with significantly increased (*red*) or decreased (*blue*) signature gene expression; FDR <0.05, Wilcoxon rank sum test). **b** Replicate CD4+ and CD8+ T cell populations. Heatmap colors depict average IMQ responses of 100 signature genes for each T-cell population (*left margin*: *n* = naïve T cell, *m* = memory T cell; *e* = effector T cell; *magenta font* indicates representative populations shown in **a** and **c**). **c** CD8.7e signature genes (GEO samples GSM538387, GSM538388, and GSM605897). *P* values (*vertical axis*) quantify the degree of cell type-specific expression for each gene (CD8.7e versus other IGP cell types, Wilcoxon rank sum test). **d**–**f** RT-PCR analysis of *Cd3g* (**d**), *Cd4* (**e**), and *Cd8* (**f**) expression (*n* = 5 per strain/sex/treatment group; *n* = 40 mice total). Groups without the same letter differ significantly (*P* < 0.05, Tukey honest significant difference; *error bars* represent standard error of the mean; *p* values, strain-by-treatment interaction effect). *Rn18s* was used as an endogenous control to estimate relative gene expression. *F* female, *FC* fold change, *M* male, *PN* uninvolved skin from psoriasis patients, *PP* lesional skin from psoriasis patients, *qPCR* quantitative PCR

Activation of cytokine pathways by IMQ may differ in mouse strains due to disparities in the abundance of infiltrating immune cells. We thus used microarray data from the Immunological Genome Project (IGP) to identify signature genes specifically expressed by sorted immune cell populations [47, 48] and evaluated whether expression of such genes is systematically altered in IMQ-treated skin and human psoriasis (Fig. 6).

Both IMQ dermatitis and psoriasis showed increased expression of immune cell-expressed genes and decreased expression of genes expressed by resident cell types (e.g., fibroblasts and epithelial and endothelial cells; Fig. 6a). Consistent with human psoriasis, IMQ increased expression of genes expressed by innate immune cells (e.g., macrophages, monocytes, and granulocytes; Fig. 6a). On the other hand, Langerhans and dendritic cell (DC)-expressed genes were weakly altered in human psoriasis but variably affected by IMQ in different strains (Fig. 6a).

Genes encoding CD3 antigen were repressed by IMQ in B6 mice but not BALB/c mice, suggesting strain-specific effects of IMQ on T-lymphocyte abundance (Figs. 5b and 6d). Consistent with this, signature genes expressed by CD4+ T-cell populations were decreased in B6, DBA, and 129/SvJ IMQ phenotypes but unaltered or elevated in the FVB and BALB/c phenotypes (Fig. 6b). In agreement with this, *Cd4* expression was decreased by IMQ in B6 but not BALB/c mice (Fig. 6e; *P* = 2.6 × 10^−3^, strain-by-treatment interaction). Genes expressed by most CD8+ T-cell populations followed similar trends and, indeed, the *Cd8a* expression pattern mirrored *Cd4* (Fig. 6f; *P* = 0.022, strain-by-treatment interaction). However, genes expressed by effector CD8+ T cells harvested from virus-infected mice (CD8.7e) were elevated in nearly all IMQ phenotypes as well as human psoriasis, with stronger increases in B6 mice compared to other strains (Fig. 6a–c).

### IMQ treatment of MOLF males induces TLR gene expression but downregulates KC differentiation genes localized to suprabasal epidermis

**Fig. 7:**
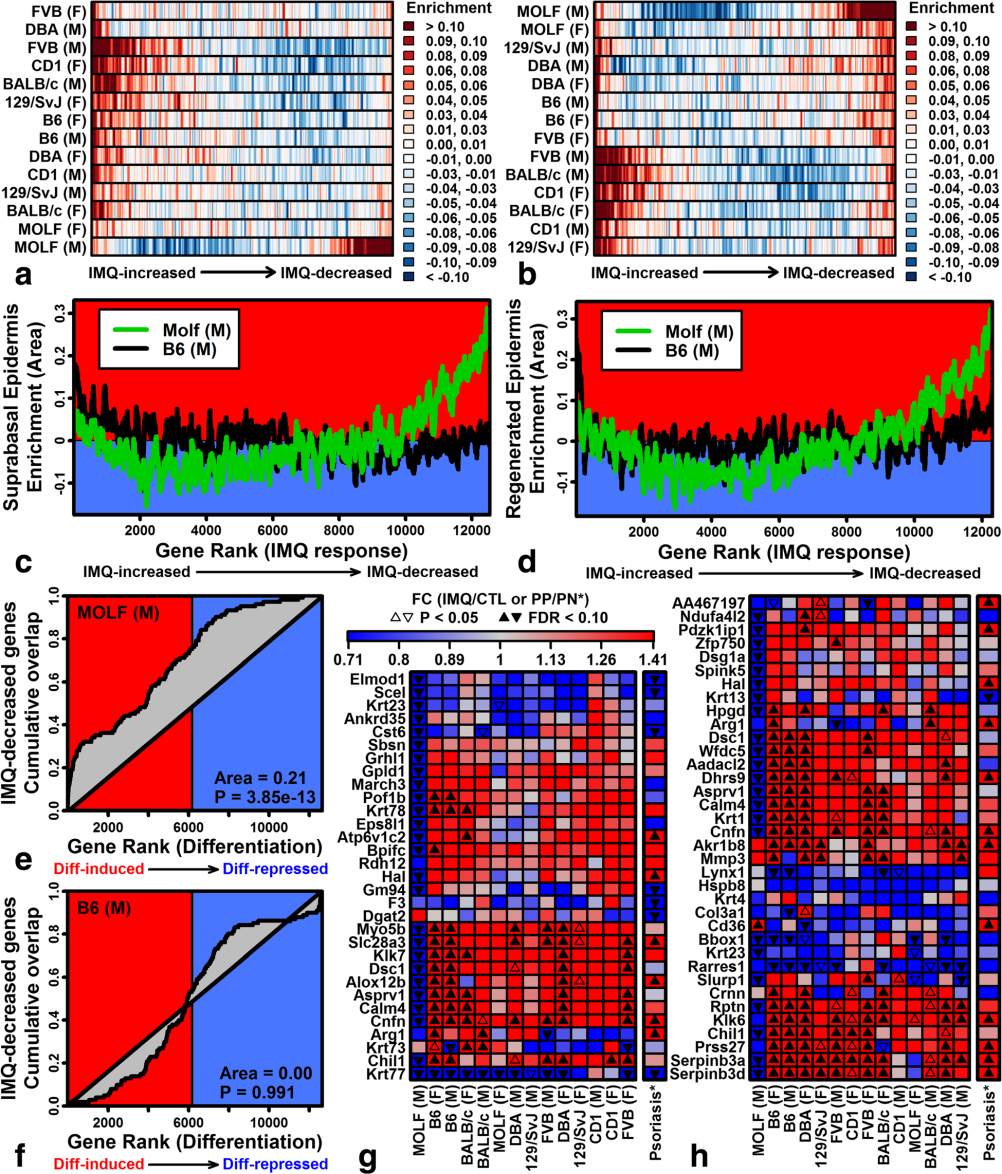
IMQ treatment of MOLF males downregulates KC differentiation genes localized to suprabasal epidermis. **a** Suprabasal versus basal epidermis (GSE42114; sliding window analysis). Genes were ranked based upon their IMQ response and windows of 100 genes were tested for enrichment in suprabasal epidermis (*red*, suprabasal-increased; *blue*, suprabasal-decreased). **b** Human epidermal regeneration (GSE52651; sliding window analysis). Genes were ranked based upon their IMQ response and windows of 100 genes were tested for enrichment in regenerated epidermis (day 7 versus day 0; *red*, differentiation-increased; *blue*, differentiation-decreased). **c** Suprabasal versus basal epidermis. Enrichment statistics in MOLF and B6 males. **d** Human epidermal regeneration (day 7 versus day 0). Enrichment statistics in MOLF and B6 males. **e**, **f** Gene set enrichment analysis for the top 100 IMQ-decreased genes in MOLF males (**e**) and B6 males (**f**). Cumulative overlap (*vertical axis*) is shown between IMQ-decreased genes and genes ranked according to their expression in suprabasal versus basal epidermis (*horizontal axis*). **g**, **h** Genes most strongly elevated in suprabasal (**g**) versus basal (**h**) epidermis and in regenerated epidermis (day 7 versus day 0 cultures). *F* female, *FC* fold change, *M* male, *PN* uninvolved skin from psoriasis patients, *PP* lesional skin from psoriasis patients

The MOLF male expression response to IMQ was negatively correlated with other strains (Fig. 2c, d). Genes specifically elevated by IMQ in MOLF males were associated with organic acid metabolic process, brown fat cell differentiation, type I interferon, and lipid storage (e.g., *Ifit1*, *Ifit3*, *Lep*, *Adipoq*; Additional file 10a). Genes specifically repressed by IMQ in MOLF males included IL-1 family cytokines (e.g., *Il1a*, *Il1f5*, *Il1f6*, *Il1f8*, *Il1f9*; Fig. 5a; Additional file 11b), as well as other genes associated with epidermis development, peptide cross-linking, and skin barrier establishment (e.g., *Krt1*, *Serpin3a*, *Serpin3b*; Additional file 11b, f). Consistent with this, genes decreased by IMQ in MOLF males were elevated in suprabasal versus basal epidermis (Fig. 7a, c) [49], as well as during 7 days of organotypic human epidermal regeneration (Fig. 7b, d) [50]. This was not the case for other strains, however (Fig. 7a–f). Nearly all of the genes most strongly elevated in suprabasal epidermis (or during epidermal regeneration) were decreased by IMQ in MOLF males but not other strains (e.g., *Grhl1*, *Krt1*, *Krt78*, *Klk7*, *Dsc1*; Fig. 7g, h). Using RT-PCR, we confirmed that *Il1f5* and *Krt1* show different expression responses to IMQ in B6 and MOLF mice, with slightly decreased expression in MOLF males in contrast to strongly elevated expression in the B6 strain (Additional files 11c and 10d; *P* < 0.011, strain-by-treatment interaction).

Previous work has shown that MOLF mice exhibit unique responses to TLR agonists, such as lipoteichoic acid, poly(I:C), lipopolysaccharides, cytosine guanine dinucleotide (CpG), and resiquimod [18, 56, 57]. A set of 101 TLR-associated genes compiled by the Kyoto Encyclopedia of Genes and Genomes (KEGG) [58] was disproportionately elevated by IMQ in all strains, including MOLF males and females (Additional file 12; KEGG pathway mmu04620). *TLR1* expression was significantly elevated by IMQ in MOLF males and females, and MOLF males showed robust elevation of other TLR upstream components (*TLR2*, *TLR6*, *CD14*, *PI3K*), as well as mRNAs encoding downstream transcription factors (STAT1, IRF7) and cytokines (tumor necrosis factor (TNF)-α, IL-1β, IL-6, RANTES, MIP1-α, MIP-1β) (Additional file 12). Among all strains, expression of TLR-associated genes was most strongly elevated in 129/SvJ mice but most weakly elevated in CD1 mice (Additional file 12).

### Genes altered by IMQ overlap significantly with those altered in other human skin conditions besides psoriasis (wounding, acne, eschars, infection)

**Fig. 8:**
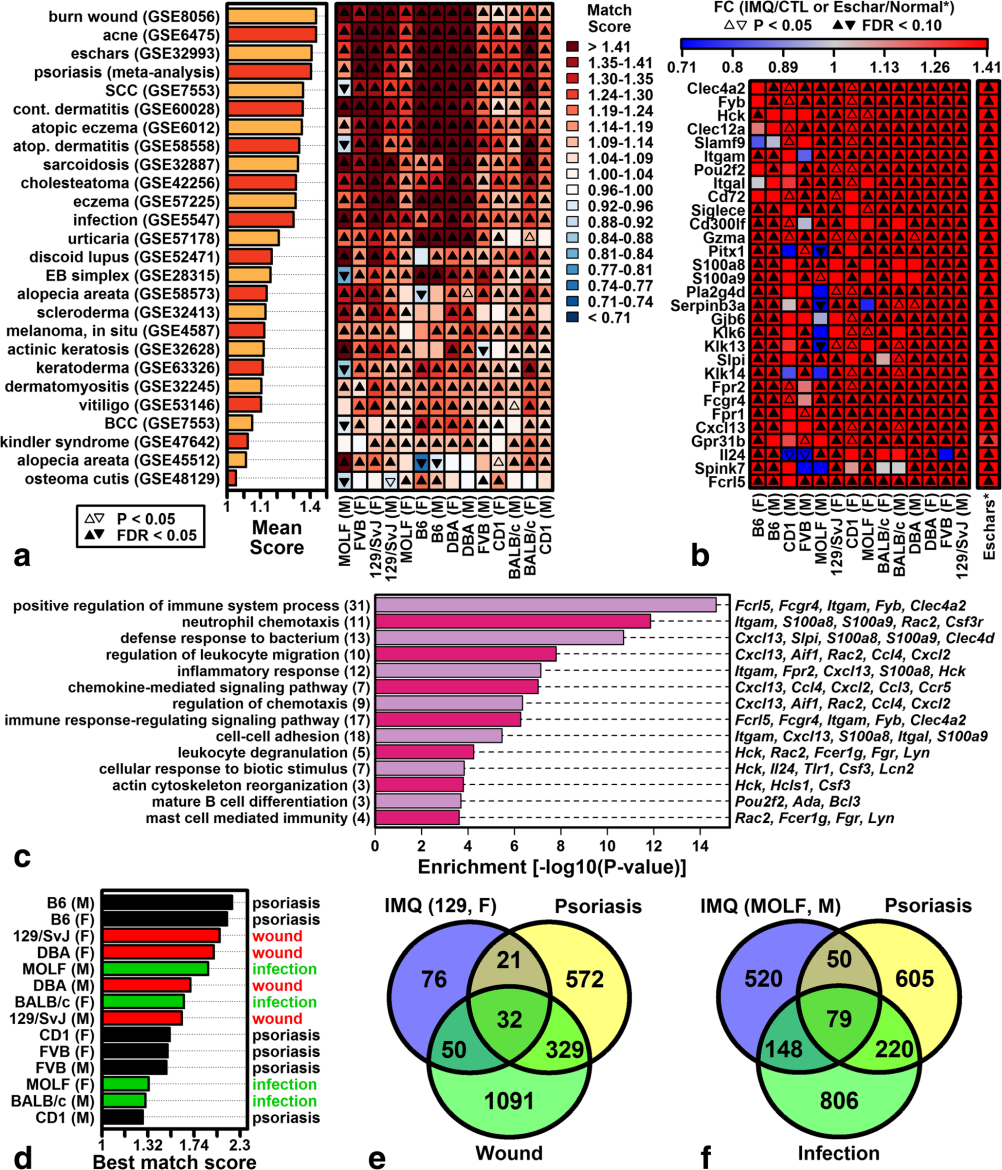
Genes altered by IMQ overlap significantly with those altered in other human skin conditions besides psoriasis (eschars, acne, infection, wounding). **a** Match scores. Scores represent the average fold change (FC) of the 200 genes most strongly increased in each disease (IMQ/CTL) and the 200 genes most strongly decreased in each disease (CTL/IMQ). Diseases are ranked by average score across IMQ phenotypes. **b** Genes increased in Mediterranean spotted fever (MSF) eschars and IMQ phenotypes. **c** GO BP terms enriched among genes increased in MSF eschars and IMQ phenotypes. **d** The highest score is shown for each sex–strain combination. **e** 129/SvJ females. Overlap of genes increased in IMQ dermatitis, burn wounds, and psoriasis lesions (FDR <0.10 in each case). **f** MOLF males. Overlap of genes increased in IMQ dermatitis, *L. braziliensis*-infected cutaneous lesions, and psoriasis lesions (FDR <0.10 in each case). *F* female, *FC* fold change, *M* male

Gene expression patterns in psoriasis overlap considerably with those in other human skin diseases [45]. We therefore considered whether expression responses to IMQ in mice were consistent with other human skin diseases besides psoriasis (Fig. 8).

IMQ expression responses were compared to 124 expression signatures derived from microarray studies of 35 skin diseases [45]. A match score was calculated for each disease to quantify whether genes most strongly increased/decreased in disease lesions were similarly altered by IMQ (Fig. 8a). On average across all strains, match scores for wounding (GSE8056), acne (GSE6475), and Mediterranean spotted fever (MSF) eschars (GSE32993) were higher than those for psoriasis (Fig. 8a), although significant correspondence was observed for several human skin diseases (e.g., squamous cell carcinoma, contact dermatitis, atopic eczema, atopic dermatitis; Fig. 8a). Genes elevated by IMQ and in MSF eschars were associated with neutrophil chemotaxis, defense response to bacterium, leukocyte migration, and inflammatory response (e.g., *Itgam*, *S100a8*, *S100a9*, *Cxcl13*; Fig. 8b, c). For six of the 14 sex–strain combinations, the single highest match score was obtained for psoriasis (Fig. 8d), but in other cases IMQ responses were most similar to another disease (e.g., wounding and infection; Fig. 8d). Among 179 genes elevated by IMQ in 129/SvJ females, for example, 82 were correspondingly increased in burn wound margins (0–3 days post-injury) [51], whereas fewer (53) were elevated in psoriasis lesions (Fig. 8e). Likewise, among 797 genes elevated by IMQ in MOLF males, 227 were elevated in *L. braziliensis*-infected cutaneous lesions [52], but only 129 were elevated in psoriasis lesions (Fig. 8f).

### The psoriasis-specific expression signature varies among strains but is most closely aligned with IMQ responses in B6 mice

All mouse strains showed expression responses to IMQ that overlapped significantly with other human skin diseases besides psoriasis (Fig. 8a). Compared to other strains, however, the B6 response to IMQ was most consistent with psoriasis (Additional file 8a), and for this strain no other disease matched IMQ responses more closely than psoriasis (Fig. 8d).

We next considered genes with a high psoriasis specificity index (PSI) identified recently from a large-scale comparison of expression data from human skin diseases [45]. Such genes provide a good basis for evaluation of psoriasis mouse models since they are significantly altered in psoriasis lesions but not similarly altered in lesions from other skin diseases [45]. Many such genes did not show psoriasis-like expression responses to IMQ in any strain (e.g., *Atp1b1*, *Dnah2*, *Slc8a1*, *Prkcb*; Additional file 13). Some psoriasis-specific genes, however, were similarly altered by IMQ, although these IMQ responses varied among strains (Additional file 13). The response in B6 mice best paralleled expression patterns specific to human psoriasis (Additional file 13b, e). Only B6 mice, for example, showed significantly decreased expression of *Fcer2*, *Cspg4*, *Lsp1*, and *Aldh1b1* with IMQ treatment to recapitulate expression shifts unique to psoriasis lesions. In contrast, IMQ responses in MOLF males and 129/SvJ mice were least consistent with psoriasis-specific expression shifts, and in some cases IMQ altered expression in the direction opposite to that observed in psoriasis lesions (Additional file 13b, e).

## Discussion

The IMQ model of psoriasiform dermatitis has been used in more than 200 mouse studies as a convenient tool to probe mechanisms underlying psoriasis vulgaris. The importance of strain and sex on IMQ response has never been systematically investigated, but may impact conclusions and interpretation of experimental findings. Indeed, inattention to strain and sex may underlie conflicting results among laboratories, possibly even impeding translation from mouse to humans [10, 11, 59]. Understanding these factors can therefore bolster reproducibility of mechanistic studies [13]. Using RNA-seq, we showed that strain and sex influence correspondence between Aldara (5% IMQ)-induced dermatitis and human psoriasis, suggesting that phenotypes developing on alternative backgrounds model the human disease to differing extents. Strain differences were not peripherally associated with psoriasis disease mechanisms but related to key aspects of the pathogenesis (e.g., KC differentiation, T-cell abundance, and IL-17A and type I interferon responses). The transcriptional IMQ signature, moreover, did not uniquely correspond to human psoriasis but was consistent with diverse inflammatory skin conditions. Consequently, the human disease best matching IMQ expression responses varied by strain, with psoriasis correspondence strongest in B6 mice. Surprisingly, responses in four strains were more consistent with wounding or infection (e.g., BALB/c, 129/SvJ, DBA, MOLF).

The formation and development of psoriasis lesions depend upon a dynamic cytokine network that involves a multitude of interacting cell types and mediators. At present, however, biologic therapies directed against only a limited number of cytokines have proven clinically effective, including TNF, IL-17A, and IL-23 [60–62]. Of these, IL-17A may represent the most pivotal “hub” within the cytokine network coordinating plaque development, since TNF and IL-23 appear to positively regulate IL-17A production, in part through their influence on Th17 differentiation [3]. In this study, responses of IL-17 family cytokines to IMQ varied, with differing response patterns in the two most commonly studied strains (B6 and BALB/c). In B6 mice, IMQ increased expression of *Il17a*, *Il17b*, *Il17c*, and *Il17f*, partially consistent with human psoriasis, but in the BALB/c strain such genes were unaltered by IMQ (Additional file 10). Stronger elevation of IL-17 family gene expression in B6 mice was concurrent with heightened epidermal thickness and mitotic gene expression, along with increased expression of genes expressed by CD8+ T cells, granulocytes, and ɣδ T cells (Fig. 6a). These findings conflict with a previous study reporting that *Il17a* expression was induced by IMQ in both B6 and BALB/c mice, although with a faster response in BALB/c (2 days) compared to B6 mice (3–4 days) [3]. Since IMQ responses were evaluated after a longer time period in this study (6 days), however, it is unlikely that our results solely reflect delayed *Il17a* response to IMQ in B6 mice. In previous studies, manipulation of IL-17 family cytokines appears to have only modest effects on IMQ dermatitis, with the largest effects observed in mice lacking IL-17 F [3, 5, 63, 64]. The main implication of our findings is that the commonly studied B6 and BALB/c mice cannot be regarded as interchangeable in such work and disparate responses in these strains may impact conclusions related to this important cytokine family.

Topical IMQ was originally developed as a means to induce anti-viral responses and type I interferon in animal models [4]. It is therefore surprising that genes associated with viral genome replication and poly(I:C) were among the most variably altered by IMQ, with key pathway components activated in some strains (MOLF, 129/SvJ, BALB/c) and repressed in others (B6, DBA, FVB) (Fig. 4a, b). In the latter group, IMQ also led to stronger declines in the expression of genes specifically expressed by plasmacytoid dendritic cells (pDCs), which are considered to be the main source of IFN-α in IMQ dermatitis [65]. Our results may reflect strain disparities in pDC abundance, since prior studies have demonstrated increased pDC numbers in 129/SvJ and BALB/c mice compared to the B6 strain (blood, spleen, and lymph nodes), along with reduced ability of B6 mice to produce IFN-α following viral stimulation [66, 67]. The exact role of pDCs and type I interferon in IMQ dermatitis is complex with conflicting evidence. One study reported that pDC depletion in B6 mice has little impact on IMQ dermatitis with similar phenotypes in wild-type mice and *Ifnar1*-KO mice (i.e., mice deficient for receptor 1 of type I IFNs) [65]. A more recent study, however, indicates that erythema, epidermal proliferation, and splenomegaly are reduced in IMQ dermatitis of *Ifnar1*-KO mice (B6) [68]. Given that B6 mice exhibit relatively weak type I interferon IMQ responses (Fig. 4a, b), the IMQ phenotype may be attenuated more substantially by *Ifnar1* ablation on other genetic backgrounds (e.g., MOLF, 129/SvJ, and BALB/c).

MOLF/EiJ mice are representatives of strains commonly referred to as “wild-derived”, which have a unique genetic history with divergence from classic inbred strains occurring more than one million years ago [69]. MOLF mice thus represent a reservoir of genetic diversity absent from common laboratory strains, providing a rich genetic resource for discovering novel gene functions based on previously untested allelic variants. It was of interest to evaluate IMQ dermatitis in MOLF mice, since previous studies have identified MOLF genetic variants impacting Toll-like receptor (TLR) signaling, including alleles associated with *Tlr5* [57], *Irak1bp1* [56], *Irak2* [70], *Irak2c* [71], and *Cyld* [72]. These variants appear to alter immune responses of MOLF mice to several stimuli, such as lipoteichoic acid, poly(I:C), lipopolysaccharide, and schistosome infection [18, 56, 70]. IMQ is a TLR7 agonist and previous work has identified TLR7 expression in a subpopulation of mouse interfollicular epidermal keratinocytes [73]. In our study, IMQ was poorly tolerated by MOLF mice and two of ten IMQ-treated mice died prior to completing the 5-day treatment (see “Methods”). A previous study has demonstrated elevated IL-6 production by MOLF macrophages following TLR stimulation [56], which may have contributed to the mortality we observed [3]. Among MOLF mice surviving treatment, TLR-associated genes did not exhibit aberrant IMQ responses compared to other strains (Additional file 12). Rather, we identified an aberrant epidermal response suggestive of defective KC differentiation and barrier formation (Fig. 7). These epidermal effects may be secondary to TLR-dependent and TLR-independent acute immune responses in MOLF mice, and consistent with this, genes associated with IFN-α were uniquely upregulated by IMQ in MOLF males (e.g., *Ifit1*, *Ifit2*, *Ifit3*; Additional file 11e). Activation of these and other immune responses in MOLF mice may promote NALP3 inflammasome activation and apoptosis within the epidermis [74]. Future work will be needed to address these possibilities and identify the locus (loci) responsible. These findings, however, demonstrate the potential for MOLF mice, and possibly other wild-derived strains, to exhibit cutaneous responses differing from common laboratory strains. Further analysis of such mice and their unique allelic diversity may be valuable to other areas of experimental dermatology, where established observations may be based largely upon classic inbred strains (e.g., B6 and BALB/c).

This study is the first to report results from RNA-seq-based comparisons of IMQ expression responses among multiple mouse strains. Our findings are novel and informative, but we note six study limitations. First, we have focused on gene expression responses to IMQ. This approach is objective and quantitative, but other aspects of IMQ dermatitis not considered in our study, such as histological characteristics and drug treatment responses, represent additional criteria for comparing IMQ phenotypes among strains and to human skin diseases [1]. Second, this study evaluated Aldara (5% IMQ) at a topical dose of 62.5 mg and analyzed the resulting phenotype after 5 days of treatment, based on our observation that the cutaneous phenotype has reached steady state and is fully developed one day after the final application (day 6). Given this protocol, we have not examined early inflammatory responses 24–48 hours post-treatment, which may also differ depending upon strain and sex [74]. Along these lines, we cannot exclude the possibility that strain and sex differences we’ve identified reflect kinetic differences in IMQ response, potentially associated with strain variation in absorption [3]. Third, to ensure comparability, we used a common treatment protocol in all experiments, although this same protocol may not be ideal for each strain. A lower dose or less prolonged treatment, for example, may be appropriate for MOLF mice. Potentially, an IMQ protocol specifically tailored to each strain would achieve greater uniformity of IMQ responses or better transcriptional correspondence to human psoriasis. Fourth, this study focused exclusively on the IMQ dermatitis developing on back skin. Ear skin is another common application site and may yield responses different from those described here [4]. Fifth, our analyses have focused on IMQ responses of each strain, i.e., the expression difference between IMQ and CTL samples. Notably, however, baseline gene expression as measured in CTL samples also varied among strains and may contribute to IMQ response differences (Additional file 3). Sixth, control experiments in this study were performed using a benign lanolin-derived occlusion cream, which lacks the isostearic acid vehicle component of the Aldara preparation. Some effects we’ve attributed to IMQ may thus alternatively be due to isostearic acid vehicle, although in prior work vehicle effects have been limited to early responses (<24 h) [74] rather than the day 6 phenotypes examined here.

Mechanistic studies of human psoriasis have been hindered by the lack of an animal model that closely recapitulates all aspects of the human disease [1]. To fill this gap, many mouse phenotypes have been developed, each with strengths and weaknesses relative to human psoriasis [2]. IMQ dermatitis has been widely investigated as a psoriasiform phenotype, in part due to its macroscopic appearance, histologic features, and partial dependence upon pathways known to mediate development of the human disease [3]. Similar to other mouse psoriasiform phenotypes [2], this study shows that the IMQ dermatitis expression signature exhibits strong resemblance to human psoriasis (0.05 ≤ *r*_s_ ≤ 0.45; Additional file 8), with mouse–human correlation estimates better than those reported for other types of acute inflammatory response [75]. This correspondence, however, varies among strains and was strongest in B6 mice (Fig. 8d; Additional file 8a; Additional file 13b, e). It is also important to recognize that, regardless of strain, IMQ-driven expression shifts do not uniquely match psoriasis, but are consistent with several human skin conditions (e.g., eschars, acne, infection, wounding; Fig. 8). The basis for this correspondence is activation of core pathways related to positive regulation of immune response, neutrophil chemotaxis, leukocyte migration, and bacterial defense response (Figs. 3f and 8c). Along with this, decreased expression of homeostatic genes is a common effect of IMQ across strains that is also observed in psoriasis and other skin conditions (Fig. 3g; Additional file 9a). To some degree, therefore, IMQ may trigger innate immune responses and breakdown of epidermal homeostasis intrinsic to several forms of cutaneous pathology [45].

Psoriasis is a complex genetic disease with at least 41 genetic susceptibility loci contributing to the pathogenesis [76], and there continues to be progress towards deciphering the genetics of other skin diseases as well [77]. Considering the additional influence of environmental factors [78], it is tempting to argue that studies focused exclusively on genetically homogenous inbred mice, despite all their advantages, can never succeed in fully replicating human diseases and their complexity [79]. An advantage of IMQ, however, is its flexibility and convenience as an experimental approach, which in this study allowed us to demonstrate strain- and sex-dependent effects. A study of similar scale using any other proposed model of psoriasisiform dermatitis would have been difficult or impossible. This flexibility and ease-of-use remains the key advantage of the IMQ model and, in principle, could allow for more robust mechanistic studies that routinely consider the roles of sex and genetic background. Evaluation of multiple strains may lead to conflicting results, possibly challenging observations established from focused studies of individual strains [10, 11]. This approach, however, should offer dividends through more robust conclusions and stronger reproducibility of experimental results [13], ultimately improving the odds that mechanistic findings are successfully translated from mice to inform our understanding of disease progression in human patients [10].

## Conclusions

The characteristics of IMQ dermatitis have been extensively investigated in prior mouse studies, with the expectation that such work carries implications for human psoriasis [3–6]. Mechanisms established by these studies, however, may be influenced by genetic background, complicating inferences with regard to human psoriasis. This study showed that key features of the cutaneous phenotype generated by Aldara (5% IMQ) are influenced by strain and sex, including features related to central aspects of psoriasis lesion development, such as epidermal proliferation, IL-17A signaling, and T lymphocyte activity. This study also shows that gene expression patterns associated with IMQ dermatitis, while bearing significant resemblance to human psoriasis, are also aligned closely with expression signatures of other human skin diseases (e.g., wounding, acne, eschars, and infection). For some strains, expression responses to IMQ were most consistent with expression shifts observed in skin wounds or infections (BALB/c, 129/SvJ, DBA, MOLF). Nonetheless, IMQ-induced expression shifts on the B6 background were more consistent with psoriasis than any other disease included in our analyses, and overall, the B6 phenotype matched psoriasis better than other strain phenotypes. B6 mice may therefore provide a better genetic background than other strains for using IMQ to model psoriasis in the laboratory mouse.

## Additional files

Additional file 1: Histograms of weight change, epidermal thickness, and spleen weight. (PDF 403 kb)
Additional file 2: Mapping of reads to mouse genome (*n* = 56 samples). (PDF 723 kb)
Additional file 3: CTL sample expression among mouse strains and comparison to normal human skin. (PDF 1321 kb)
Additional file 4: RNA-seq exploratory analyses. (PDF 975 kb)
Additional file 5: Comparison of IMQ responses between males and females. (PDF 1350 kb)
Additional file 6: Number of differentially expressed genes and associations with CTL body weight, epidermal thickness, and spleen weight. (PDF 286 kb)
Additional file 7: Microarray dataset comparison. (PDF 1604 kb)
Additional file 8: Comparison of IMQ responses to human psoriasis lesions. (PDF 1593 kb)
Additional file 9: Gene Ontology (GO) biological process (BP) terms enriched among psoriasis-decreased DEGs and correspondence with IMQ responses. (PDF 1635 kb)
Additional file 10: IL-17 family gene expression (*Il17a*, *Il17b*, *Il17c*, *Il17d*, *Il17e*, *Il17f*). (PDF 506 kb)
Additional file 11: Genes uniquely altered by IMQ in MOLF males. (PDF 1511 kb)
Additional file 12: Genes associated with Toll-like receptor (TLR) signaling are disproportionately elevated by IMQ in all strains. (PDF 1173 kb)
Additional file 13: Psoriasis-specific genes with altered expression in psoriasis lesions but not other human skin diseases. (PDF 1382 kb)

## Abbreviations

B6: C57BL/6 J
BP: Biological process
CTL: Control
DC: Dendritic cell
DEG: Differentially expressed gene
FDR: False discovery rate
FPKM: Fragments per kilobase of transcript per million mapped reads
GEO: Gene Expression Omnibus
GO: Gene Ontology
IFN: Interferon
IGP: Immunological Genome Project
IL: Interleukin
IMQ: Imiquimod
KC: Keratinocyte
MSF: mediterranean spotted fever
pDC: Plasmacytoid dendritic cell
TLR: Toll-like receptor
TNF: Tumor necrosis factor.

## References

[1] Gudjonsson JE, Johnston A, Dyson M, Valdimarsson H, Elder JT (2007). Mouse models of psoriasis. J Invest Dermatol.

[2] Swindell WR, Johnston A, Carbajal S, Han G, Wohn C, Lu J, Xing X, Nair RP, Voorhees JJ, Elder JT (2011). Genome-wide expression profiling of five mouse models identifies similarities and differences with human psoriasis. PLoS One.

[3] van der Fits L, Mourits S, Voerman JS, Kant M, Boon L, Laman JD, Cornelissen F, Mus AM, Florencia E, Prens EP, Lubberts E (2009). Imiquimod-induced psoriasis-like skin inflammation in mice is mediated via the IL-23/IL-17 axis. J Immunol.

[4] Flutter B, Nestle FO (2013). TLRs to cytokines: mechanistic insights from the imiquimod mouse model of psoriasis. Eur J Immunol.

[5] Pantelyushin S, Haak S, Ingold B, Kulig P, Heppner FL, Navarini AA, Becher B (2012). Rorgammat + innate lymphocytes and gammadelta T cells initiate psoriasiform plaque formation in mice. J Clin Invest.

[6] Van Belle AB, de Heusch M, Lemaire MM, Hendrickx E, Warnier G, Dunussi-Joannopoulos K, Fouser LA, Renauld JC, Dumoutier L (2012). IL-22 is required for imiquimod-induced psoriasiform skin inflammation in mice. J Immunol.

[7] Elder JT, Bruce AT, Gudjonsson JE, Johnston A, Stuart PE, Tejasvi T, Voorhees JJ, Abecasis GR, Nair RP (2010). Molecular dissection of psoriasis: integrating genetics and biology. J Invest Dermatol.

[8] Kong BY, Haugh IM, Schlosser BJ, Getsios S, Paller AS (2016). Mind the gap: sex bias in basic skin research. J Invest Dermatol.

[9] Bezdek S, Hdnah A, Sezin T, Mousavi S, Zillikens D, Ibrahim S, Ludwig RJ, Sadik CD. The genetic difference between C57Bl/6J and C57Bl/6N mice significantly impacts Aldara-induced psoriasiform dermatitis. Exp Dermatol. 2016. doi:10.1111/exd.13131. 10.1111/exd.13131 27315297

[10] Churchill GA (2014). Misleading results: don’t blame the mice. Science.

[11] Iwata H, Witte M, Samavedam UK, Gupta Y, Shimizu A, Ishiko A, Schroder T, Seeger K, Dahlke M, Rades D (2015). Radiosensitive hematopoietic cells determine the extent of skin inflammation in experimental epidermolysis bullosa acquisita. J Immunol.

[12] Prinz F, Schlange T, Asadullah K (2011). Believe it or not: how much can we rely on published data on potential drug targets?. Nat Rev Drug Discov.

[13] Collins FS, Tabak LA (2014). Policy: NIH plans to enhance reproducibility. Nature.

[14] Sarup P, Sorensen JG, Kristensen TN, Hoffmann AA, Loeschcke V, Paige KN, Sorensen P (2011). Candidate genes detected in transcriptome studies are strongly dependent on genetic background. PLoS One.

[15] Burnett C, Valentini S, Cabreiro F, Goss M, Somogyvari M, Piper MD, Hoddinott M, Sutphin GL, Leko V, McElwee JJ (2011). Absence of effects of Sir2 overexpression on lifespan in C. elegans and Drosophila. Nature.

[16] Liao CY, Rikke BA, Johnson TE, Diaz V, Nelson JF (2010). Genetic variation in the murine lifespan response to dietary restriction: from life extension to life shortening. Aging Cell.

[17] Chandler CH, Chari S, Dworkin I (2013). Does your gene need a background check? How genetic background impacts the analysis of mutations, genes, and evolution. Trends Genet.

[18] Stephan K, Smirnova I, Jacque B, Poltorak A (2007). Genetic analysis of the innate immune responses in wild-derived inbred strains of mice. Eur J Immunol.

[19] Gudjonsson JSW, Michaels K, Sutter A, Diaconu D, Fritz Y, Xing X, Sarkar M, Liang Y, Tsoi A, Ward N (2016). Imiquimod has strain-dependent effects in mice and does not uniquely model human psoriasis. Exp Dermatol.

[20] Di Meglio P, Duarte JH, Ahlfors H, Owens ND, Li Y, Villanova F, Tosi I, Hirota K, Nestle FO, Mrowietz U (2014). Activation of the aryl hydrocarbon receptor dampens the severity of inflammatory skin conditions. Immunity.

[21] Bai J, Liu Z, Xu Z, Ke F, Zhang L, Zhu H, Lou F, Wang H, Fei Y, Shi YL, Wang H (2015). Epigenetic downregulation of SFRP4 contributes to epidermal hyperplasia in psoriasis. J Immunol.

[22] Kjaer TN, Thorsen K, Jessen N, Stenderup K, Pedersen SB (2015). Resveratrol ameliorates imiquimod-induced psoriasis-like skin inflammation in mice. PLoS One.

[23] Petkov PM, Ding Y, Cassell MA, Zhang W, Wagner G, Sargent EE, Asquith S, Crew V, Johnson KA, Robinson P (2004). An efficient SNP system for mouse genome scanning and elucidating strain relationships. Genome Res.

[24] Chia R, Achilli F, Festing MF, Fisher EM (2005). The origins and uses of mouse outbred stocks. Nat Genet.

[25] Shah PP, Desai PR, Patel AR, Singh MS (2012). Skin permeating nanogel for the cutaneous co-delivery of two anti-inflammatory drugs. Biomaterials.

[26] Ando N, Nakamura Y, Aoki R, Ishimaru K, Ogawa H, Okumura K, Shibata S, Shimada S, Nakao A (2015). Circadian gene clock regulates psoriasis-like skin inflammation in mice. J Invest Dermatol.

[27] Wolfram JA, Diaconu D, Hatala DA, Rastegar J, Knutsen DA, Lowther A, Askew D, Gilliam AC, McCormick TS, Ward NL (2009). Keratinocyte but not endothelial cell-specific overexpression of Tie2 leads to the development of psoriasis. Am J Pathol.

[28] Ward NL, Hatala DA, Wolfram JA, Knutsen DA, Loyd CM (2011). Cutaneous manipulation of vascular growth factors leads to alterations in immunocytes, blood vessels and nerves: Evidence for a cutaneous neurovascular unit. J Dermatol Sci.

[29] Martin M (2011). Cutadapt removes adapter sequences from high-throughput sequencing reads. EMBnetjournal.

[30] Del Fabbro C, Scalabrin S, Morgante M, Giorgi FM (2013). An extensive evaluation of read trimming effects on Illumina NGS data analysis. PLoS One.

[31] FASTX-Toolkit. http://hannonlab.cshl.edu/fastx_toolkit/. Accessed 8 Mar 2017.

[32] FastQC: a quality control tool for high throughput sequence data. http://www.bioinformatics.babraham.ac.uk/projects/fastqc/. Accessed 8 Mar 2017.

[33] Kim D, Pertea G, Trapnell C, Pimentel H, Kelley R, Salzberg SL (2013). TopHat2: accurate alignment of transcriptomes in the presence of insertions, deletions and gene fusions. Genome Biol.

[34] Speir ML, Zweig AS, Rosenbloom KR, Raney BJ, Paten B, Nejad P, Lee BT, Learned K, Karolchik D, Hinrichs AS (2016). The UCSC Genome Browser database: 2016 update. Nucleic Acids Res.

[35] Li H, Handsaker B, Wysoker A, Fennell T, Ruan J, Homer N, Marth G, Abecasis G, Durbin R (2009). The Sequence Alignment/Map format and SAMtools. Bioinformatics.

[36] Anders S, Pyl PT, Huber W (2015). HTSeq-a Python framework to work with high-throughput sequencing data. Bioinformatics.

[37] Trapnell C, Roberts A, Goff L, Pertea G, Kim D, Kelley DR, Pimentel H, Salzberg SL, Rinn JL, Pachter L (2012). Differential gene and transcript expression analysis of RNA-seq experiments with TopHat and Cufflinks. Nat Protoc.

[38] Wang L, Wang S, Li W (2012). RSeQC: quality control of RNA-seq experiments. Bioinformatics.

[39] DeLuca DS, Levin JZ, Sivachenko A, Fennell T, Nazaire MD, Williams C, Reich M, Winckler W, Getz G (2012). RNA-SeQC: RNA-seq metrics for quality control and process optimization. Bioinformatics.

[40] Robinson MD, McCarthy DJ, Smyth GK (2010). edgeR: a Bioconductor package for differential expression analysis of digital gene expression data. Bioinformatics.

[41] Robinson MD, Oshlack A (2010). A scaling normalization method for differential expression analysis of RNA-seq data. Genome Biol.

[42] Landau WM, Liu P (2013). Dispersion estimation and its effect on test performance in RNA-seq data analysis: a simulation-based comparison of methods. PLoS One.

[43] Benjamini Y, Hochberg Y (1995). Controlling the false discovery rate: a powerful and practical approach to multiple testing. J R Stat Soc B.

[44] Bult CJ, Eppig JT, Blake JA, Kadin JA, Richardson JE (2016). Mouse genome database 2016. Nucleic Acids Res.

[45] Swindell WR, Sarkar MK, Liang Y, Xing X, Gudjonsson JE. Cross-disease transcriptomics: Unique IL-17A signaling in psoriasis lesions and an autoimmune PBMC signature. J Invest Dermatol. 2016;136:1820–30. 10.1016/j.jid.2016.04.035 PMC5234565 27206706

[46] Karim R, Meyers C, Backendorf C, Ludigs K, Offringa R, van Ommen GJ, Melief CJ, van der Burg SH, Boer JM (2011). Human papillomavirus deregulates the response of a cellular network comprising of chemotactic and proinflammatory genes. PLoS One.

[47] Swindell WR, Xing X, Fritz Y, Diaconu D, Simon DI, Ward NL, Gudjonsson JE. Deficiency of myeloid-related proteins 8 and 14 (Mrp8/Mrp14) does not block inflammaging but prevents steatosis. Oncotarget. 2016;7:35535–51. 10.18632/oncotarget.9550 PMC5094943 27224926

[48] Kim CC, Lanier LL (2013). Beyond the transcriptome: completion of act one of the Immunological Genome Project. Curr Opin Immunol.

[49] Gulati N, Krueger JG, Suarez-Farinas M, Mitsui H (2013). Creation of differentiation-specific genomic maps of human epidermis through laser capture microdissection. J Invest Dermatol.

[50] Lopez-Pajares V, Qu K, Zhang J, Webster DE, Barajas BC, Siprashvili Z, Zarnegar BJ, Boxer LD, Rios EJ, Tao S (2015). A LncRNA-MAF:MAFB transcription factor network regulates epidermal differentiation. Dev Cell.

[51] Greco JA, Pollins AC, Boone BE, Levy SE, Nanney LB (2010). A microarray analysis of temporal gene expression profiles in thermally injured human skin. Burns.

[52] Novais FO, Carvalho LP, Passos S, Roos DS, Carvalho EM, Scott P, Beiting DP (2015). Genomic profiling of human Leishmania braziliensis lesions identifies transcriptional modules associated with cutaneous immunopathology. J Invest Dermatol.

[53] Irizarry RA, Hobbs B, Collin F, Beazer-Barclay YD, Antonellis KJ, Scherf U, Speed TP (2003). Exploration, normalization, and summaries of high density oligonucleotide array probe level data. Biostatistics.

[54] Smyth GK (2004). Linear models and empirical bayes methods for assessing differential expression in microarray experiments. Stat Appl Genet Mol Biol.

[55] Livak KJ, Schmittgen TD (2001). Analysis of relative gene expression data using real-time quantitative PCR and the 2(-Delta Delta C(T)) method. Methods.

[56] Conner JR, Smirnova II, Poltorak A (2008). Forward genetic analysis of Toll-like receptor responses in wild-derived mice reveals a novel antiinflammatory role for IRAK1BP1. J Exp Med.

[57] Sebastiani G, Leveque G, Lariviere L, Laroche L, Skamene E, Gros P, Malo D (2000). Cloning and characterization of the murine toll-like receptor 5 (Tlr5) gene: sequence and mRNA expression studies in Salmonella-susceptible MOLF/Ei mice. Genomics.

[58] Kanehisa M, Sato Y, Kawashima M, Furumichi M, Tanabe M (2016). KEGG as a reference resource for gene and protein annotation. Nucleic Acids Res.

[59] Clayton JA (2016). Studying both sexes: a guiding principle for biomedicine. Faseb J.

[60] Sofen H, Smith S, Matheson RT, Leonardi CL, Calderon C, Brodmerkel C, Li K, Campbell K, Marciniak SJ, Wasfi Y (2014). Guselkumab (an IL-23-specific mAb) demonstrates clinical and molecular response in patients with moderate-to-severe psoriasis. J Allergy Clin Immunol.

[61] Papp KA, Leonardi C, Menter A, Ortonne JP, Krueger JG, Kricorian G, Aras G, Li J, Russell CB, Thompson EH, Baumgartner S (2012). Brodalumab, an anti-interleukin-17-receptor antibody for psoriasis. N Engl J Med.

[62] Leonardi C, Matheson R, Zachariae C, Cameron G, Li L, Edson-Heredia E, Braun D, Banerjee S (2012). Anti-interleukin-17 monoclonal antibody ixekizumab in chronic plaque psoriasis. N Engl J Med.

[63] Tortola L, Rosenwald E, Abel B, Blumberg H, Schafer M, Coyle AJ, Renauld JC, Werner S, Kisielow J, Kopf M (2012). Psoriasiform dermatitis is driven by IL-36-mediated DC-keratinocyte crosstalk. J Clin Invest.

[64] Ramirez-Carrozzi V, Sambandam A, Luis E, Lin Z, Jeet S, Lesch J, Hackney J, Kim J, Zhou M, Lai J (2011). IL-17C regulates the innate immune function of epithelial cells in an autocrine manner. Nat Immunol.

[65] Wohn C, Ober-Blobaum JL, Haak S, Pantelyushin S, Cheong C, Zahner SP, Onderwater S, Kant M, Weighardt H, Holzmann B (2013). Langerin(neg) conventional dendritic cells produce IL-23 to drive psoriatic plaque formation in mice. Proc Natl Acad Sci U S A.

[66] Asselin-Paturel C, Brizard G, Pin JJ, Briere F, Trinchieri G (2003). Mouse strain differences in plasmacytoid dendritic cell frequency and function revealed by a novel monoclonal antibody. J Immunol.

[67] Nakano H, Yanagita M, Gunn MD (2001). CD11c(+)B220(+)Gr-1(+) cells in mouse lymph nodes and spleen display characteristics of plasmacytoid dendritic cells. J Exp Med.

[68] Grine L, Dejager L, Libert C, Vandenbroucke RE (2015). Dual inhibition of TNFR1 and IFNAR1 in imiquimod-induced psoriasiform skin inflammation in mice. J Immunol.

[69] Guenet JL, Bonhomme F (2003). Wild mice: an ever-increasing contribution to a popular mammalian model. Trends Genet.

[70] Smith PM, Jacque B, Conner JR, Poltorak A, Stadecker MJ (2011). IRAK-2 regulates IL-1-mediated pathogenic Th17 cell development in helminthic infection. PLoS Pathog.

[71] Conner JR, Smirnova II, Poltorak A (2009). A mutation in Irak2c identifies IRAK-2 as a central component of the TLR regulatory network of wild-derived mice. J Exp Med.

[72] Schworer SA, Smirnova II, Kurbatova I, Bagina U, Churova M, Fowler T, Roy AL, Degterev A, Poltorak A (2014). Toll-like receptor-mediated down-regulation of the deubiquitinase cylindromatosis (CYLD) protects macrophages from necroptosis in wild-derived mice. J Biol Chem.

[73] Yin C, Zhang T, Qiao L, Du J, Li S, Zhao H, Wang F, Huang Q, Meng W, Zhu H (2014). TLR7-expressing cells comprise an interfollicular epidermal stem cell population in murine epidermis. Sci Rep.

[74] Walter A, Schafer M, Cecconi V, Matter C, Urosevic-Maiwald M, Belloni B, Schonewolf N, Dummer R, Bloch W, Werner S (2013). Aldara activates TLR7-independent immune defence. Nat Commun.

[75] Seok J, Warren HS, Cuenca AG, Mindrinos MN, Baker HV, Xu W, Richards DR, McDonald-Smith GP, Gao H, Hennessy L (2013). Genomic responses in mouse models poorly mimic human inflammatory diseases. Proc Natl Acad Sci U S A.

[76] Tsoi LC, Spain SL, Ellinghaus E, Stuart PE, Capon F, Knight J, Tejasvi T, Kang HM, Allen MH, Lambert S (2015). Enhanced meta-analysis and replication studies identify five new psoriasis susceptibility loci. Nat Commun.

[77] DeStefano GM, Christiano AM. The genetics of human skin disease. Cold Spring Harb Perspect Med. 2014;4:a015172. 10.1101/cshperspect.a015172 PMC4200211 25274756

[78] Dika E, Bardazzi F, Balestri R, Maibach HI (2007). Environmental factors and psoriasis. Curr Probl Dermatol.

[79] Mak IW, Evaniew N, Ghert M (2014). Lost in translation: animal models and clinical trials in cancer treatment. Am J Transl Res.

