# Supplementary material for: Imiquimod has strain-dependent effects in mice and does not uniquely model human psoriasis
Source: Genome Med. 2017 Mar 9;9:24. doi: 10.1186/s13073-017-0415-3 (PMC5345243; doi:10.1186/s13073-017-0415-3)
Supplement: Additional file 1: — Histograms of weight change, epidermal thickness, and spleen weight. (PDF 403 kb) [file 13073_2017_415_MOESM1_ESM.pdf]

Additional File 1. Histograms of weight change, epidermal thickness and spleen weight.

(A, D) Distribution of weight change in CTL (n = 70) and IMQ (n = 70) mice. Negative values indicate weight loss with CTL or IMQ treatment. (B, E) Distribution of $\log_{10}$(epidermal thickness) in CTL (n = 69) and IMQ (n = 70) mice. (C, F) Distribution of spleen weight in CTL (n = 69) and IMQ (n = 69) mice. Dotted red lines in (A) – (F) represent density estimates obtained using the Gaussian kernel.

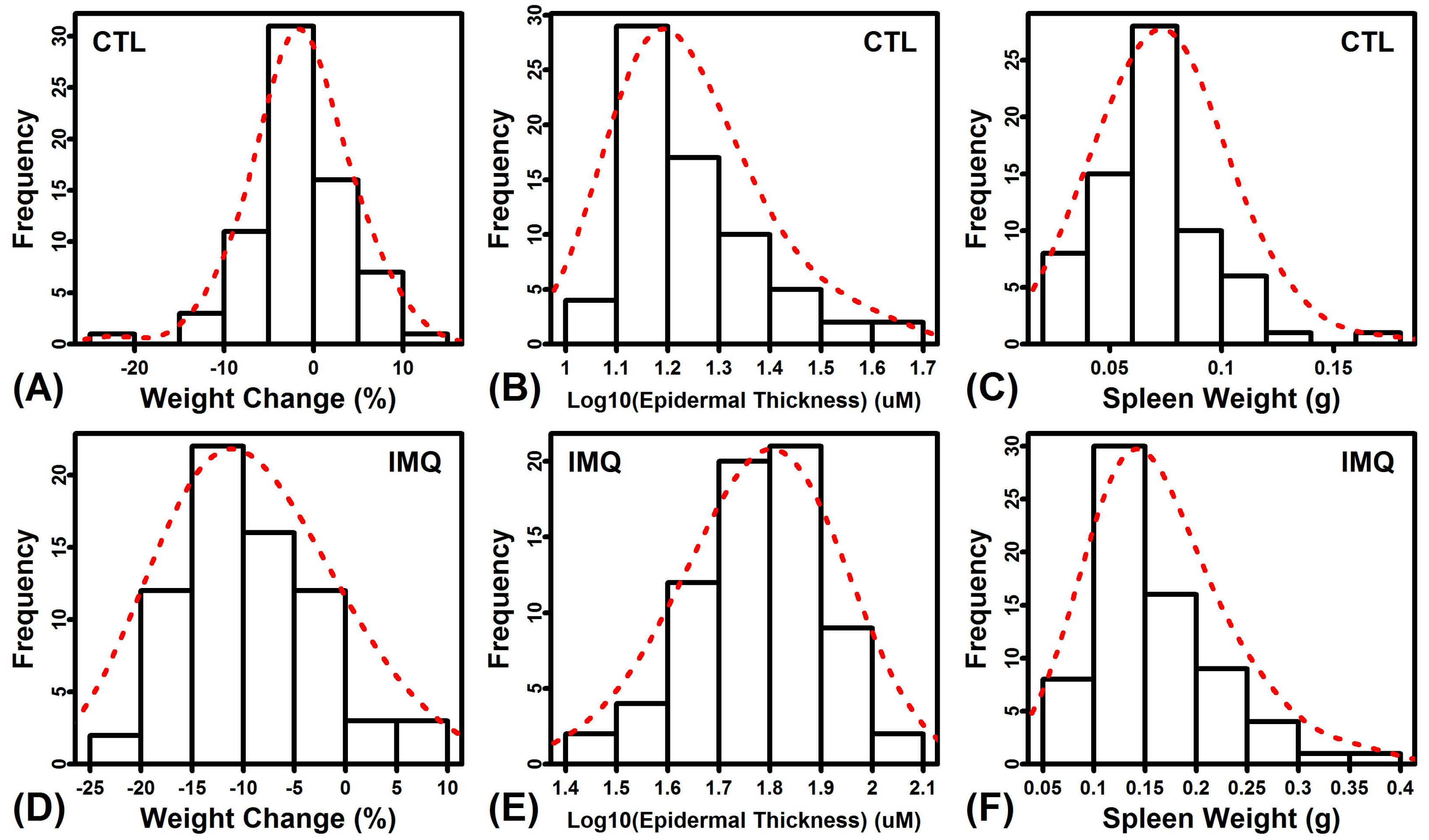
CTL
Frequency
(A)
Weight Change (%)
CTL
Frequency
(B)
Log10(Epidermal Thickness) (uM)
CTL
Frequency
(C)
Spleen Weight (g)
IMQ
Frequency
(D)
Weight Change (%)
IMQ
Frequency
(E)
Log10(Epidermal Thickness) (uM)
IMQ
Frequency
(F)
Spleen Weight (g)
