# Supplementary material for: Imiquimod has strain-dependent effects in mice and does not uniquely model human psoriasis
Source: Genome Med. 2017 Mar 9;9:24. doi: 10.1186/s13073-017-0415-3 (PMC5345243; doi:10.1186/s13073-017-0415-3)
Supplement: Additional file 2: — Mapping of reads to mouse genome (n = 56 samples). (PDF 723 kb) [file 13073_2017_415_MOESM2_ESM.pdf]

Additional File 2. Mapping of reads to mouse genome (*n* = 56 samples). (A) Reads per sample. (B) Quality-filtered reads per sample. (C) Percentage of quality-filtered reads mapped to the mouse genome (GRCm38/mm10). (D) Average percentage of mapped quality-filtered reads per strain. (E) Percentage of reads mapped to exonic regions. (F) Average percentage of exonic reads per strain. In (D) and (F), groups without the same letter differ significantly ($P < 0.05$, Tukey honest significant difference).

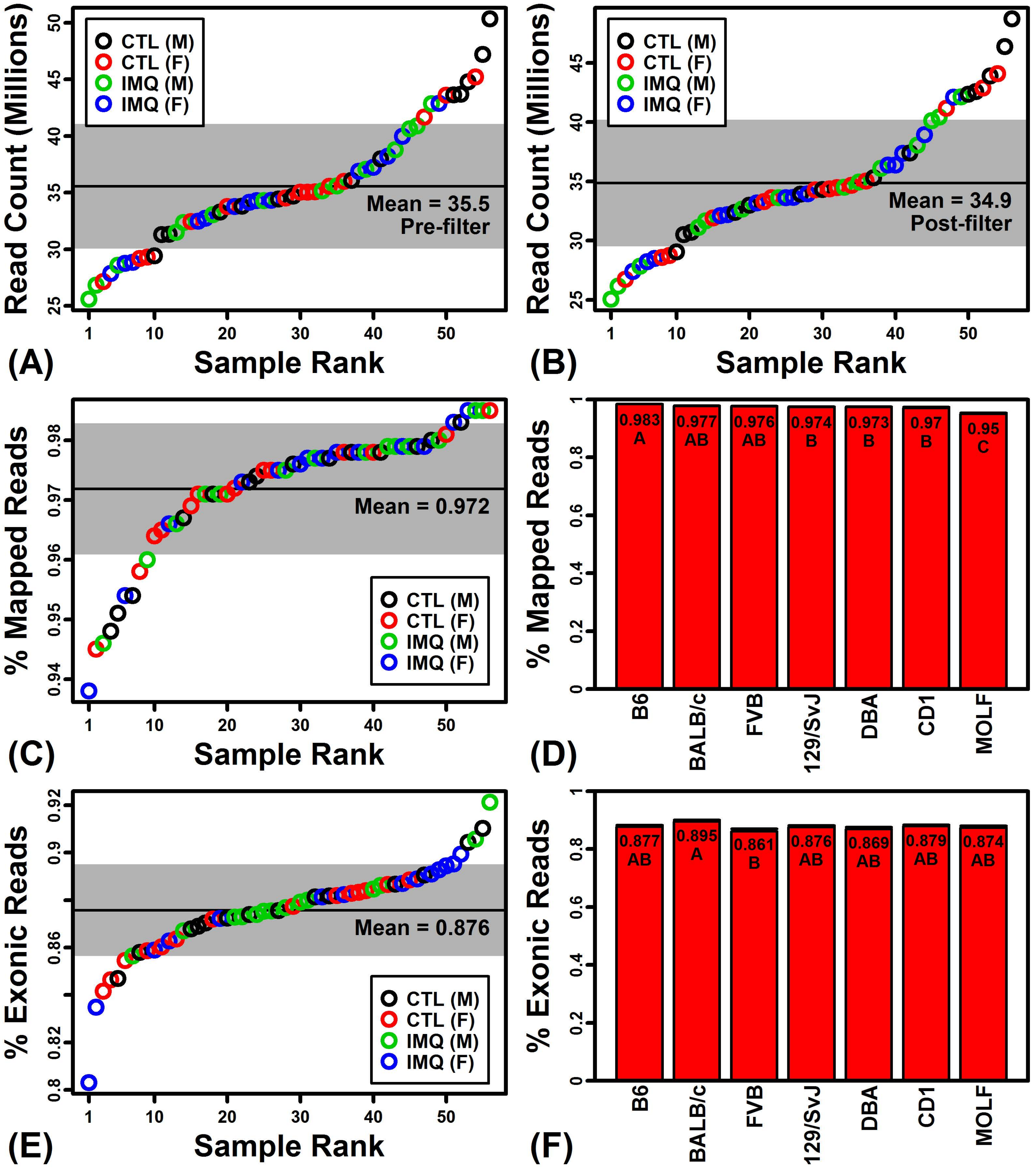

Read Count (Millions)
CTL (M)
CTL (F)
IMQ (M)
IMQ (F)
Mean = 35.5
Pre-filter
Sample Rank
(A)
Read Count (Millions)
Mean = 34.9
Post-filter
Sample Rank
(B)
% Mapped Reads
Mean = 0.972
Sample Rank
(C)
% Mapped Reads
0.983 A
0.977 AB
0.976 AB
0.974 B
0.973 B
0.97 B
0.95 C
B6
BALB/c
FVB
129/SvJ
DBA
CD1
MOLF
(D)
% Exonic Reads
Mean = 0.876
Sample Rank
(E)
% Exonic Reads
0.877 AB
0.895 A
0.861 B
0.876 AB
0.869 AB
0.879 AB
0.874 AB
(F)
