# Supplementary material for: Imiquimod has strain-dependent effects in mice and does not uniquely model human psoriasis
Source: Genome Med. 2017 Mar 9;9:24. doi: 10.1186/s13073-017-0415-3 (PMC5345243; doi:10.1186/s13073-017-0415-3)
Supplement: Additional file 3: — CTL sample expression among mouse strains and comparison to normal human skin. (PDF 1321 kb) [file 13073_2017_415_MOESM3_ESM.pdf]

Additional File 3. CTL sample expression among mouse strains and comparison to normal human skin. (A – G) Spearman correlation of average expression (FPKM) in CTL mouse skin (*n* = 4 per strain, males + females) and normal human skin (*n* = 90 subjects; GSE54456). Dashed lines represent robust linear regression estimates. (H) Overlap of robust regression estimates for each strain. (I) Genes with highest expression in normal human skin (bottom margin parentheses: average FPKM). Heatmap colors depict average FPKM in CTL mouse skin for each strain (see legend). Relative FPKM across strains is indicated by values within the heatmap (1 = strain with highest expression; left margin parentheses: average relative expression across genes). (J) Number of genes differentially expressed between CTL samples (FDR < 0.10; FC > 2.0 or FC < 0.50; all 21 possible strain comparisons; red: higher expression in first strain listed; blue: higher expression in second strain listed). (K – N) Genes with different expression in CTL samples from B6 and MOLF mice. Groups without the same letter differ significantly (P < 0.05, Tukey honest significant difference; Error bars: standard error of the mean).

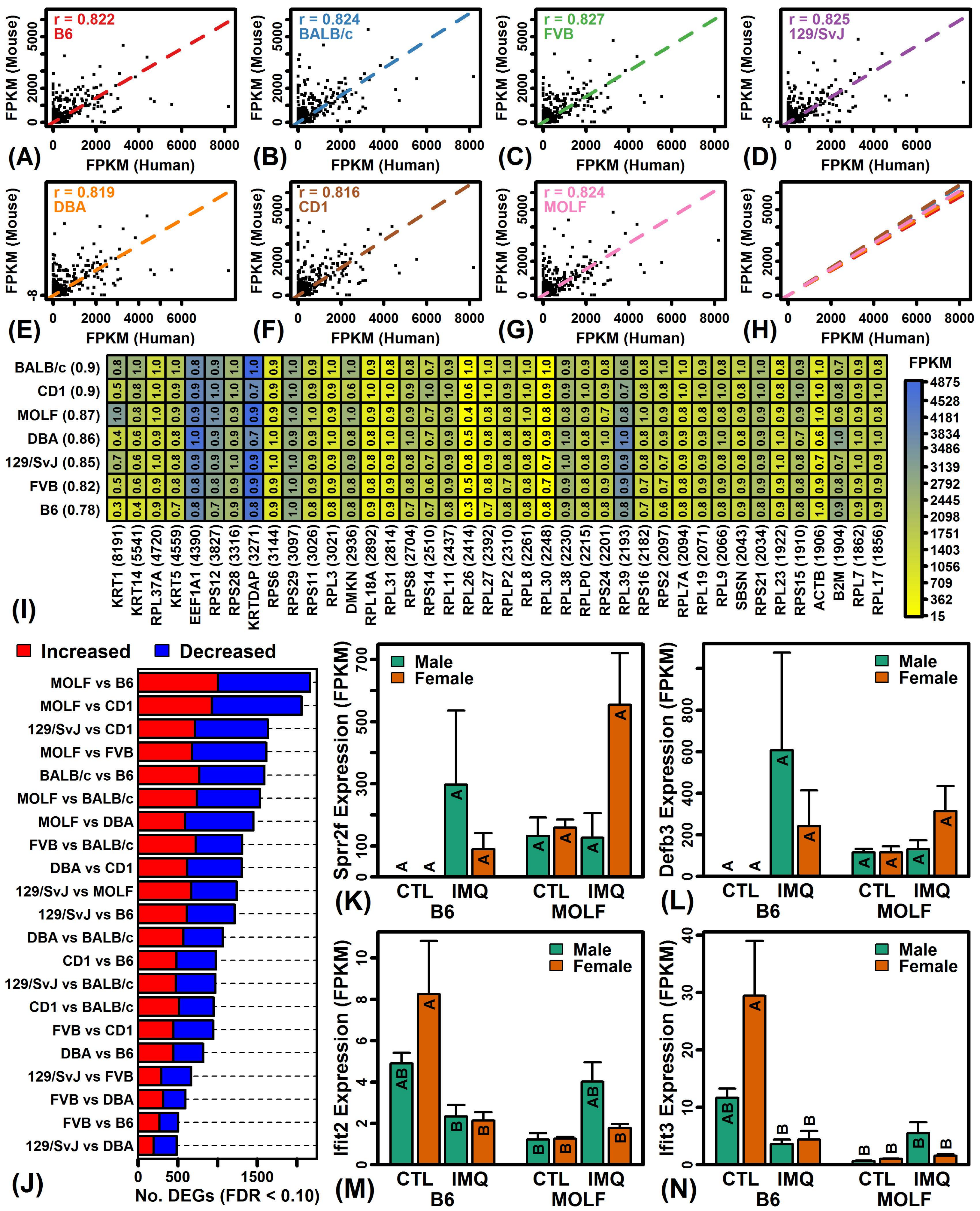

r = 0.822
B6
r = 0.824
BALB/c
r = 0.827
FVB
r = 0.825
129/SvJ
r = 0.819
DBA
r = 0.816
CD1
r = 0.824
MOLF
FPKM (Mouse)
FPKM (Human)
(A)
(B)
(C)
(D)
(E)
(F)
(G)
(H)
(I)
(J)
(K)
(L)
(M)
(N)
BALB/c (0.9)
CD1 (0.9)
MOLF (0.87)
DBA (0.86)
129/SvJ (0.85)
FVB (0.82)
B6 (0.78)
KRT1 (8191)
KRT14 (5541)
RPL37A (4720)
KRT5 (4559)
EEF1A1 (4390)
RPS12 (3827)
RPS28 (3316)
KRTDAP (3271)
RPS6 (3144)
RPS29 (3097)
RPS11 (3026)
RPL3 (3021)
DMKN (2936)
RPL18A (2892)
RPL31 (2814)
RPS8 (2704)
RPS14 (2510)
RPL11 (2437)
RPL26 (2414)
RPL27 (2392)
RPLP2 (2310)
RPL8 (2261)
RPL30 (2248)
RPL38 (2230)
RPLP0 (2215)
RPS24 (2201)
RPL39 (2193)
RPS16 (2182)
RPS2 (2097)
RPL7A (2094)
RPL19 (2071)
RPL9 (2066)
SBSN (2043)
RPS21 (2034)
RPL23 (1922)
RPS15 (1910)
ACTB (1906)
B2M (1904)
RPL7 (1862)
RPL17 (1856)
FPKM
Increased
Decreased
MOLF vs B6
MOLF vs CD1
129/SvJ vs CD1
MOLF vs FVB
BALB/c vs B6
MOLF vs BALB/c
MOLF vs DBA
FVB vs BALB/c
DBA vs CD1
129/SvJ vs MOLF
129/SvJ vs B6
DBA vs BALB/c
CD1 vs B6
129/SvJ vs BALB/c
CD1 vs BALB/c
FVB vs CD1
DBA vs B6
129/SvJ vs FVB
FVB vs DBA
FVB vs B6
129/SvJ vs DBA
No. DEGs (FDR < 0.10)
Male
Female
Sprr2f Expression (FPKM)
Defb3 Expression (FPKM)
Ifit2 Expression (FPKM)
Ifit3 Expression (FPKM)
CTL
IMQ
B6
MOLF
