# Supplementary material for: Imiquimod has strain-dependent effects in mice and does not uniquely model human psoriasis
Source: Genome Med. 2017 Mar 9;9:24. doi: 10.1186/s13073-017-0415-3 (PMC5345243; doi:10.1186/s13073-017-0415-3)
Supplement: Additional file 4: — RNA-seq exploratory analyses. (PDF 975 kb) [file 13073_2017_415_MOESM4_ESM.pdf]

Additional File 4. RNA-seq exploratory analyses. (A) Cluster analysis. Protein-coding gene expression profiles were averaged across replicates and clustered (average linkage and Euclidean distance). (B, C) Spearman correlations between expression profiles in (B) males and (C) females. (D) The 28 sex-strain-treatment combinations were plotted with respect to the first two principal components. (E) Variable importance. Linear models were generated for each gene with strain, treatment and sex as explanatory factors. Importance of each factor was assessed using likelihood ratio tests (LRTs). Boxes span the middle 50% of $-\log_{10}$-transformed p-values from LRTs for each factor (whiskers: 10th and 90th percentiles).

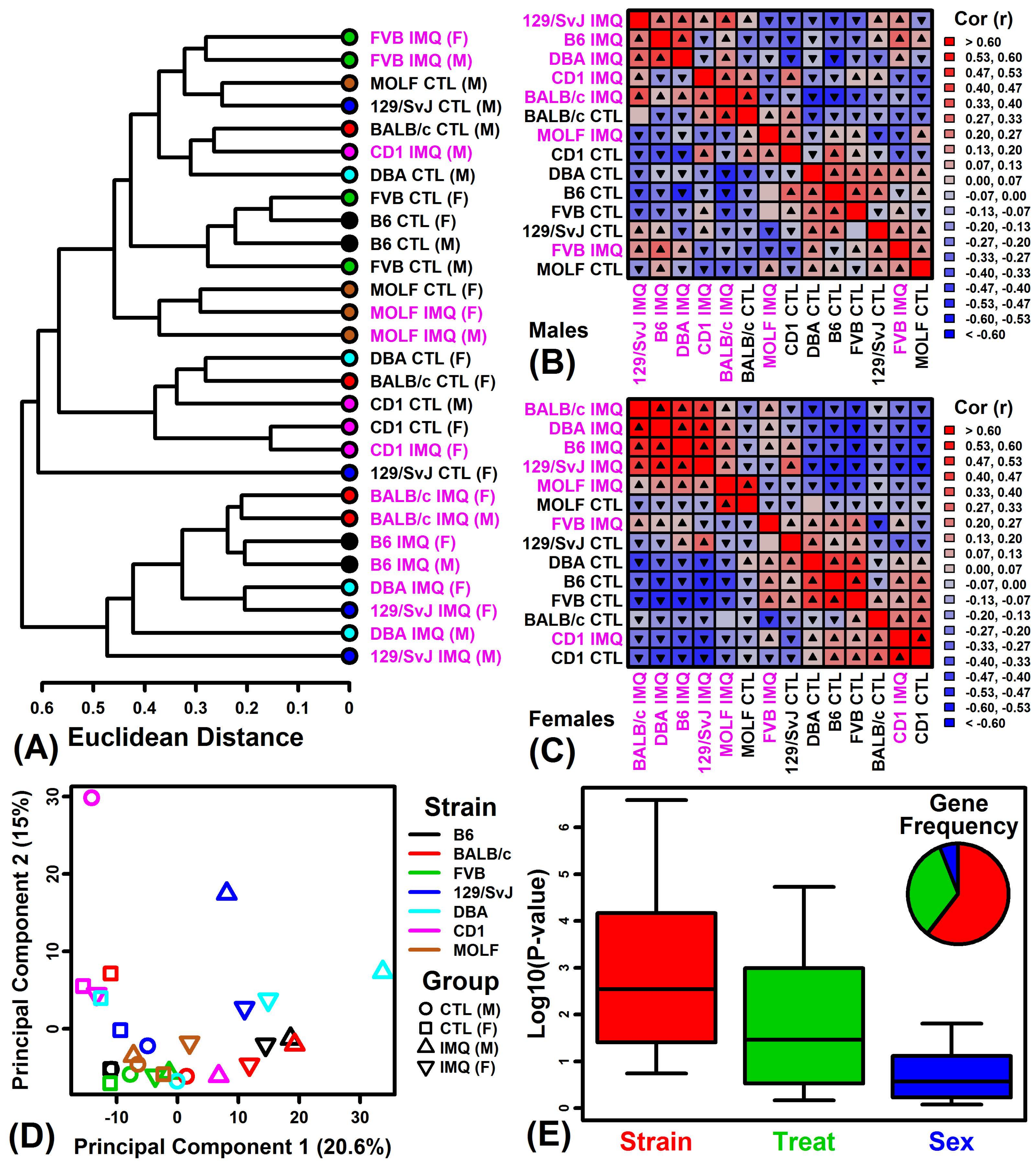

FVB IMQ (F)
FVB IMQ (M)
MOLF CTL (M)
129/SvJ CTL (M)
BALB/c CTL (M)
CD1 IMQ (M)
DBA CTL (M)
FVB CTL (F)
B6 CTL (F)
B6 CTL (M)
FVB CTL (M)
MOLF CTL (F)
MOLF IMQ (F)
MOLF IMQ (M)
DBA CTL (F)
BALB/c CTL (F)
CD1 CTL (M)
CD1 CTL (F)
CD1 IMQ (F)
129/SvJ CTL (F)
BALB/c IMQ (F)
BALB/c IMQ (M)
B6 IMQ (F)
B6 IMQ (M)
DBA IMQ (F)
129/SvJ IMQ (F)
DBA IMQ (M)
129/SvJ IMQ (M)
0.6
0.5
0.4
0.3
0.2
0.1
0
(A)
Euclidean Distance
129/SvJ IMQ
B6 IMQ
DBA IMQ
CD1 IMQ
BALB/c IMQ
BALB/c CTL
MOLF IMQ
CD1 CTL
DBA CTL
B6 CTL
FVB CTL
129/SvJ CTL
FVB IMQ
MOLF CTL
Males
(B)
Cor (r)
> 0.60
0.53, 0.60
0.47, 0.53
0.40, 0.47
0.33, 0.40
0.27, 0.33
0.20, 0.27
0.13, 0.20
0.07, 0.13
0.00, 0.07
-0.07, 0.00
-0.13, -0.07
-0.20, -0.13
-0.27, -0.20
-0.33, -0.27
-0.40, -0.33
-0.47, -0.40
-0.53, -0.47
-0.60, -0.53
< -0.60
BALB/c IMQ
DBA IMQ
B6 IMQ
129/SvJ IMQ
MOLF IMQ
MOLF CTL
FVB IMQ
129/SvJ CTL
DBA CTL
B6 CTL
FVB CTL
BALB/c CTL
CD1 IMQ
CD1 CTL
Females
(C)
Principal Component 2 (15%)
Principal Component 1 (20.6%)
-10
0
10
20
30
Strain
B6
BALB/c
FVB
129/SvJ
DBA
CD1
MOLF
Group
CTL (M)
CTL (F)
IMQ (M)
IMQ (F)
(D)
Log10(P-value)
0
1
2
3
4
5
6
Gene Frequency
Strain
Treat
Sex
(E)
