# Supplementary material for: Imiquimod has strain-dependent effects in mice and does not uniquely model human psoriasis
Source: Genome Med. 2017 Mar 9;9:24. doi: 10.1186/s13073-017-0415-3 (PMC5345243; doi:10.1186/s13073-017-0415-3)
Supplement: Additional file 5: — Comparison of IMQ responses between males and females. (PDF 1350 kb) [file 13073_2017_415_MOESM5_ESM.pdf]

Additional File 5. Comparison of IMQ responses between males and females. (A – G) IMQ response comparison between males and females for each strain (*r* = Spearman rank correlation; dashed lines: robust linear regression estimates; ellipses: 90% of genes closest to the bivariate mean based on Mahalanobis distance). (H) Overlap of Mahalanobis distance ellipses for each strain. (I) Number of genes with significant sex-by-treatment interaction effects (FDR < 0.10; right margin: example genes with most significant interactions). (J) Number of genes with significant sex-by-treatment interaction effects (without FDR correction, P < 0.05). (K) Overlap of genes with sex-by-treatment interaction effects in FVB and MOLF strains (FDR < 0.10; top: IMQ-increased (males) and IMQ-decreased (females); bottom: IMQ-decreased (males) and IMQ-increased (females)). (L, M) Genes with the most consistent sex-by-treatment interaction patterns across strains (right margin: number of strains with shared pattern, without FDR correction, P < 0.05). (N – P) RT-PCR analysis of (N) *Cyp2g1*, (O) *Fosb* and (P) *Aldh1l2* expression (*n* = 5 per strain/sex/treatment group; *n* = 40 mice total). Groups without the same letter differ significantly (P < 0.05, Tukey honest significant difference; Error bars: standard error of the mean; p-values: sex-by-treatment interaction effects). *Rn18s* was used as an endogenous control to estimate relative gene expression.

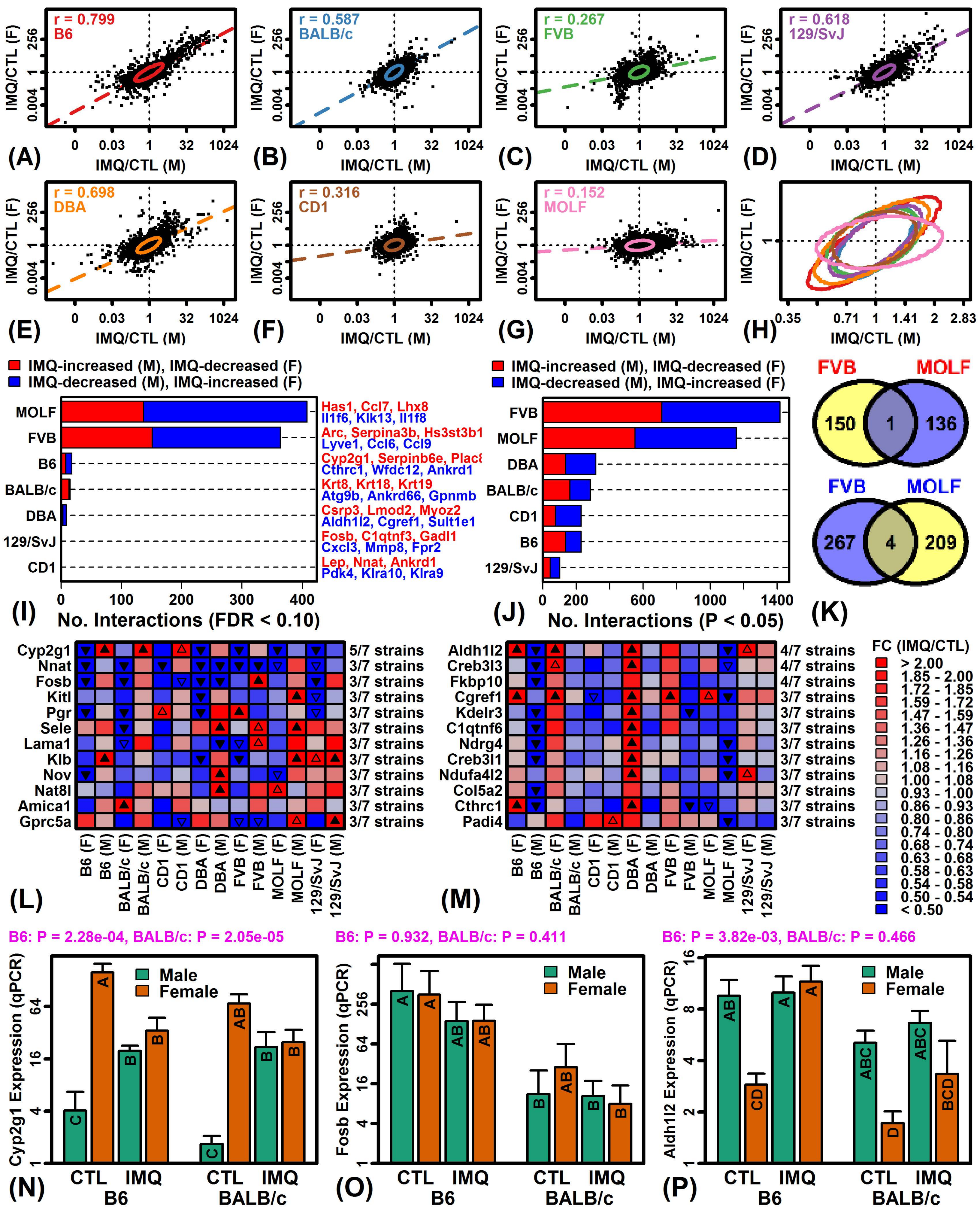

r = 0.799
B6
r = 0.587
BALB/c
r = 0.267
FVB
r = 0.618
129/SvJ
r = 0.698
DBA
r = 0.316
CD1
r = 0.152
MOLF
IMQ/CTL (F)
IMQ/CTL (M)
0.004
1
256
0
0.03
32
1024
0.35
0.71
1.41
2
2.83
(A)
(B)
(C)
(D)
(E)
(F)
(G)
(H)
IMQ-increased (M), IMQ-decreased (F)
IMQ-decreased (M), IMQ-increased (F)
MOLF
FVB
B6
BALB/c
DBA
129/SvJ
CD1
Has1, Ccl7, Lhx8
Il1f6, Klk13, Il1f8
Arc, Serpina3b, Hs3st3b1
Lyve1, Ccl6, Ccl9
Cyp2g1, Serpinb6e, Plac8
Cthrc1, Wfdc12, Ankrd1
Krt8, Krt18, Krt19
Atg9b, Ankrd66, Gpnmb
Csrp3, Lmod2, Myoz2
Aldh1l2, Cgref1, Sult1e1
Fosb, C1qtnf3, Gadl1
Cxcl3, Mmp8, Fpr2
Lep, Nnat, Ankrd1
Pdk4, Klra10, Klra9
0
100
200
300
400
(I)
No. Interactions (FDR < 0.10)
FVB
MOLF
DBA
BALB/c
CD1
B6
129/SvJ
0
200
600
1000
1400
(J)
No. Interactions (P < 0.05)
FVB
MOLF
150
1
136
FVB
MOLF
267
4
209
(K)
Cyp2g1
Nnat
Fosb
Kitl
Pgr
Sele
Lama1
Klb
Nov
Nat8l
Amica1
Gprc5a
5/7 strains
3/7 strains
B6 (F)
B6 (M)
BALB/c (F)
BALB/c (M)
CD1 (F)
CD1 (M)
DBA (F)
DBA (M)
FVB (F)
FVB (M)
MOLF (F)
MOLF (M)
129/SvJ (F)
129/SvJ (M)
(L)
Aldh1l2
Creb3l3
Fkbp10
Cgref1
Kdelr3
C1qtnf6
Ndrg4
Creb3l1
Ndufa4l2
Col5a2
Cthrc1
Padi4
4/7 strains
3/7 strains
(M)
FC (IMQ/CTL)
> 2.00
1.85 - 2.00
1.72 - 1.85
1.59 - 1.72
1.47 - 1.59
1.36 - 1.47
1.26 - 1.36
1.16 - 1.26
1.08 - 1.16
0.93 - 1.00
0.86 - 0.93
0.80 - 0.86
0.74 - 0.80
0.68 - 0.74
0.63 - 0.68
0.58 - 0.63
0.54 - 0.58
0.50 - 0.54
< 0.50
B6: P = 2.28e-04, BALB/c: P = 2.05e-05
Cyp2g1 Expression (qPCR)
Male
Female
1
4
16
64
A
B
AB
C
CTL
IMQ
B6
BALB/c
(N)
B6: P = 0.932, BALB/c: P = 0.411
Fosb Expression (qPCR)
1
4
16
64
256
(O)
B6: P = 3.82e-03, BALB/c: P = 0.466
Aldh1l2 Expression (qPCR)
1
2
4
8
16
ABC
CD
D
BCD
(P)
