# Supplementary material for: Imiquimod has strain-dependent effects in mice and does not uniquely model human psoriasis
Source: Genome Med. 2017 Mar 9;9:24. doi: 10.1186/s13073-017-0415-3 (PMC5345243; doi:10.1186/s13073-017-0415-3)
Supplement: Additional file 6: — Number of differentially expressed genes and associations with CTL body weight, epidermal thickness, and spleen weight. (PDF 286 kb) [file 13073_2017_415_MOESM6_ESM.pdf]

Additional File 6. Number of differentially expressed genes and associations with CTL body weight, epidermal thickness and spleen weight. Differentially expressed genes were identified for each strain-sex combination (FDR < 0.10; FC > 2.0 or FC < 0.5). The number of differentially expressed genes identified is shown (vertical axis) and compared to (A) CTL body weight, (B) CTL epidermal thickness and (C) CTL spleen weight (*r* = Spearman rank correlation; P = Spearman correlation p-value; dashed line: robust linear regression estimate).

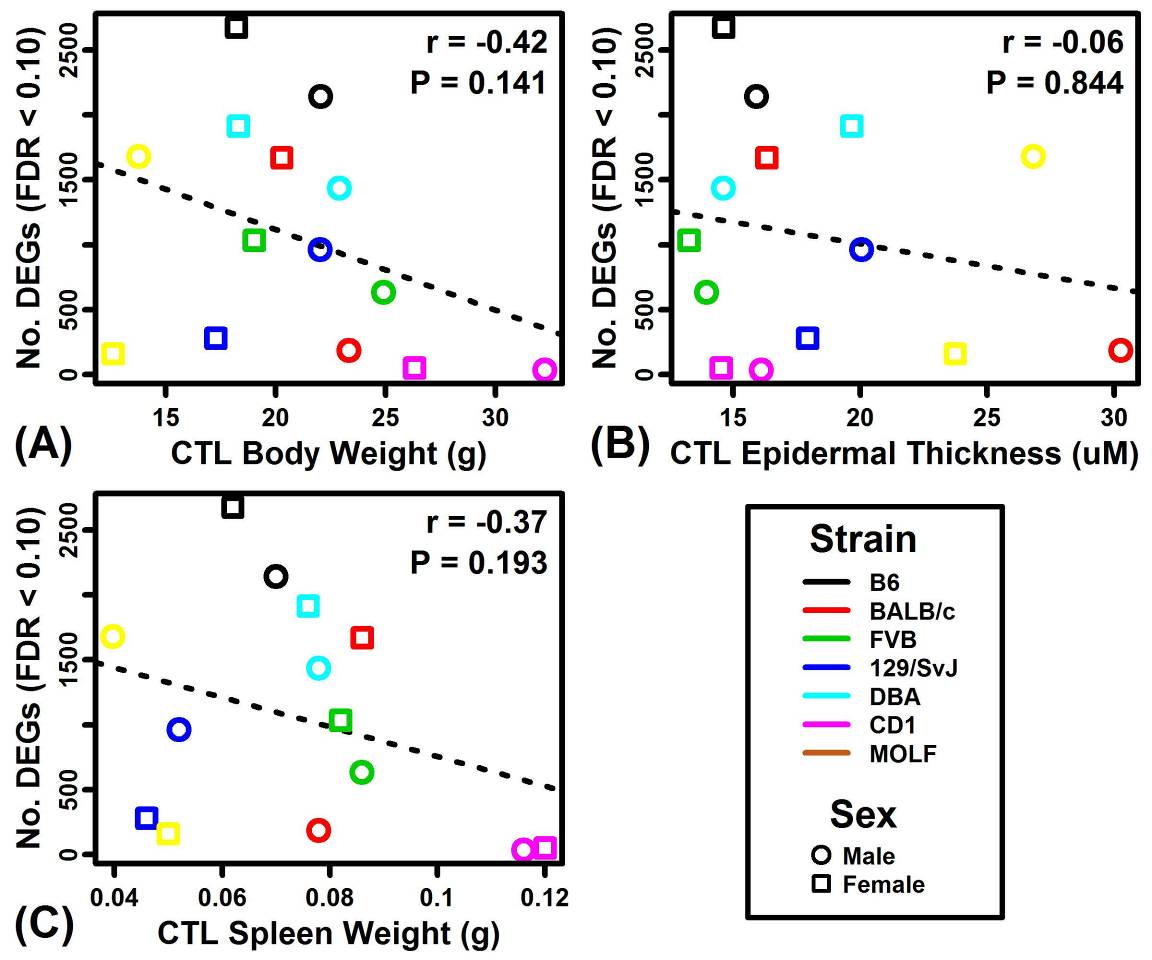

r = -0.42
P = 0.141
No. DEGs (FDR < 0.10)
0
500
1500
2500
15
20
25
30
(A)
CTL Body Weight (g)
r = -0.06
P = 0.844
(B)
CTL Epidermal Thickness (uM)
r = -0.37
P = 0.193
0.04
0.06
0.08
0.1
0.12
(C)
CTL Spleen Weight (g)
Strain
B6
BALB/c
FVB
129/SvJ
DBA
CD1
MOLF
Sex
Male
Female
