# Supplementary material for: Imiquimod has strain-dependent effects in mice and does not uniquely model human psoriasis
Source: Genome Med. 2017 Mar 9;9:24. doi: 10.1186/s13073-017-0415-3 (PMC5345243; doi:10.1186/s13073-017-0415-3)
Supplement: Additional file 7: — Microarray dataset comparison. (PDF 1604 kb) [file 13073_2017_415_MOESM7_ESM.pdf]

Additional File 7. Microarray dataset comparison. (A) Microarray datasets that have compared IMQ- and CTL-treated mouse skin. (B) RNA-seq vs. microarray correlation of FC estimates (rows: microarray datasets; columns: RNA-seq data from current study). (C) Overlap of genes elevated (top) or repressed (bottom) by IMQ in RNA-seq and microarray experiments (top: FC > 2.0 with FDR < 0.10; bottom: FC < 0.50 with FDR < 0.10). Microarray totals include genes altered in any of the 5 previous experiments. RNA-seq totals include genes altered in either sex of the B6 or BALB/c strains. (D) Gene density scatterplots comparing FC estimates (IMQ/CTL) from previous array studies (GSE27628 or GSE47607; horizontal axes) to RNA-seq estimates from B6, BALB/c or MOLF mice (vertical axes). (E) IMQ-increased DEGs identified by RNA-seq but not previously by microarray (FC > 2.0 with FDR < 0.10). (F) IMQ-decreased DEGs identified by RNA-seq but not previously by microarray (FC < 0.50 with FDR < 0.10).

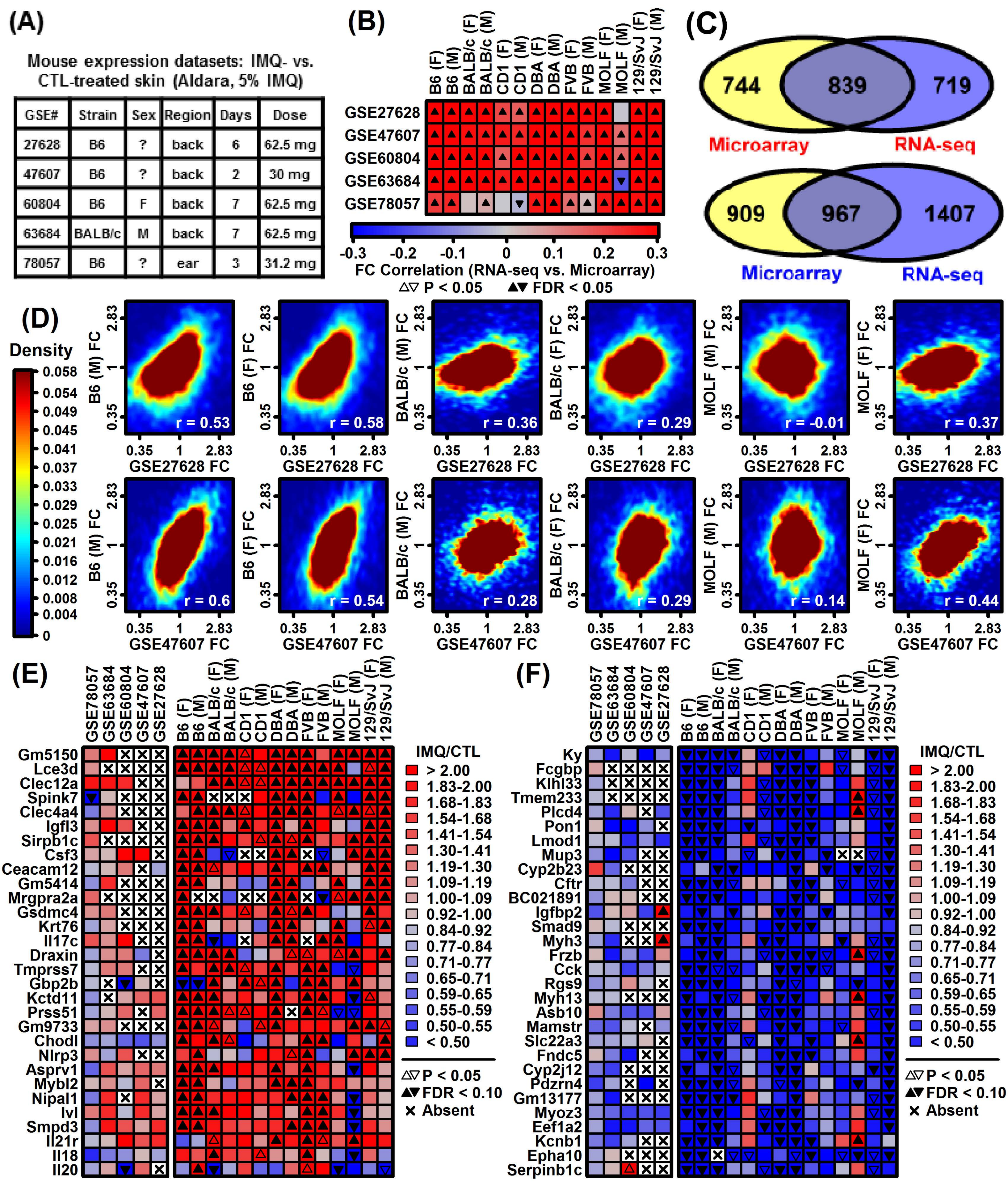
(A)
Mouse expression datasets: IMQ- vs. CTL-treated skin (Aldara, 5% IMQ)
GSE# | Strain | Sex | Region | Days | Dose
27628 | B6 | ? | back | 6 | 62.5 mg
47607 | B6 | ? | back | 2 | 30 mg
60804 | B6 | F | back | 7 | 62.5 mg
63684 | BALB/c | M | back | 7 | 62.5 mg
78057 | B6 | ? | ear | 3 | 31.2 mg
(B)
B6 (F) B6 (M) BALB/c (F) BALB/c (M) CD1 (F) CD1 (M) DBA (F) DBA (M) FVB (F) FVB (M) MOLF (F) MOLF (M) 129/SvJ (F) 129/SvJ (M)
GSE27628
GSE47607
GSE60804
GSE63684
GSE78057
-0.3 -0.2 -0.1 0 0.1 0.2 0.3
FC Correlation (RNA-seq vs. Microarray)
△▽ P < 0.05 ▲▼ FDR < 0.05
(C)
744 839 719
Microarray RNA-seq
909 967 1407
Microarray RNA-seq
(D)
Density
0.058 0.054 0.049 0.045 0.041 0.037 0.033 0.029 0.025 0.021 0.016 0.012 0.008 0.004 0
B6 (M) FC r = 0.53 GSE27628 FC
B6 (F) FC r = 0.58 GSE27628 FC
BALB/c (M) FC r = 0.36 GSE27628 FC
BALB/c (F) FC r = 0.29 GSE27628 FC
MOLF (M) FC r = -0.01 GSE27628 FC
MOLF (F) FC r = 0.37 GSE27628 FC
B6 (M) FC r = 0.6 GSE47607 FC
B6 (F) FC r = 0.54 GSE47607 FC
BALB/c (M) FC r = 0.28 GSE47607 FC
BALB/c (F) FC r = 0.29 GSE47607 FC
MOLF (M) FC r = 0.14 GSE47607 FC
MOLF (F) FC r = 0.44 GSE47607 FC
0.35 1 2.83
(E)
GSE78057 GSE63684 GSE60804 GSE47607 GSE27628
B6 (F) B6 (M) BALB/c (F) BALB/c (M) CD1 (F) CD1 (M) DBA (F) DBA (M) FVB (F) FVB (M) MOLF (F) MOLF (M) 129/SvJ (F) 129/SvJ (M)
Gm5150 Lce3d Clec12a Spink7 Clec4a4 Igfl3 Sirpb1c Csf3 Ceacam12 Gm5414 Mrgpra2a Gsdmc4 Krt76 Il17c Draxin Tmprss7 Gbp2b Kctd11 Prss51 Gm9733 Chodl Nlrp3 Asprv1 Mybl2 Nipal1 Ivl Smpd3 Il21r Il18 Il20
IMQ/CTL
> 2.00
1.83-2.00
1.68-1.83
1.54-1.68
1.41-1.54
1.30-1.41
1.19-1.30
1.09-1.19
1.00-1.09
0.92-1.00
0.84-0.92
0.77-0.84
0.71-0.77
0.65-0.71
0.59-0.65
0.55-0.59
0.50-0.55
< 0.50
△▽ P < 0.05
▲▼ FDR < 0.10
× Absent
(F)
GSE78057 GSE63684 GSE60804 GSE47607 GSE27628
B6 (F) B6 (M) BALB/c (F) BALB/c (M) CD1 (F) CD1 (M) DBA (F) DBA (M) FVB (F) FVB (M) MOLF (F) MOLF (M) 129/SvJ (F) 129/SvJ (M)
Ky Fcgbp Klhl33 Tmem233 Plcd4 Pon1 Lmod1 Mup3 Cyp2b23 Cftr BC021891 Igfbp2 Smad9 Myh3 Frzb Cck Rgs9 Myh13 Asb10 Mamstr Slc22a3 Fndc5 Cyp2j12 Pdzrn4 Gm13177 Myoz3 Eef1a2 Kcnb1 Epha10 Serpinb1c
IMQ/CTL
> 2.00
1.83-2.00
1.68-1.83
1.54-1.68
1.41-1.54
1.30-1.41
1.19-1.30
1.09-1.19
1.00-1.09
0.92-1.00
0.84-0.92
0.77-0.84
0.71-0.77
0.65-0.71
0.59-0.65
0.55-0.59
0.50-0.55
< 0.50
△▽ P < 0.05
▲▼ FDR < 0.10
× Absent
