# Supplementary material for: Imiquimod has strain-dependent effects in mice and does not uniquely model human psoriasis
Source: Genome Med. 2017 Mar 9;9:24. doi: 10.1186/s13073-017-0415-3 (PMC5345243; doi:10.1186/s13073-017-0415-3)
Supplement: Additional file 8: — Comparison of IMQ responses to human psoriasis lesions. (PDF 1593 kb) [file 13073_2017_415_MOESM8_ESM.pdf]

Additional File 8. Comparison of IMQ responses to human psoriasis lesions. (A) IMQ/psoriasis FC comparison. Gene density plots compare mouse FC estimates (IMQ/CTL) to psoriasis FC estimates (involved/uninvolved skin) for homologous genes. (B) Psoriasis-increased DEGs. Enrichment of psoriasis-increased DEGs was assessed relative to gene lists ranked according to IMQ response (*FDR < 0.05). (C) Psoriasis-decreased DEGs. Enrichment of psoriasis-decreased DEGs was assessed relative to gene lists ranked according to IMQ response (*FDR < 0.05). (E) Genes most strongly elevated in human psoriasis lesions. (F) Genes most strongly decreased in human psoriasis lesions.

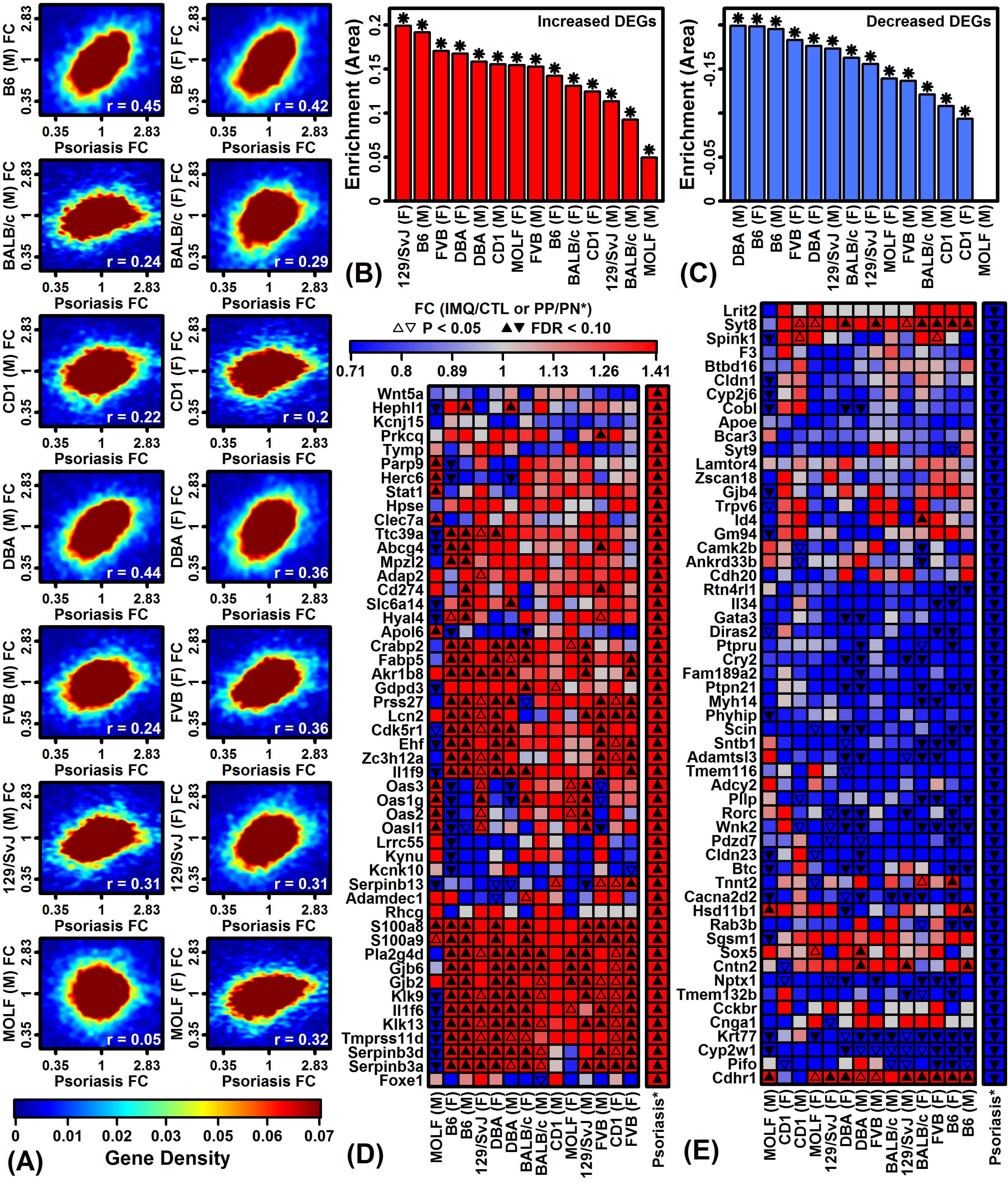

(A)
B6 (M) FC
B6 (F) FC
BALB/c (M) FC
BALB/c (F) FC
CD1 (M) FC
CD1 (F) FC
DBA (M) FC
DBA (F) FC
FVB (M) FC
FVB (F) FC
129/SvJ (M) FC
129/SvJ (F) FC
MOLF (M) FC
MOLF (F) FC
Psoriasis FC
0.35
1
2.83
r = 0.45
r = 0.42
r = 0.24
r = 0.29
r = 0.22
r = 0.2
r = 0.44
r = 0.36
r = 0.24
r = 0.36
r = 0.31
r = 0.31
r = 0.05
r = 0.32
0
0.01
0.02
0.03
0.05
0.06
0.07
Gene Density
(B)
Increased DEGs
Enrichment (Area)
0
0.05
0.1
0.15
0.2
129/SvJ (F)
B6 (M)
FVB (F)
DBA (F)
DBA (M)
CD1 (M)
MOLF (F)
FVB (M)
B6 (F)
BALB/c (F)
CD1 (F)
129/SvJ (M)
BALB/c (M)
MOLF (M)
(C)
Decreased DEGs
Enrichment (Area)
0
-0.05
-0.15
DBA (M)
B6 (F)
B6 (M)
FVB (F)
DBA (F)
129/SvJ (M)
BALB/c (F)
129/SvJ (F)
MOLF (F)
FVB (M)
BALB/c (M)
CD1 (M)
CD1 (F)
MOLF (M)
FC (IMQ/CTL or PP/PN*)
△▽ P < 0.05
▲▼ FDR < 0.10
0.71
0.8
0.89
1
1.13
1.26
1.41
(D)
Wnt5a
Hephl1
Kcnj15
Prkcq
Tymp
Parp9
Herc6
Stat1
Hpse
Clec7a
Ttc39a
Abcg4
Mpzl2
Adap2
Cd274
Slc6a14
Hyal4
Apol6
Crabp2
Fabp5
Akr1b8
Gdpd3
Prss27
Lcn2
Cdk5r1
Ehf
Zc3h12a
Il1f9
Oas3
Oas1g
Oas2
Oasl1
Lrrc55
Kynu
Kcnk10
Serpinb13
Adamdec1
Rhcg
S100a8
S100a9
Pla2g4d
Gjb6
Gjb2
Klk9
Il1f6
Klk13
Tmprss11d
Serpinb3d
Serpinb3a
Foxe1
MOLF (M)
B6 (F)
B6 (M)
129/SvJ (F)
DBA (F)
DBA (M)
BALB/c (F)
BALB/c (M)
CD1 (M)
MOLF (F)
129/SvJ (M)
FVB (M)
CD1 (F)
FVB (F)
Psoriasis*
(E)
Lrit2
Syt8
Spink1
F3
Btbd16
Cldn1
Cyp2j6
Cobl
Apoe
Bcar3
Syt9
Lamtor4
Zscan18
Gjb4
Trpv6
Id4
Gm94
Camk2b
Ankrd33b
Cdh20
Rtn4rl1
Il34
Gata3
Diras2
Ptpru
Cry2
Fam189a2
Ptpn21
Myh14
Phyhip
Scin
Sntb1
Adamtsl3
Tmem116
Adcy2
Pllp
Rorc
Wnk2
Pdzd7
Cldn23
Btc
Tnnt2
Cacna2d2
Hsd11b1
Rab3b
Sgsm1
Sox5
Cntn2
Nptx1
Tmem132b
Cckbr
Cnga1
Krt77
Cyp2w1
Pifo
Cdhr1
MOLF (M)
CD1 (F)
CD1 (M)
MOLF (F)
129/SvJ (F)
DBA (F)
DBA (M)
FVB (M)
BALB/c (M)
129/SvJ (M)
BALB/c (F)
FVB (F)
B6 (F)
B6 (M)
Psoriasis*
