# Supplementary material for: Imiquimod has strain-dependent effects in mice and does not uniquely model human psoriasis
Source: Genome Med. 2017 Mar 9;9:24. doi: 10.1186/s13073-017-0415-3 (PMC5345243; doi:10.1186/s13073-017-0415-3)
Supplement: Additional file 9: — Gene Ontology (GO) biological process (BP) terms enriched among psoriasis-decreased DEGs and correspondence with IMQ responses. (PDF 1635 kb) [file 13073_2017_415_MOESM9_ESM.pdf]

Additional File 9. Gene ontology (GO) biological process (BP) terms enriched among psoriasis-decreased DEGs and correspondence with IMQ responses. (A) Psoriasis-enriched GO BP terms. Heatmap colors depict average IMQ responses of 10 representative mouse homologues for each GO BP term. Chosen genes are homologous to the 10 human genes associated with the listed term for which expression was most strongly decreased in psoriasis lesions (i.e., lowest p-value). (B) Psoriasis-decreased DEGs associated with homeostatic GO BP terms (blue font in part (A)). (C) Psoriasis-decreased DEGs associated with transport GO BP terms (red font in part (A)). (D, E) RT-PCR analysis of (D) *Atp13a5* and (E) *P2rx1* expression ($n \geq 4$ per strain/sex/treatment group; $n = 38$ mice total). Groups without the same letter differ significantly ($P < 0.05$, Tukey honest significant difference; Error bars: standard error of the mean; p-values: strain-by-treatment interaction effect). *Rn18s* was used as an endogenous control to estimate relative gene expression.

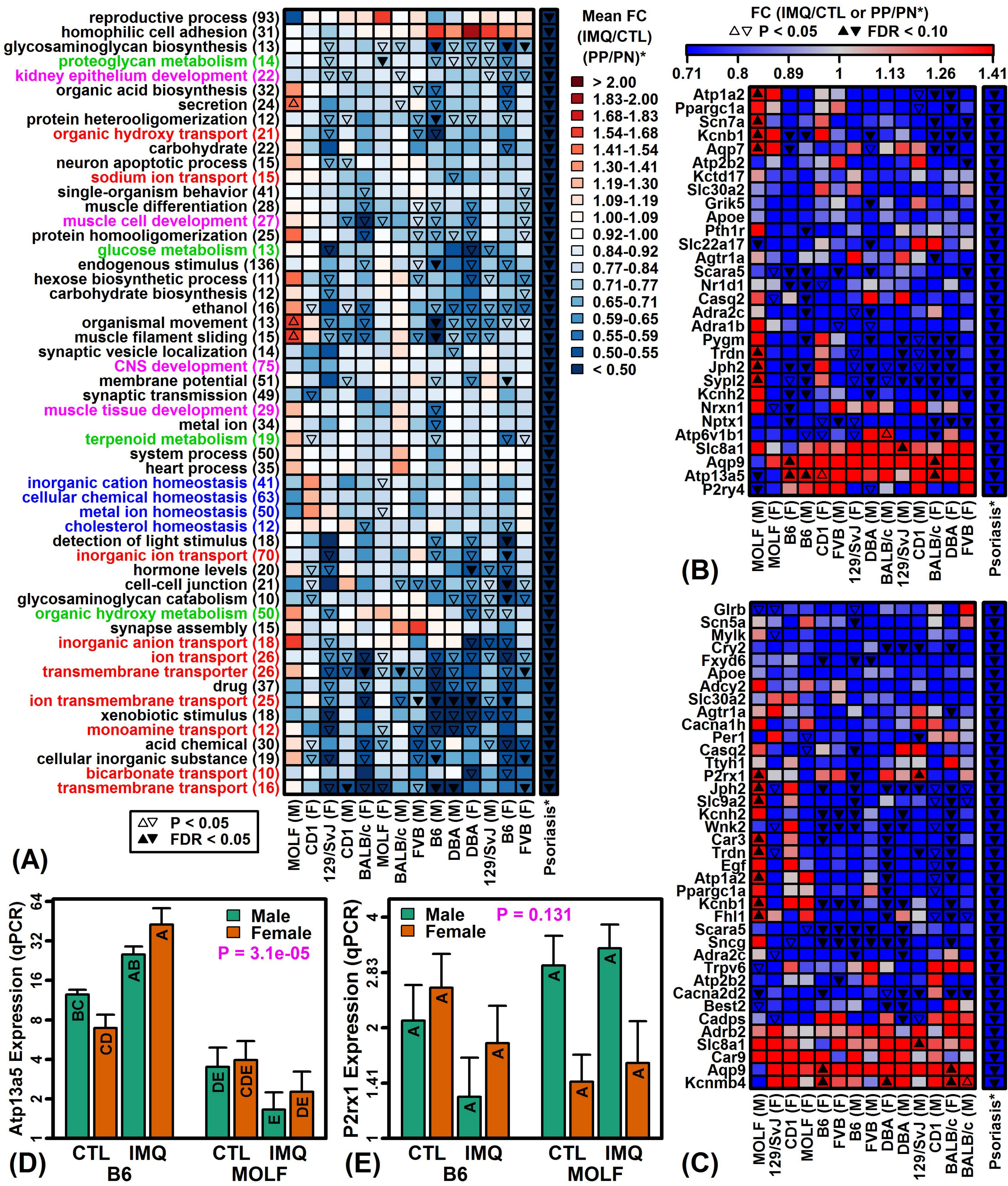

reproductive process (93)
homophilic cell adhesion (31)
glycosaminoglycan biosynthesis (13)
proteoglycan metabolism (14)
kidney epithelium development (22)
organic acid biosynthesis (32)
secretion (24)
protein heterooligomerization (12)
organic hydroxy transport (21)
carbohydrate (22)
neuron apoptotic process (15)
sodium ion transport (15)
single-organism behavior (41)
muscle differentiation (28)
muscle cell development (27)
protein homooligomerization (25)
glucose metabolism (13)
endogenous stimulus (136)
hexose biosynthetic process (11)
carbohydrate biosynthesis (12)
ethanol (16)
organismal movement (13)
muscle filament sliding (15)
synaptic vesicle localization (14)
CNS development (75)
membrane potential (51)
synaptic transmission (49)
muscle tissue development (29)
metal ion (34)
terpenoid metabolism (19)
system process (50)
heart process (35)
inorganic cation homeostasis (41)
cellular chemical homeostasis (63)
metal ion homeostasis (50)
cholesterol homeostasis (12)
detection of light stimulus (18)
inorganic ion transport (70)
hormone levels (20)
cell-cell junction (21)
glycosaminoglycan catabolism (10)
organic hydroxy metabolism (50)
synapse assembly (15)
inorganic anion transport (18)
ion transport (26)
transmembrane transporter (26)
drug (37)
ion transmembrane transport (25)
xenobiotic stimulus (18)
monoamine transport (12)
acid chemical (30)
cellular inorganic substance (19)
bicarbonate transport (10)
transmembrane transport (16)
MOLF (M) CD1 (F) 129/SvJ (F) CD1 (M) BALB/c (F) MOLF (F) BALB/c (M) FVB (M) B6 (M) DBA (M) DBA (F) 129/SvJ (M) B6 (F) FVB (F) Psoriasis*
△▽ P < 0.05
▲▼ FDR < 0.05
(A)
Mean FC (IMQ/CTL) (PP/PN)*
> 2.00
1.83-2.00
1.68-1.83
1.54-1.68
1.41-1.54
1.30-1.41
1.19-1.30
1.09-1.19
1.00-1.09
0.92-1.00
0.84-0.92
0.77-0.84
0.71-0.77
0.65-0.71
0.59-0.65
0.55-0.59
0.50-0.55
< 0.50
FC (IMQ/CTL or PP/PN*)
△▽ P < 0.05 ▲▼ FDR < 0.10
0.71 0.8 0.89 1 1.13 1.26 1.41
Atp1a2
Ppargc1a
Scn7a
Kcnb1
Aqp7
Atp2b2
Kctd17
Slc30a2
Grik5
Apoe
Pth1r
Slc22a17
Agtr1a
Scara5
Nr1d1
Casq2
Adra2c
Adra1b
Pygm
Trdn
Jph2
Sypl2
Kcnh2
Nrxn1
Nptx1
Atp6v1b1
Slc8a1
Aqp9
Atp13a5
P2ry4
MOLF (M) MOLF (F) B6 (F) B6 (M) CD1 (F) FVB (M) 129/SvJ (F) DBA (M) BALB/c (M) 129/SvJ (M) CD1 (M) BALB/c (F) DBA (F) FVB (F) Psoriasis*
(B)
Glrb
Scn5a
Mylk
Cry2
Fxyd6
Apoe
Adcy2
Slc30a2
Agtr1a
Cacna1h
Per1
Casq2
Ttyh1
P2rx1
Jph2
Slc9a2
Kcnh2
Wnk2
Car3
Trdn
Egf
Atp1a2
Ppargc1a
Kcnb1
Fhl1
Scara5
Sncg
Adra2c
Trpv6
Atp2b2
Cacna2d2
Best2
Cadps
Adrb2
Slc8a1
Car9
Aqp9
Kcnmb4
MOLF (M) 129/SvJ (F) CD1 (F) MOLF (F) B6 (F) FVB (F) B6 (M) FVB (M) DBA (F) DBA (M) 129/SvJ (M) CD1 (M) BALB/c (F) BALB/c (M) Psoriasis*
(C)
Atp13a5 Expression (qPCR)
64 32 16 8 4 2 1
Male
Female
P = 3.1e-05
BC CD AB A DE CDE E DE
CTL IMQ CTL IMQ
B6 MOLF
(D)
P2rx1 Expression (qPCR)
4 2.83 2 1.41 1
Male
Female
P = 0.131
A A A A A A A A
CTL IMQ CTL IMQ
B6 MOLF
(E)
