# Supplementary material for: Imiquimod has strain-dependent effects in mice and does not uniquely model human psoriasis
Source: Genome Med. 2017 Mar 9;9:24. doi: 10.1186/s13073-017-0415-3 (PMC5345243; doi:10.1186/s13073-017-0415-3)
Supplement: Additional file 10: — IL-17 family gene expression (Il17a, Il17b, Il17c, Il17d, Il17e, Il17f). (PDF 506 kb) [file 13073_2017_415_MOESM10_ESM.pdf]

Additional File 10. IL-17 family gene expression (*Il17a*, *Il17b*, *Il17c*, *Il17d*, *Il17e*, *Il17f*). RT-PCR was used to evaluate relative gene expression of IL-17 family genes in B6 and BALB/c mice (*n* = 5 per strain/sex/treatment group; *n* = 40 mice total). Groups without the same letter differ significantly ($P < 0.05$, Tukey honest significant difference; Error bars: standard error of the mean; p-values: strain-by-treatment interaction effect). *Rn18s* was used as an endogenous control to estimate relative gene expression.

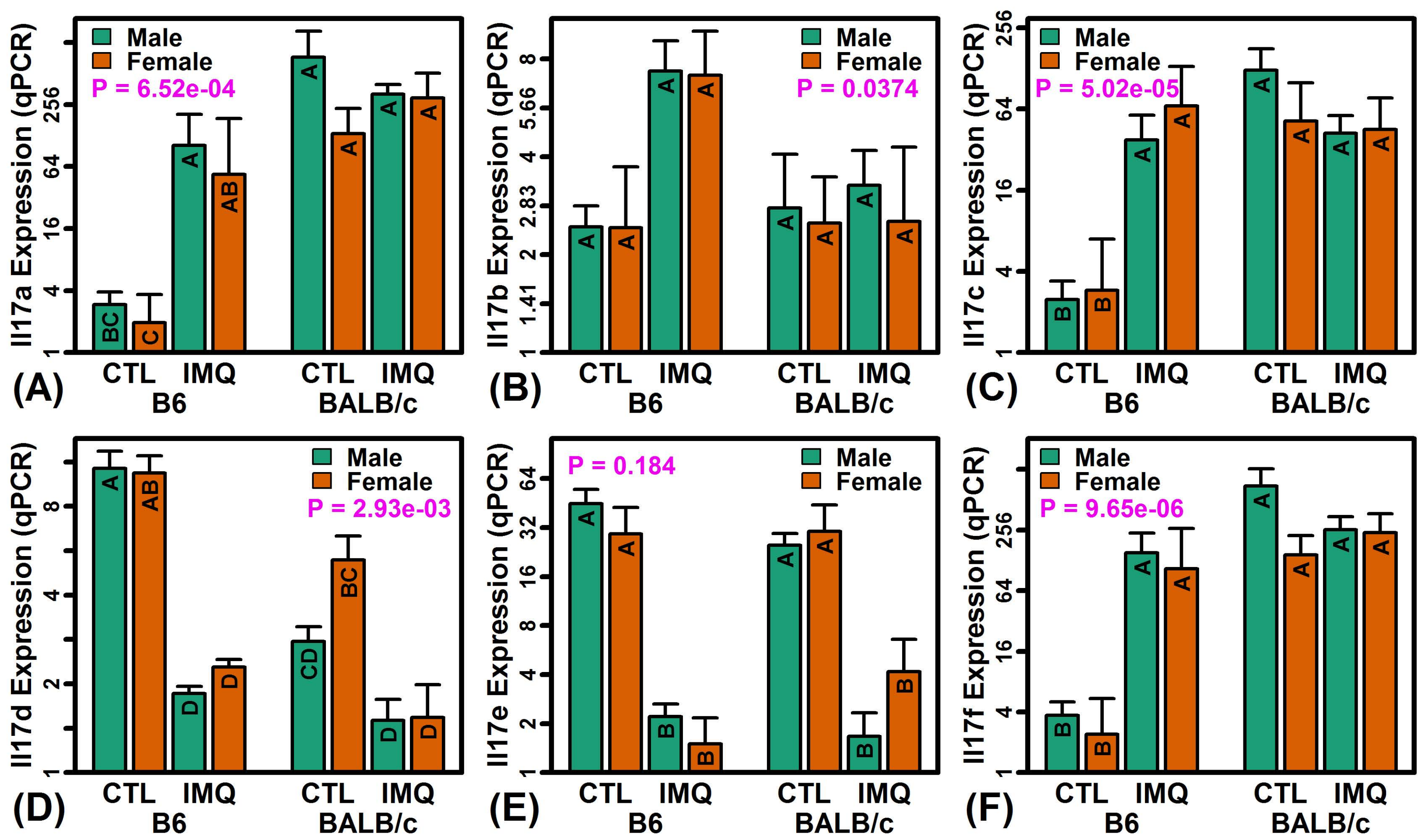
(A)
Il17a Expression (qPCR)
Male
Female
P = 6.52e-04
(B)
Il17b Expression (qPCR)
P = 0.0374
(C)
Il17c Expression (qPCR)
P = 5.02e-05
(D)
Il17d Expression (qPCR)
P = 2.93e-03
(E)
Il17e Expression (qPCR)
P = 0.184
(F)
Il17f Expression (qPCR)
P = 9.65e-06
CTL
IMQ
B6
BALB/c
