# Supplementary material for: Imiquimod has strain-dependent effects in mice and does not uniquely model human psoriasis
Source: Genome Med. 2017 Mar 9;9:24. doi: 10.1186/s13073-017-0415-3 (PMC5345243; doi:10.1186/s13073-017-0415-3)
Supplement: Additional file 11: — Genes uniquely altered by IMQ in MOLF males. (PDF 1511 kb) [file 13073_2017_415_MOESM11_ESM.pdf]

Additional File 11. Genes uniquely altered by IMQ in MOLF males. (A) Genes uniquely increased by IMQ in MOLF males. (B) Genes uniquely decreased by IMQ in MOLF males. (C, D) RT-PCR analysis of (C) *Il1f5* and (D) *Krt1* expression ($n \geq 4$ per strain/sex/treatment group; $n = 38$ mice total). Groups without the same letter differ significantly ($P < 0.05$, Tukey honest significant difference; Error bars: standard error of the mean; p-values: strain-by-treatment interaction effect). *Rn18s* was used as an endogenous control to estimate relative gene expression. (E) GO BP terms enriched among genes uniquely increased by IMQ in MOLF males (right margin: exemplar IMQ-increased genes). (F) GO BP terms enriched among genes uniquely decreased by IMQ in MOLF males (right margin: exemplar IMQ-decreased genes).

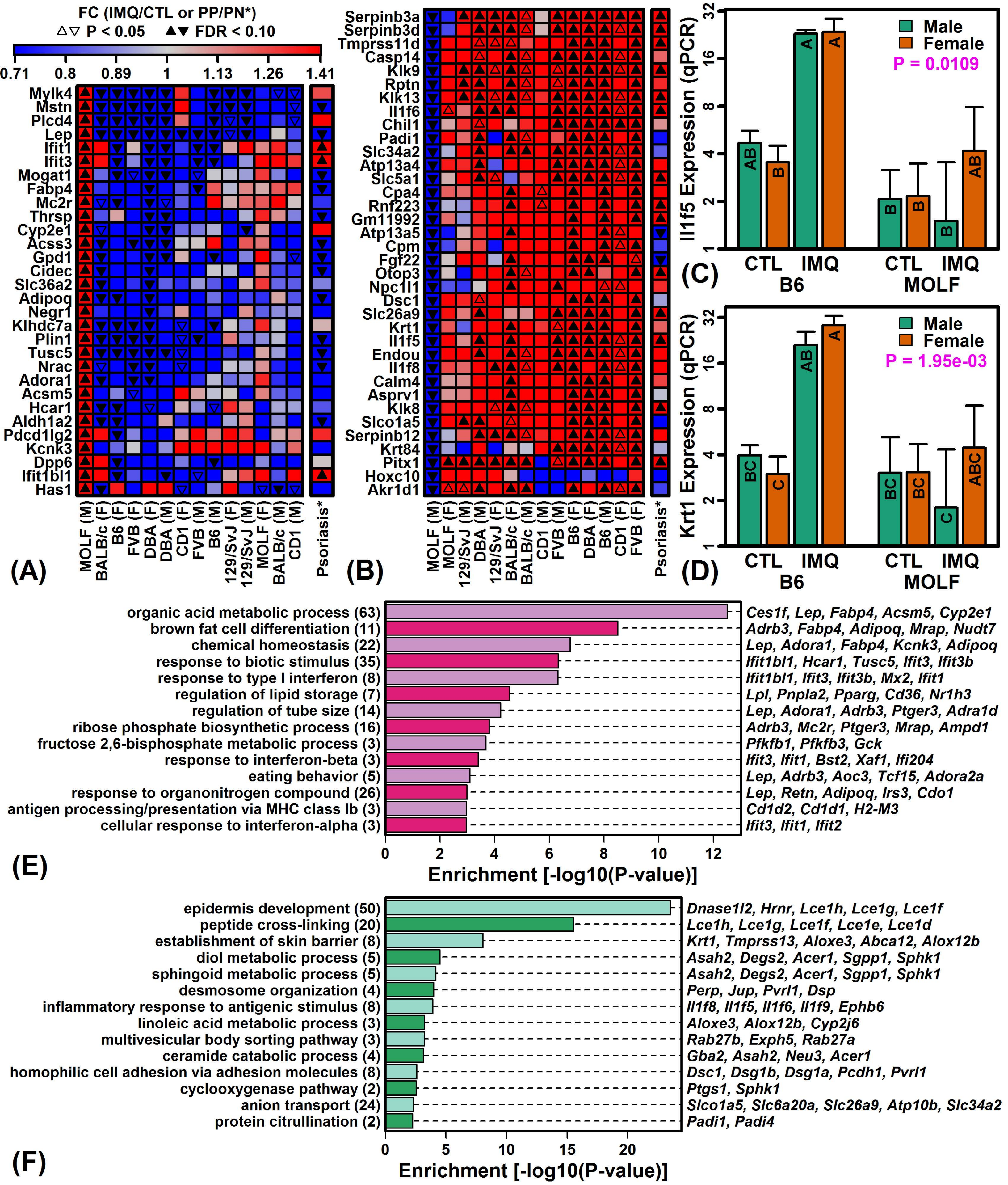

FC (IMQ/CTL or PP/PN*)
△▽ P < 0.05 ▲▼ FDR < 0.10
0.71 0.8 0.89 1 1.13 1.26 1.41
Mylk4
Mstn
Plcd4
Lep
Ifit1
Ifit3
Mogat1
Fabp4
Mc2r
Thrsp
Cyp2e1
Acss3
Gpd1
Cidec
Slc36a2
Adipoq
Negr1
Klhdc7a
Plin1
Tusc5
Nrac
Adora1
Acsm5
Hcar1
Aldh1a2
Pdcd1lg2
Kcnk3
Dpp6
Ifit1bl1
Has1
MOLF (M)
BALB/c (F)
B6 (F)
FVB (F)
DBA (F)
DBA (M)
CD1 (F)
FVB (M)
B6 (M)
129/SvJ (F)
129/SvJ (M)
MOLF (F)
BALB/c (M)
CD1 (M)
Psoriasis*
(A)
Serpinb3a
Serpinb3d
Tmprss11d
Casp14
Klk9
Rptn
Klk13
Il1f6
Chil1
Padi1
Slc34a2
Atp13a4
Slc5a1
Cpa4
Rnf223
Gm11992
Atp13a5
Cpm
Fgf22
Otop3
Npc1l1
Dsc1
Slc26a9
Krt1
Il1f5
Endou
Il1f8
Calm4
Asprv1
Klk8
Slco1a5
Serpinb12
Krt84
Pitx1
Hoxc10
Akr1d1
MOLF (M)
MOLF (F)
129/SvJ (M)
DBA (M)
129/SvJ (F)
BALB/c (F)
BALB/c (M)
CD1 (M)
FVB (M)
B6 (F)
DBA (F)
B6 (M)
CD1 (F)
FVB (F)
Psoriasis*
(B)
Il1f5 Expression (qPCR)
1 2 4 8 16 32
Male
Female
P = 0.0109
AB B A A B B B AB
CTL IMQ CTL IMQ
B6 MOLF
(C)
Krt1 Expression (qPCR)
1 2 4 8 16 32
Male
Female
P = 1.95e-03
BC C AB A BC BC C ABC
CTL IMQ CTL IMQ
B6 MOLF
(D)
organic acid metabolic process (63)
brown fat cell differentiation (11)
chemical homeostasis (22)
response to biotic stimulus (35)
response to type I interferon (8)
regulation of lipid storage (7)
regulation of tube size (14)
ribose phosphate biosynthetic process (16)
fructose 2,6-bisphosphate metabolic process (3)
response to interferon-beta (3)
eating behavior (5)
response to organonitrogen compound (26)
antigen processing/presentation via MHC class Ib (3)
cellular response to interferon-alpha (3)
Ces1f, Lep, Fabp4, Acsm5, Cyp2e1
Adrb3, Fabp4, Adipoq, Mrap, Nudt7
Lep, Adora1, Fabp4, Kcnk3, Adipoq
Ifit1bl1, Hcar1, Tusc5, Ifit3, Ifit3b
Ifit1bl1, Ifit3, Ifit3b, Mx2, Ifit1
Lpl, Pnpla2, Pparg, Cd36, Nr1h3
Lep, Adora1, Adrb3, Ptger3, Adra1d
Adrb3, Mc2r, Ptger3, Mrap, Ampd1
Pfkfb1, Pfkfb3, Gck
Ifit3, Ifit1, Bst2, Xaf1, Ifi204
Lep, Adrb3, Aoc3, Tcf15, Adora2a
Lep, Retn, Adipoq, Irs3, Cdo1
Cd1d2, Cd1d1, H2-M3
Ifit3, Ifit1, Ifit2
0 2 4 6 8 10 12
Enrichment [-log10(P-value)]
(E)
epidermis development (50)
peptide cross-linking (20)
establishment of skin barrier (8)
diol metabolic process (5)
sphingoid metabolic process (5)
desmosome organization (4)
inflammatory response to antigenic stimulus (8)
linoleic acid metabolic process (3)
multivesicular body sorting pathway (3)
ceramide catabolic process (4)
homophilic cell adhesion via adhesion molecules (8)
cyclooxygenase pathway (2)
anion transport (24)
protein citrullination (2)
Dnase1l2, Hrnr, Lce1h, Lce1g, Lce1f
Lce1h, Lce1g, Lce1f, Lce1e, Lce1d
Krt1, Tmprss13, Aloxe3, Abca12, Alox12b
Asah2, Degs2, Acer1, Sgpp1, Sphk1
Asah2, Degs2, Acer1, Sgpp1, Sphk1
Perp, Jup, Pvrl1, Dsp
Il1f8, Il1f5, Il1f6, Il1f9, Ephb6
Aloxe3, Alox12b, Cyp2j6
Rab27b, Exph5, Rab27a
Gba2, Asah2, Neu3, Acer1
Dsc1, Dsg1b, Dsg1a, Pcdh1, Pvrl1
Ptgs1, Sphk1
Slco1a5, Slc6a20a, Slc26a9, Atp10b, Slc34a2
Padi1, Padi4
0 5 10 15 20
Enrichment [-log10(P-value)]
(F)
