# Supplementary material for: Imiquimod has strain-dependent effects in mice and does not uniquely model human psoriasis
Source: Genome Med. 2017 Mar 9;9:24. doi: 10.1186/s13073-017-0415-3 (PMC5345243; doi:10.1186/s13073-017-0415-3)
Supplement: Additional file 12: — Genes associated with Toll-like receptor (TLR) signaling are disproportionately elevated by IMQ in all strains. (PDF 1173 kb) [file 13073_2017_415_MOESM12_ESM.pdf]

Additional File 12. Genes associated with toll-like receptor (TLR) signaling are disproportionately elevated by IMQ in all strains. (A) TLR gene set enrichment analysis (GSEA). GSEA was performed using 101 TLR-associated genes (KEGG database; pathway ID mmu04620). Enrichment greater than zero indicates bias of TLR genes towards IMQ-increased expression (asterisk: FDR < 0.05, Wilcoxon rank sum test). (B) 129/SvJ females (GSEA analysis). Genes were ranked based upon IMQ response (horizontal axis) and cumulative overlap with the ranked gene list was evaluated (vertical axis). The area between the cumulative overlap curve and diagonal (red) corresponds to the enrichment statistic in part (A). 86 of 101 TLR genes with detectable expression were included in the analysis. (C) CD1 males (GSEA analysis). (D) TLR pathway genes most frequently altered across strains. (E - R) KEGG TLR pathway maps (red: IMQ-increased; yellow: IMQ-decreased).

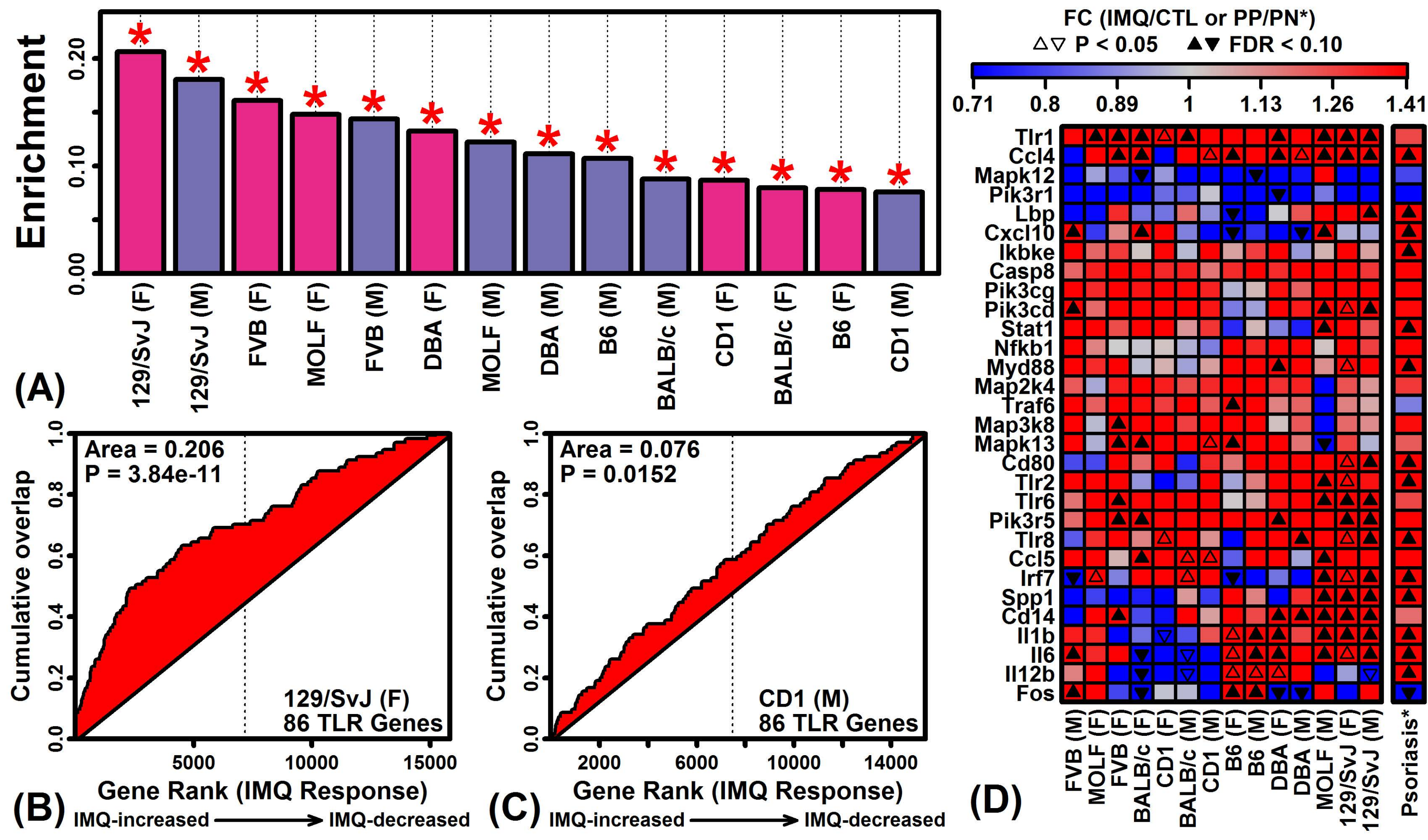

Enrichment
129/SvJ (F)
129/SvJ (M)
FVB (F)
MOLF (F)
FVB (M)
DBA (F)
MOLF (M)
DBA (M)
B6 (M)
BALB/c (M)
CD1 (F)
BALB/c (F)
B6 (F)
CD1 (M)
(A)
Area = 0.206
P = 3.84e-11
129/SvJ (F)
86 TLR Genes
Cumulative overlap
Gene Rank (IMQ Response)
(B)
IMQ-increased
IMQ-decreased
Area = 0.076
P = 0.0152
CD1 (M)
86 TLR Genes
(C)
FC (IMQ/CTL or PP/PN*)
P < 0.05
FDR < 0.10
Tlr1
Ccl4
Mapk12
Pik3r1
Lbp
Cxcl10
Ikbke
Casp8
Pik3cg
Pik3cd
Stat1
Nfkb1
Myd88
Map2k4
Traf6
Map3k8
Mapk13
Cd80
Tlr2
Tlr6
Pik3r5
Tlr8
Ccl5
Irf7
Spp1
Cd14
Il1b
Il6
Il12b
Fos
Psoriasis*
(D)

(E) B6 (M)

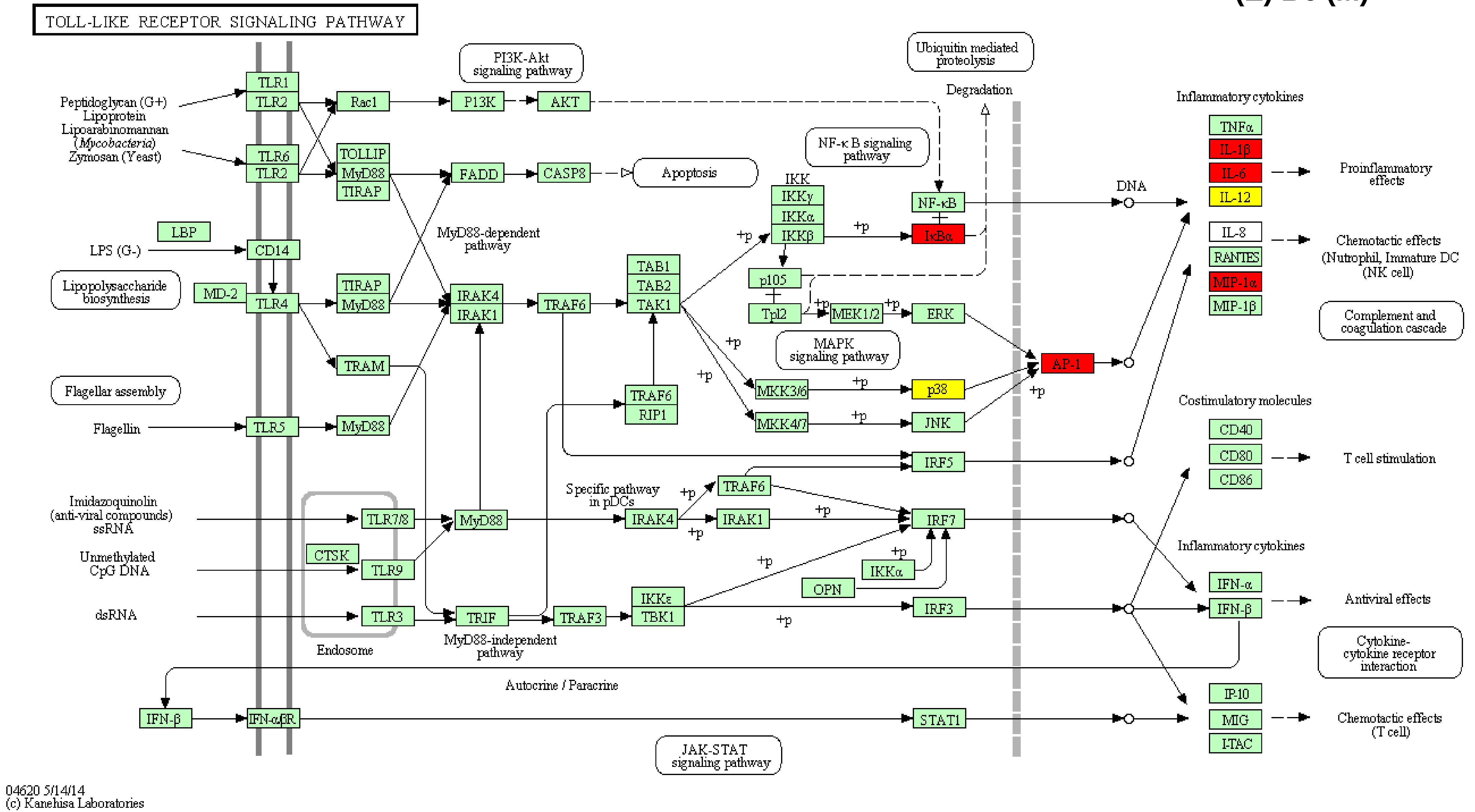

TOLL-LIKE RECEPTOR SIGNALING PATHWAY
Peptidoglycan (G+)
Lipoprotein
Lipoarabinomannan
(Mycobacteria)
Zymosan (Yeast)
TLR1
TLR2
TLR6
TLR2
Rac1
PI3K-Akt signaling pathway
PI3K
AKT
Ubiquitin mediated proteolysis
Degradation
TOLLIP
MyD88
TIRAP
FADD
CASP8
Apoptosis
NF-κB signaling pathway
IKK
IKKγ
IKKα
IKKβ
NF-κB
IκBα
+p
LBP
LPS (G-)
CD14
MD-2
TLR4
Lipopolysaccharide biosynthesis
TIRAP
MyD88
MyD88-dependent pathway
IRAK4
IRAK1
TRAF6
TAB1
TAB2
TAK1
p105
Tpl2
MEK1/2
ERK
MAPK signaling pathway
TRAM
Flagellar assembly
Flagellin
TLR5
MyD88
TRAF6
RIP1
MKK3/6
p38
MKK4/7
JNK
AP-1
DNA
IRF5
Inflammatory cytokines
TNFα
IL-1β
IL-6
IL-12
Proinflammatory effects
IL-8
RANTES
MIP-1α
MIP-1β
Chemotactic effects
(Nutrophil, Immature DC
(NK cell)
Complement and coagulation cascade
Costimulatory molecules
CD40
CD80
CD86
T cell stimulation
Imidazoquinolin
(anti-viral compounds)
ssRNA
TLR7/8
MyD88
Specific pathway in pDCs
IRAK4
TRAF6
IRAK1
IRF7
Unmethylated CpG DNA
CTSK
TLR9
IKKα
OPN
dsRNA
TLR3
Endosome
TRIF
MyD88-independent pathway
TRAF3
IKKε
TBK1
IRF3
Inflammatory cytokines
IFN-α
IFN-β
Antiviral effects
Cytokine-cytokine receptor interaction
Autocrine / Paracrine
IFN-β
IFN-α/βR
STAT1
JAK-STAT signaling pathway
IP-10
MIG
I-TAC
Chemotactic effects
(T cell)
04620 5/14/14
(c) Kanehisa Laboratories

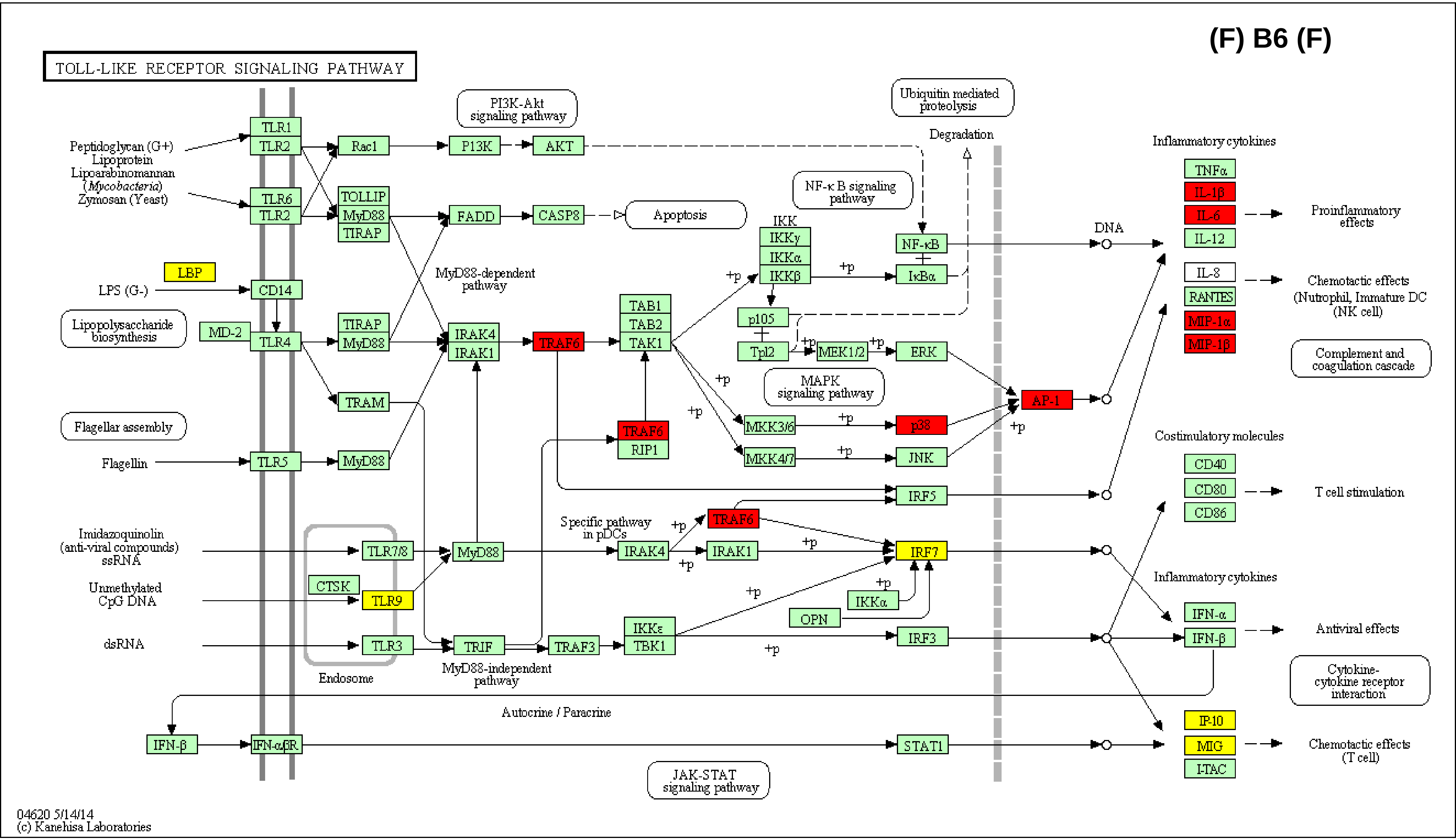
(F) B6 (F)
TOLL-LIKE RECEPTOR SIGNALING PATHWAY
Peptidoglycan (G+)
Lipoprotein
Lipoarabinomannan
(Mycobacteria)
Zymosan (Yeast)
LBP
LPS (G-)
Lipopolysaccharide biosynthesis
MD-2
Flagellar assembly
Flagellin
Imidazoquinolin (anti-viral compounds) ssRNA
Unmethylated CpG DNA
dsRNA
TLR1
TLR2
TLR6
TLR2
CD14
TLR4
TLR5
Rac1
TOLLIP
MyD88
TIRAP
TIRAP
MyD88
TRAM
MyD88
PI3K-Akt signaling pathway
PI3K
AKT
FADD
CASP8
Apoptosis
MyD88-dependent pathway
IRAK4
IRAK1
TRAF6
TAB1
TAB2
TAK1
TRAF6
RIP1
Endosome
TLR7/8
CTSK
TLR9
TLR3
MyD88
TRIF
MyD88-independent pathway
TRAF3
Specific pathway in pDCs
IRAK4
TRAF6
IRAK1
IKKε
TBK1
OPN
IKKα
IKK
IKKγ
IKKα
IKKβ
p105
Tpl2
MEK1/2
ERK
NF-κB signaling pathway
Ubiquitin mediated proteolysis
Degradation
NF-κB
IκBα
MAPK signaling pathway
MKK3/6
p38
MKK4/7
JNK
AP-1
IRF5
IRF7
IRF3
DNA
+p
Inflammatory cytokines
TNFα
IL-1β
IL-6
IL-12
Proinflammatory effects
IL-8
RANTES
MIP-1α
MIP-1β
Chemotactic effects (Nutrophil, Immature DC (NK cell)
Complement and coagulation cascade
Costimulatory molecules
CD40
CD80
CD86
T cell stimulation
Inflammatory cytokines
IFN-α
IFN-β
Antiviral effects
Cytokine-cytokine receptor interaction
IP-10
MIG
I-TAC
Chemotactic effects (T cell)
Autocrine / Paracrine
IFN-β
IFN-α/βR
STAT1
JAK-STAT signaling pathway
04620 5/14/14
(c) Kanehisa Laboratories

(G) BALB/c (M)

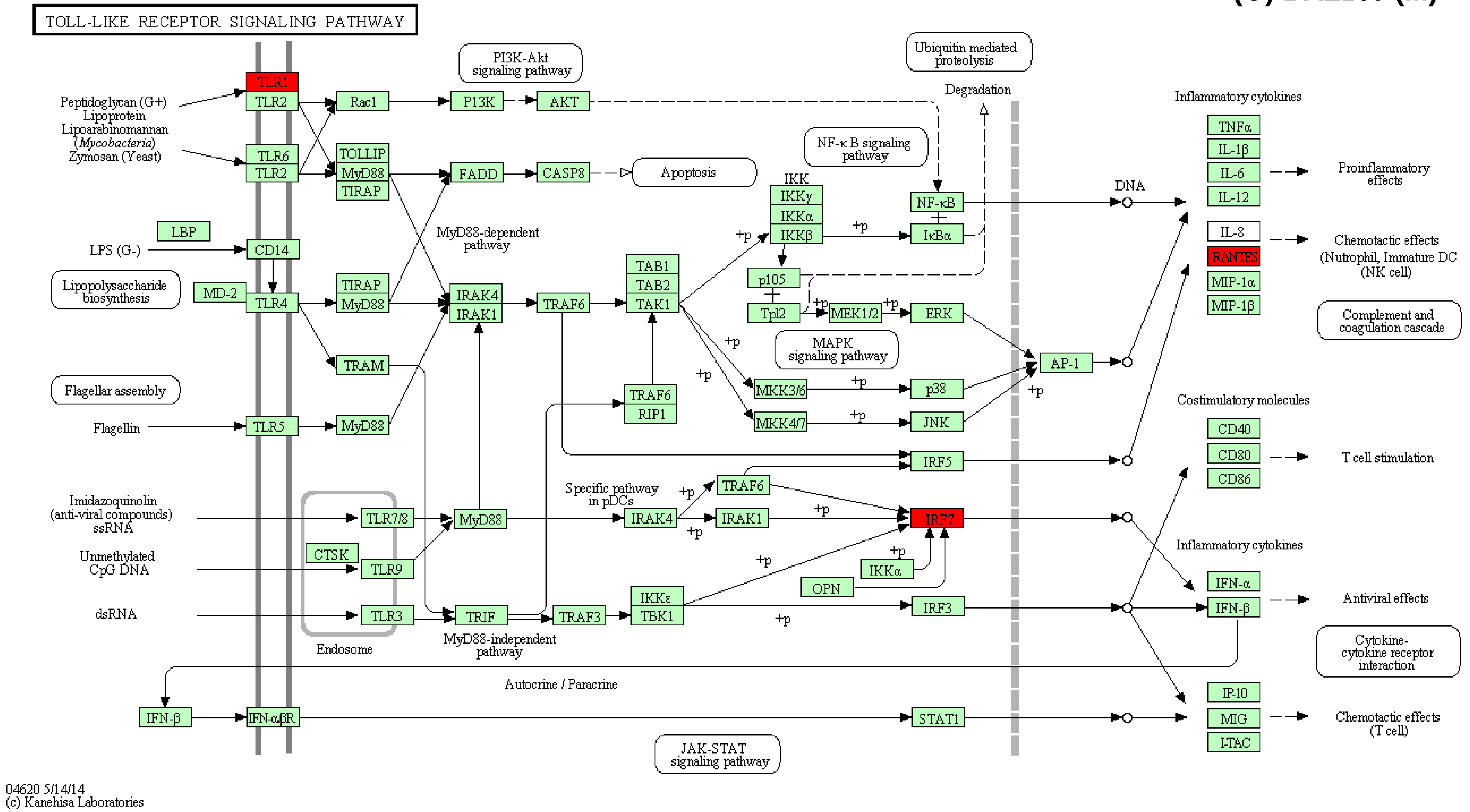

TOLL-LIKE RECEPTOR SIGNALING PATHWAY
Peptidoglycan (G+)
Lipoprotein
Lipoarabinomannan
(Mycobacteria)
Zymosan (Yeast)
TLR1
TLR2
TLR6
TLR2
Rac1
PI3K
AKT
PI3K-Akt signaling pathway
TOLLIP
MyD88
TIRAP
FADD
CASP8
Apoptosis
LBP
LPS (G-)
CD14
MD-2
TLR4
Lipopolysaccharide biosynthesis
TIRAP
MyD88
MyD88-dependent pathway
IRAK4
IRAK1
TRAF6
TAB1
TAB2
TAK1
TRAM
Flagellar assembly
Flagellin
TLR5
MyD88
TRAF6
RIP1
IKK
IKKγ
IKKα
IKKβ
NF-κB signaling pathway
Ubiquitin mediated proteolysis
Degradation
NF-κB
IκBα
p105
Tpl2
MEK1/2
ERK
MAPK signaling pathway
MKK3/6
p38
MKK4/7
JNK
AP-1
+p
DNA
IRF5
Imidazoquinolin
(anti-viral compounds)
ssRNA
Unmethylated
CpG DNA
dsRNA
TLR7/8
CTSK
TLR9
TLR3
Endosome
MyD88
TRIF
MyD88-independent pathway
Specific pathway in pDCs
IRAK4
IRAK1
TRAF6
IRF7
IKKα
OPN
TRAF3
IKKε
TBK1
IRF3
Autocrine / Paracrine
IFN-β
IFN-α/βR
STAT1
JAK-STAT signaling pathway
Inflammatory cytokines
TNFα
IL-1β
IL-6
IL-12
Proinflammatory effects
IL-8
RANTES
MIP-1α
MIP-1β
Chemotactic effects
(Nutrophil, Immature DC
(NK cell)
Complement and coagulation cascade
Costimulatory molecules
CD40
CD80
CD86
T cell stimulation
Inflammatory cytokines
IFN-α
IFN-β
Antiviral effects
Cytokine-cytokine receptor interaction
IP-10
MIG
I-TAC
Chemotactic effects
(T cell)
04620 5/14/14
(c) Kanehisa Laboratories

(H) BALB/c (F)

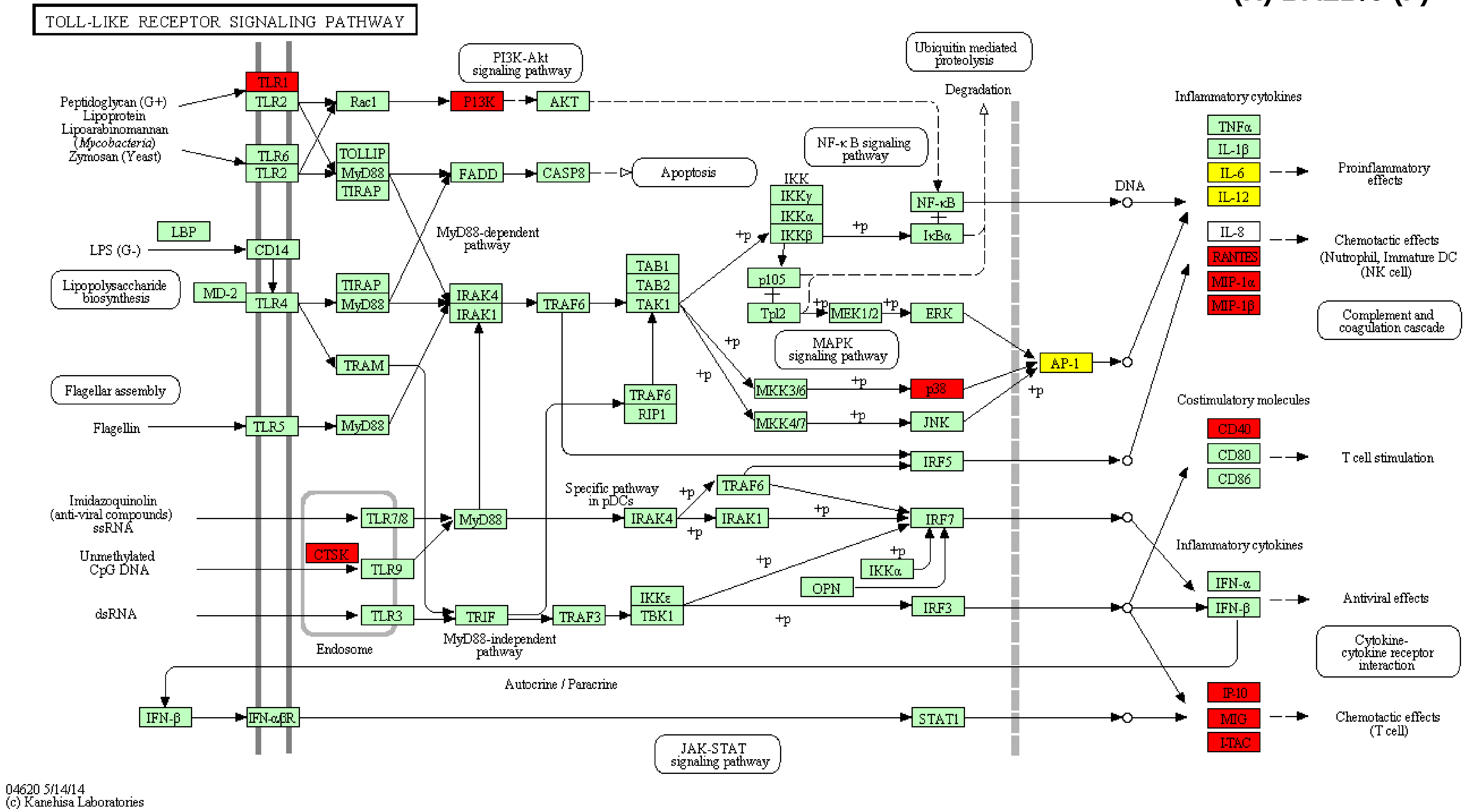

TOLL-LIKE RECEPTOR SIGNALING PATHWAY
Peptidoglycan (G+)
Lipoprotein
Lipoarabinomannan
(Mycobacteria)
Zymosan (Yeast)
TLR1
TLR2
TLR6
TLR2
Rac1
PI3K
AKT
PI3K-Akt signaling pathway
TOLLIP
MyD88
TIRAP
FADD
CASP8
Apoptosis
LBP
LPS (G-)
CD14
Lipopolysaccharide biosynthesis
MD-2
TLR4
TIRAP
MyD88
MyD88-dependent pathway
IRAK4
IRAK1
TRAF6
TAB1
TAB2
TAK1
TRAM
Flagellar assembly
Flagellin
TLR5
MyD88
TRAF6
RIP1
IKK
IKKγ
IKKα
IKKβ
NF-κB signaling pathway
Ubiquitin mediated proteolysis
Degradation
NF-κB
IκBα
p105
Tpl2
MEK1/2
ERK
MAPK signaling pathway
MKK3/6
p38
MKK4/7
JNK
AP-1
+p
DNA
IRF5
Imidazoquinolin
(anti-viral compounds)
ssRNA
TLR7/8
MyD88
Specific pathway in pDCs
IRAK4
TRAF6
IRAK1
IRF7
Unmethylated CpG DNA
CTSK
TLR9
IKKα
OPN
dsRNA
TLR3
Endosome
TRIF
MyD88-independent pathway
TRAF3
IKKε
TBK1
IRF3
Autocrine / Paracrine
IFN-β
IFN-α/βR
STAT1
JAK-STAT signaling pathway
Inflammatory cytokines
TNFα
IL-1β
IL-6
IL-12
Proinflammatory effects
IL-8
RANTES
MIP-1α
MIP-1β
Chemotactic effects
(Nutrophil, Immature DC (NK cell)
Complement and coagulation cascade
Costimulatory molecules
CD40
CD80
CD86
T cell stimulation
Inflammatory cytokines
IFN-α
IFN-β
Antiviral effects
Cytokine-cytokine receptor interaction
IP-10
MIG
I-TAC
Chemotactic effects (T cell)
04620 5/14/14
(c) Kanehisa Laboratories

(I) CD1 (M)

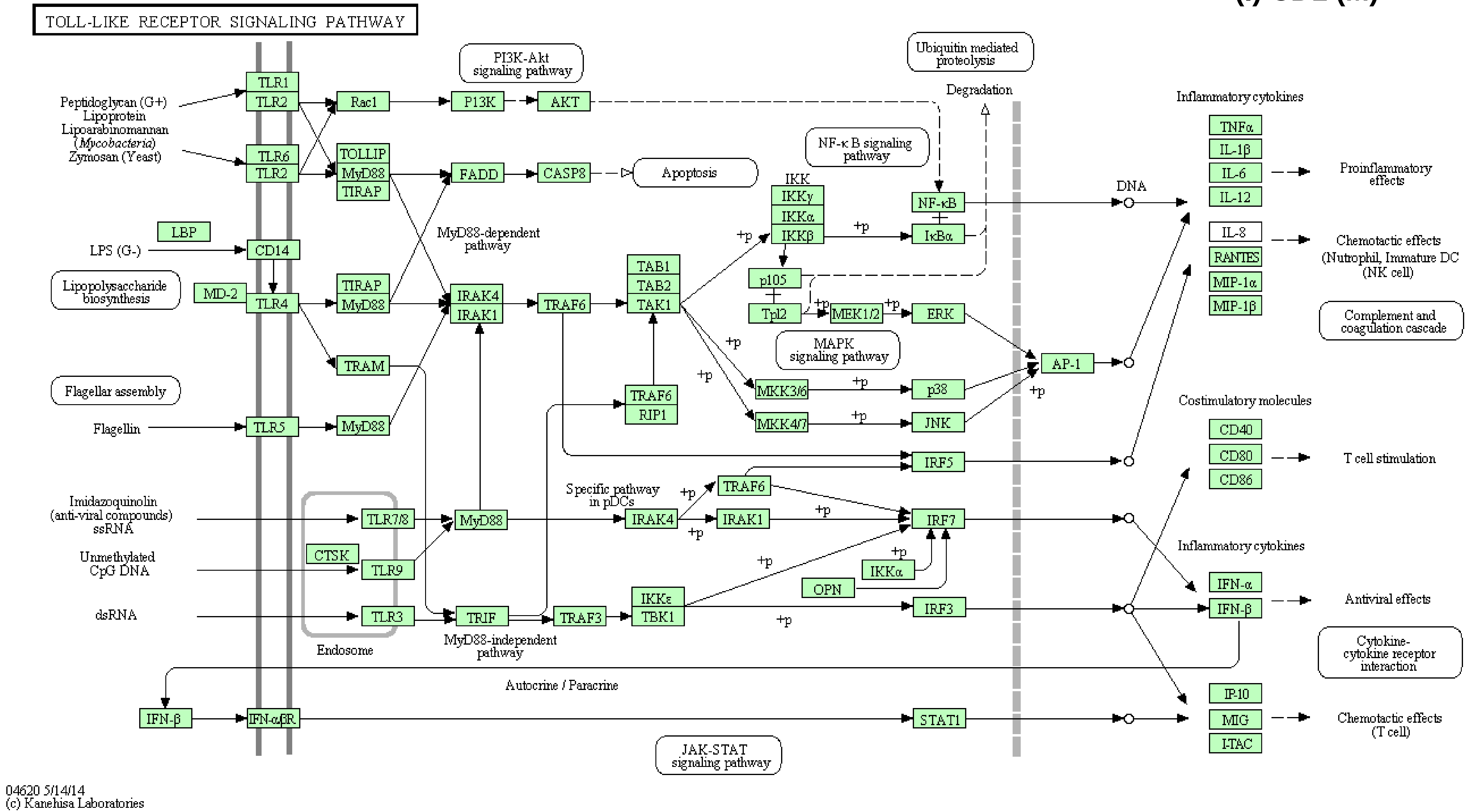
TOLL-LIKE RECEPTOR SIGNALING PATHWAY
Peptidoglycan (G+)
Lipoprotein
Lipoarabinomannan
(*Mycobacteria*)
Zymosan (Yeast)
TLR1
TLR2
TLR6
TLR2
Rac1
PI3K-Akt signaling pathway
PI3K
AKT
TOLLIP
MyD88
TIRAP
FADD
CASP8
Apoptosis
Ubiquitin mediated proteolysis
Degradation
NF-κB signaling pathway
IKK
IKKγ
IKKα
IKKβ
NF-κB
IκBα
LBP
LPS (G-)
CD14
Lipopolysaccharide biosynthesis
MD-2
TLR4
TIRAP
MyD88
MyD88-dependent pathway
IRAK4
IRAK1
TRAF6
TAB1
TAB2
TAK1
p105
Tpl2
MEK1/2
ERK
+p
MAPK signaling pathway
TRAM
Flagellar assembly
Flagellin
TLR5
MyD88
TRAF6
RIP1
MKK3/6
p38
MKK4/7
JNK
AP-1
DNA
Inflammatory cytokines
TNFα
IL-1β
IL-6
IL-12
Proinflammatory effects
IL-8
RANTES
MIP-1α
MIP-1β
Chemotactic effects
(Nutrophil, Immature DC (NK cell)
Complement and coagulation cascade
Costimulatory molecules
CD40
CD80
CD86
T cell stimulation
IRF5
Specific pathway in pDCs
TRAF6
IRAK4
IRAK1
IRF7
Imidazoquinolin
(anti-viral compounds)
ssRNA
TLR7/8
MyD88
CTSK
Unmethylated CpG DNA
TLR9
IKKα
OPN
dsRNA
TLR3
TRIF
TRAF3
IKKε
TBK1
IRF3
Endosome
MyD88-independent pathway
Inflammatory cytokines
IFN-α
IFN-β
Antiviral effects
Cytokine-cytokine receptor interaction
Autocrine / Paracrine
IFN-β
IFN-α/βR
STAT1
JAK-STAT signaling pathway
IP-10
MIG
I-TAC
Chemotactic effects (T cell)
04620 5/14/14
(c) Kanehisa Laboratories

(J) CD1 (F)

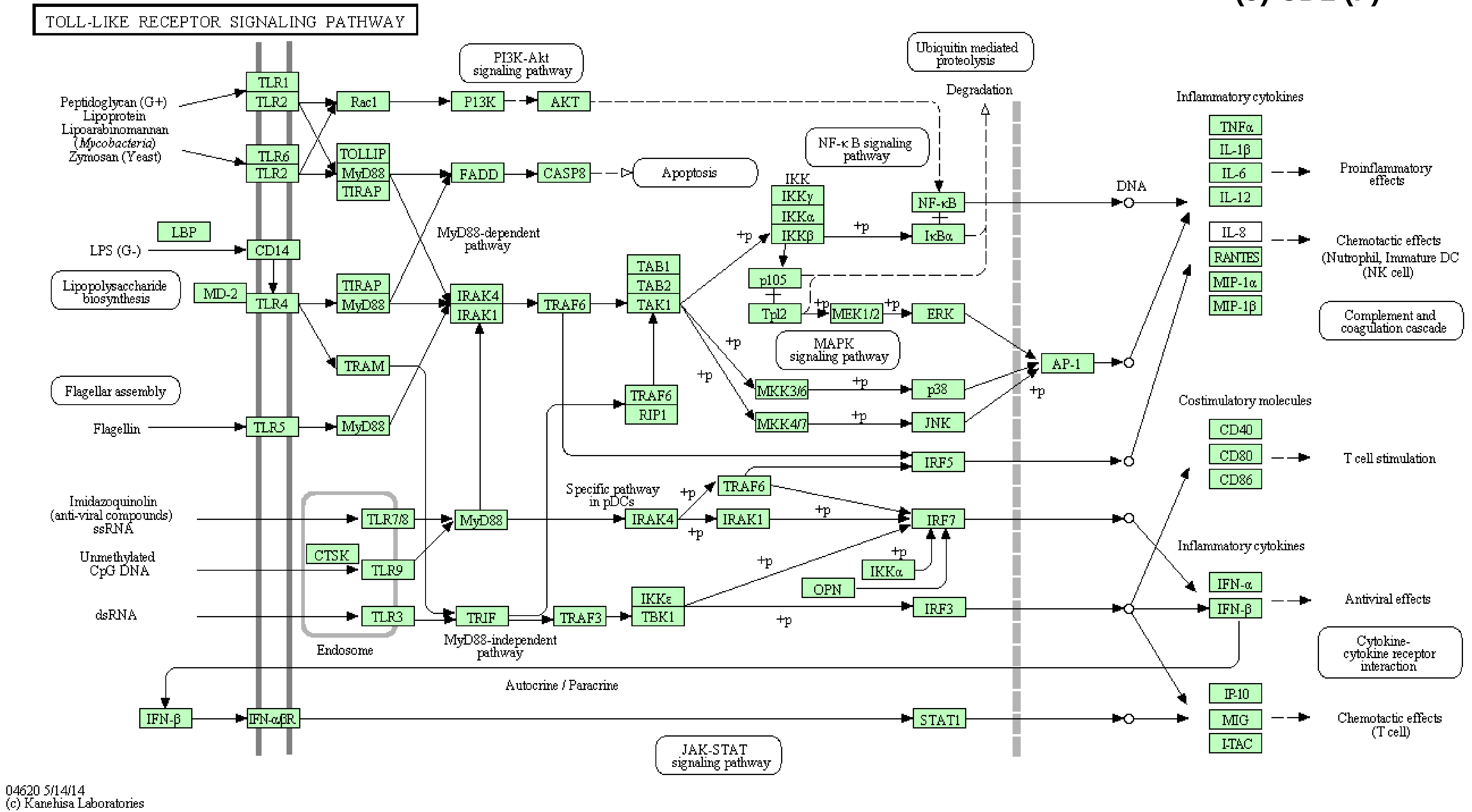
TOLL-LIKE RECEPTOR SIGNALING PATHWAY
Peptidoglycan (G+)
Lipoprotein
Lipoarabinomannan
(Mycobacteria)
Zymosan (Yeast)
TLR1
TLR2
TLR6
TLR2
Rac1
TOLLIP
MyD88
TIRAP
PI3K-Akt signaling pathway
PI3K
AKT
FADD
CASP8
Apoptosis
Ubiquitin mediated proteolysis
Degradation
NF-κB signaling pathway
IKK
IKKγ
IKKα
IKKβ
NF-κB
IκBα
+p
LBP
LPS (G-)
CD14
Lipopolysaccharide biosynthesis
MD-2
TLR4
TIRAP
MyD88
MyD88-dependent pathway
IRAK4
IRAK1
TRAF6
TAB1
TAB2
TAK1
p105
Tpl2
MEK1/2
ERK
MAPK signaling pathway
TRAM
Flagellar assembly
Flagellin
TLR5
MyD88
TRAF6
RIP1
MKK3/6
MKK4/7
p38
JNK
AP-1
DNA
Inflammatory cytokines
TNFα
IL-1β
IL-6
IL-12
Proinflammatory effects
IL-8
RANTES
MIP-1α
MIP-1β
Chemotactic effects
(Nutrophil, Immature DC
(NK cell)
Complement and coagulation cascade
Costimulatory molecules
CD40
CD80
CD86
T cell stimulation
IRF5
Specific pathway in pDCs
TRAF6
IRAK4
IRAK1
IRF7
Imidazoquinolin
(anti-viral compounds)
ssRNA
TLR7/8
MyD88
CTSK
TLR9
Unmethylated CpG DNA
IKKα
OPN
dsRNA
TLR3
Endosome
TRIF
MyD88-independent pathway
TRAF3
IKKε
TBK1
IRF3
Inflammatory cytokines
IFN-α
IFN-β
Antiviral effects
Cytokine-cytokine receptor interaction
Autocrine / Paracrine
IFN-β
IFN-α/βR
STAT1
JAK-STAT signaling pathway
IP-10
MIG
I-TAC
Chemotactic effects
(T cell)
04620 5/14/14
(c) Kanehisa Laboratories

(K) DBA (M)

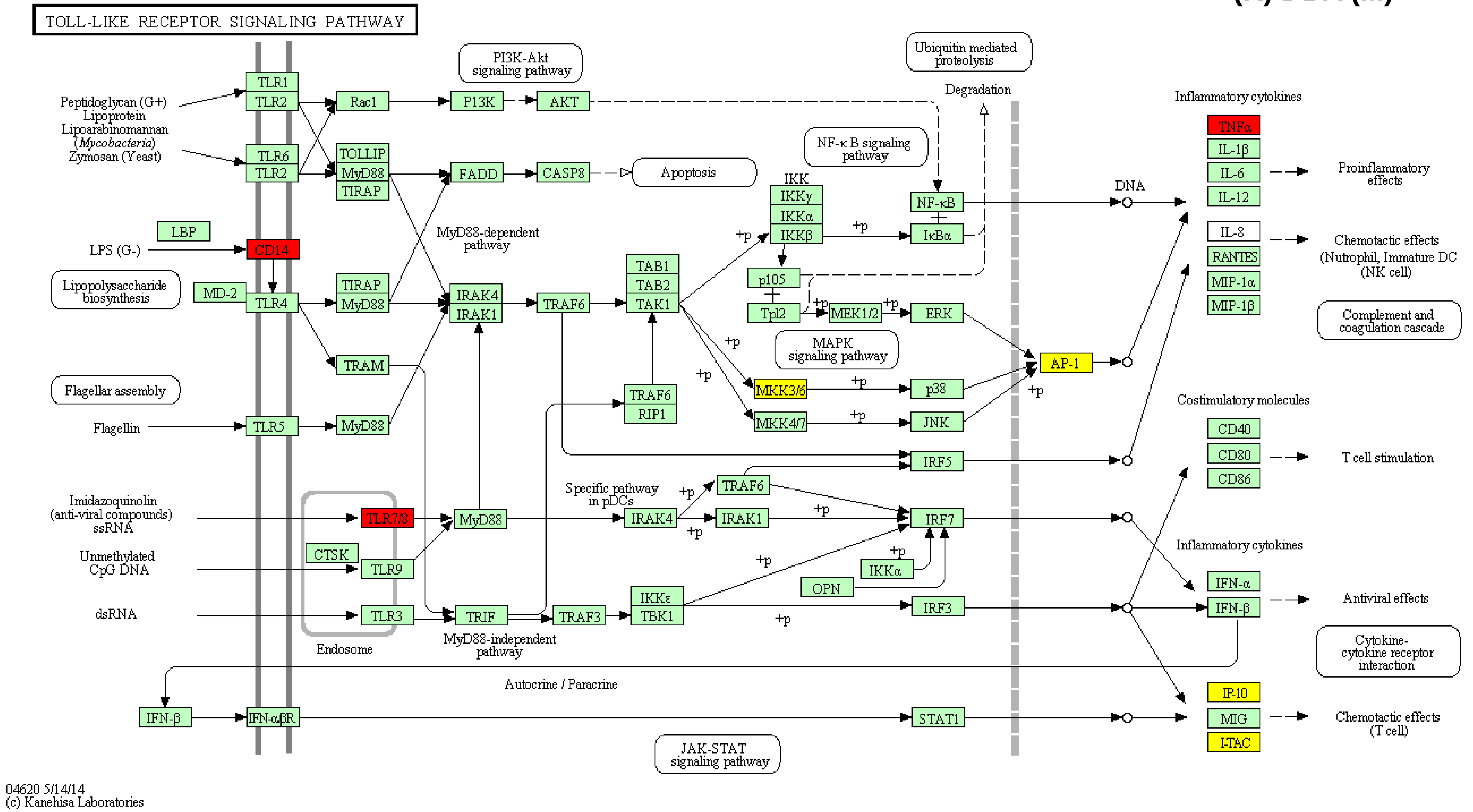

TOLL-LIKE RECEPTOR SIGNALING PATHWAY
Peptidoglycan (G+)
Lipoprotein
Lipoarabinomannan
(Mycobacteria)
Zymosan (Yeast)
TLR1
TLR2
TLR6
TLR2
Rac1
PI3K-Akt signaling pathway
PI3K
AKT
TOLLIP
MyD88
TIRAP
FADD
CASP8
Apoptosis
Ubiquitin mediated proteolysis
Degradation
NF-κB signaling pathway
IKK
IKKγ
IKKα
IKKβ
NF-κB
IκBα
Inflammatory cytokines
TNFα
IL-1β
IL-6
IL-12
Proinflammatory effects
DNA
IL-8
RANTES
MIP-1α
MIP-1β
Chemotactic effects (Nutrophil, Immature DC (NK cell)
Complement and coagulation cascade
LBP
LPS (G-)
CD14
Lipopolysaccharide biosynthesis
MD-2
TLR4
TIRAP
MyD88
MyD88-dependent pathway
IRAK4
IRAK1
TRAF6
TAB1
TAB2
TAK1
p105
Tpl2
MEK1/2
ERK
MAPK signaling pathway
AP-1
TRAM
Flagellar assembly
TRAF6
RIP1
MKK3/6
p38
MKK4/7
JNK
Flagellin
TLR5
MyD88
Costimulatory molecules
CD40
CD80
CD86
T cell stimulation
IRF5
Imidazoquinolin (anti-viral compounds) ssRNA
TLR7/8
MyD88
Specific pathway in pDCs
IRAK4
TRAF6
IRAK1
IRF7
Inflammatory cytokines
CTSK
Unmethylated CpG DNA
TLR9
IKKα
OPN
IFN-α
IFN-β
Antiviral effects
dsRNA
TLR3
TRIF
TRAF3
IKKε
TBK1
IRF3
Endosome
MyD88-independent pathway
Cytokine-cytokine receptor interaction
Autocrine / Paracrine
IFN-β
IFN-α/βR
STAT1
IP-10
MIG
I-TAC
Chemotactic effects (T cell)
JAK-STAT signaling pathway
+p
04620 5/14/14
(c) Kanehisa Laboratories

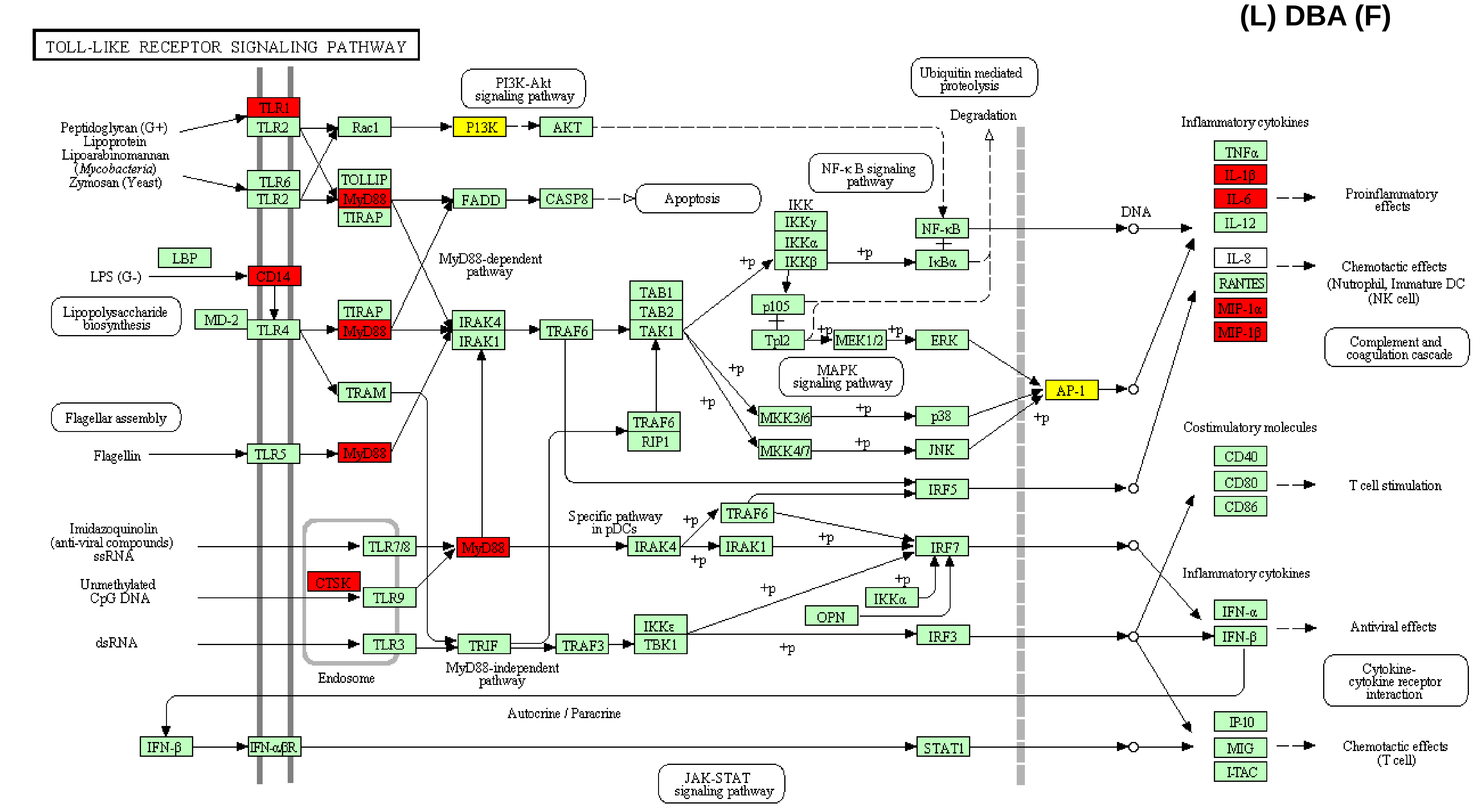

04620 5/14/14

(c) Kanehisa Laboratories

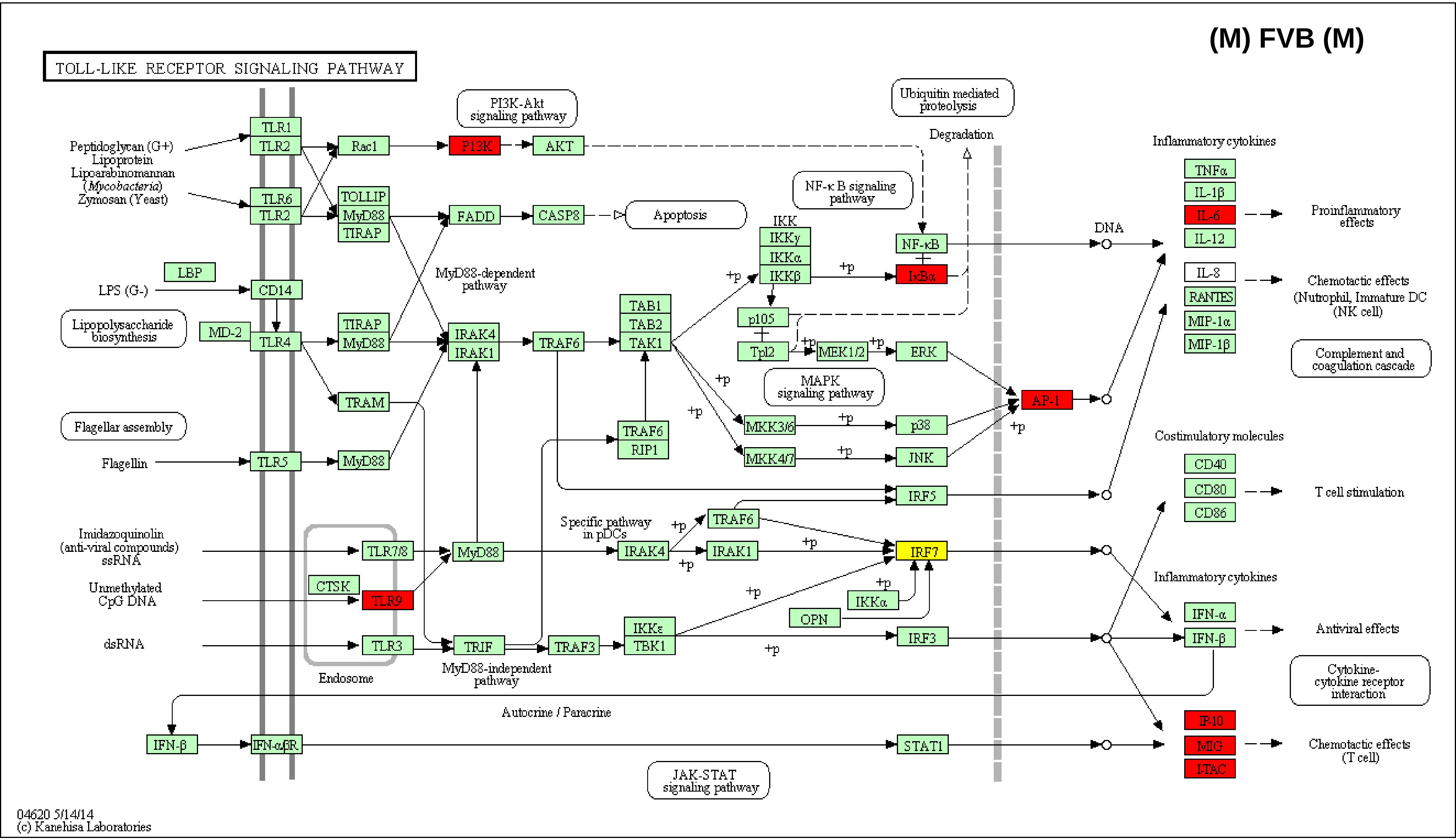

(M) FVB (M)
TOLL-LIKE RECEPTOR SIGNALING PATHWAY
PI3K-Akt signaling pathway
Ubiquitin mediated proteolysis
Degradation
Inflammatory cytokines
Peptidoglycan (G+)
Lipoprotein
Lipoarabinomannan (Mycobacteria)
Zymosan (Yeast)
TLR1
TLR2
TLR6
Rac1
PI3K
AKT
TOLLIP
MyD88
TIRAP
FADD
CASP8
Apoptosis
NF-κB signaling pathway
IKK
IKKγ
IKKα
IKKβ
NF-κB
IκBα
+p
DNA
TNFα
IL-1β
IL-6
IL-12
IL-8
RANTES
MIP-1α
MIP-1β
Proinflammatory effects
Chemotactic effects (Nutrophil, Immature DC (NK cell)
Complement and coagulation cascade
LBP
LPS (G-)
CD14
Lipopolysaccharide biosynthesis
MD-2
TLR4
MyD88-dependent pathway
IRAK4
IRAK1
TRAF6
TAB1
TAB2
TAK1
p105
Tpl2
MEK1/2
ERK
MAPK signaling pathway
AP-1
TRAM
MKK3/6
p38
MKK4/7
JNK
RIP1
Flagellar assembly
Flagellin
TLR5
Costimulatory molecules
CD40
CD80
CD86
T cell stimulation
IRF5
Imidazoquinolin (anti-viral compounds) ssRNA
TLR7/8
Specific pathway in pDCs
IRF7
CTSK
TLR9
Unmethylated CpG DNA
IKKα
OPN
dsRNA
TLR3
TRIF
TRAF3
IKKε
TBK1
IRF3
Endosome
MyD88-independent pathway
IFN-α
IFN-β
Antiviral effects
Cytokine-cytokine receptor interaction
Autocrine / Paracrine
IFN-β
IFN-α/βR
STAT1
IP-10
MIG
I-TAC
Chemotactic effects (T cell)
JAK-STAT signaling pathway
04620 5/14/14
(c) Kanehisa Laboratories

(N) FVB (F)

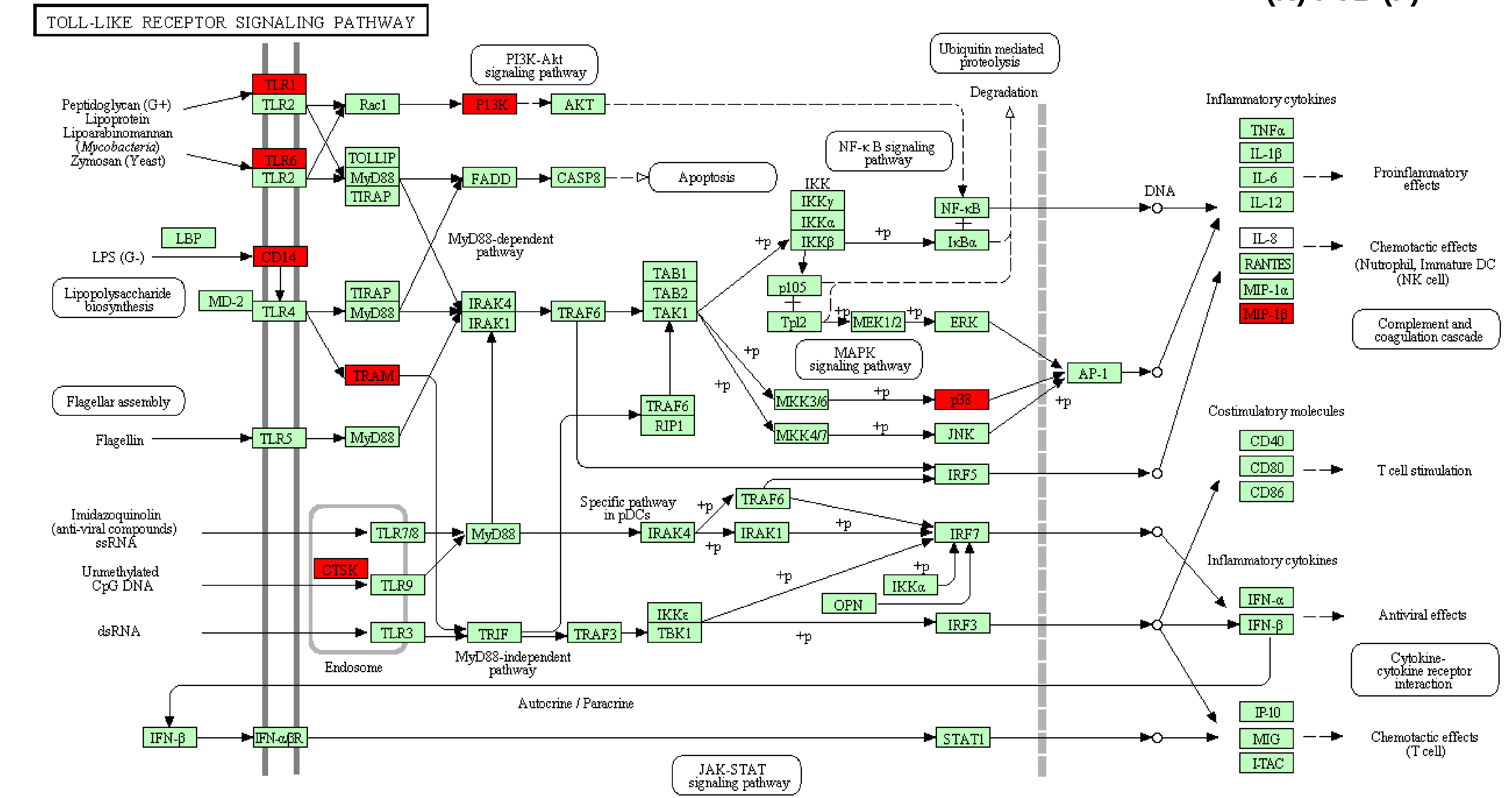

04620 5/14/14
(c) Kanehisa Laboratories

(O) 129/SvJ (M)

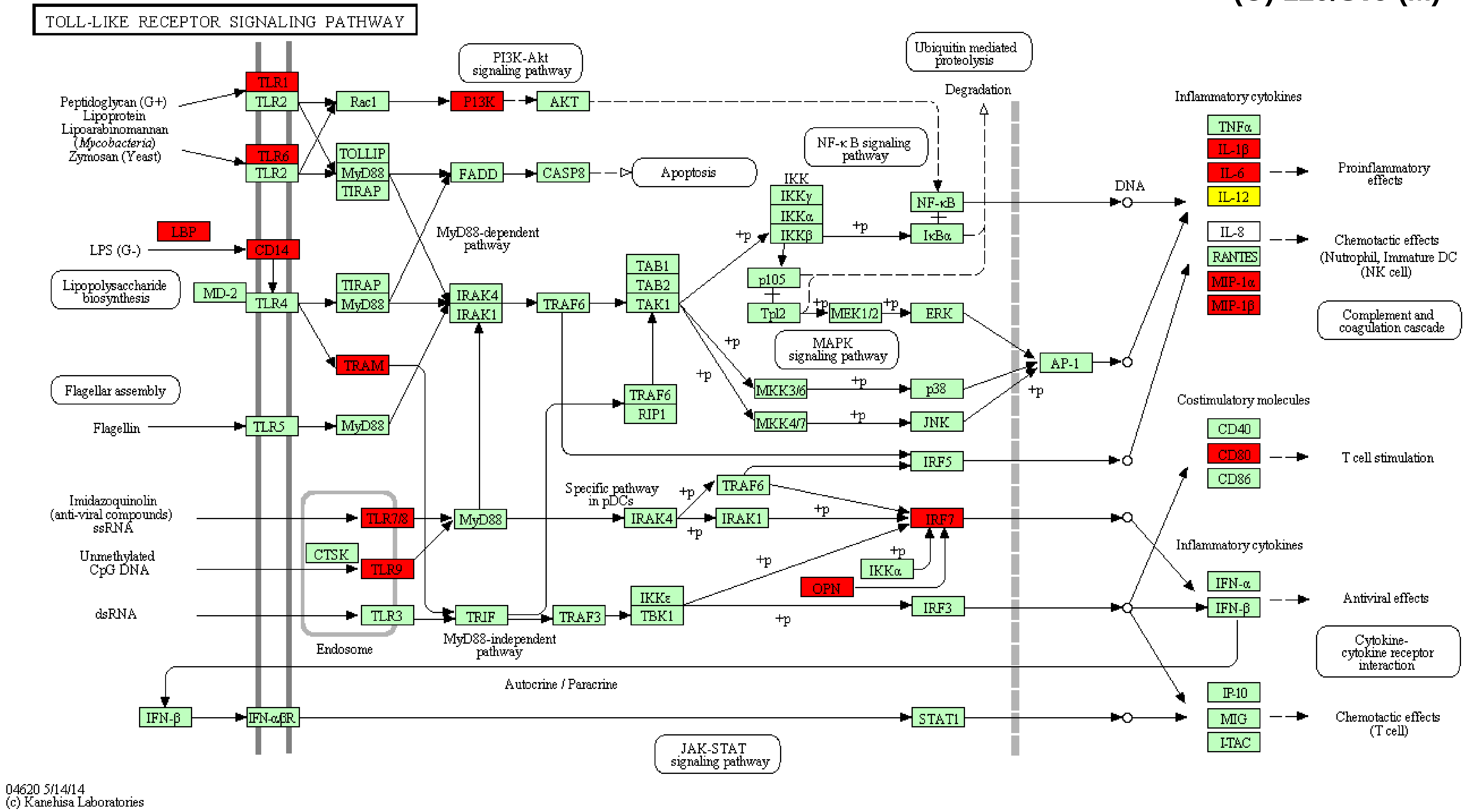

TOLL-LIKE RECEPTOR SIGNALING PATHWAY
Peptidoglycan (G+)
Lipoprotein
Lipoarabinomannan
(Mycobacteria)
Zymosan (Yeast)
TLR1
TLR2
TLR6
TLR2
Rac1
PI3K-Akt signaling pathway
PI3K
AKT
TOLLIP
MyD88
TIRAP
FADD
CASP8
Apoptosis
LBP
LPS (G-)
CD14
Lipopolysaccharide biosynthesis
MD-2
TLR4
TIRAP
MyD88
MyD88-dependent pathway
IRAK4
IRAK1
TRAF6
TAB1
TAB2
TAK1
TRAM
Flagellar assembly
Flagellin
TLR5
MyD88
TRAF6
RIP1
Ubiquitin mediated proteolysis
Degradation
NF-κB signaling pathway
IKK
IKKγ
IKKα
IKKβ
NF-κB
IκBα
p105
Tpl2
MEK1/2
ERK
MAPK signaling pathway
MKK3/6
p38
MKK4/7
JNK
AP-1
DNA
IRF5
+p
Inflammatory cytokines
TNFα
IL-1β
IL-6
IL-12
Proinflammatory effects
IL-8
RANTES
MIP-1α
MIP-1β
Chemotactic effects
(Nutrophil, Immature DC
(NK cell)
Complement and coagulation cascade
Costimulatory molecules
CD40
CD80
CD86
T cell stimulation
Imidazoquinolin
(anti-viral compounds)
ssRNA
Unmethylated CpG DNA
dsRNA
TLR7/8
CTSK
TLR9
TLR3
Endosome
MyD88
Specific pathway in pDCs
IRAK4
TRAF6
IRAK1
IRF7
IKKα
OPN
TRIF
MyD88-independent pathway
TRAF3
IKKε
TBK1
IRF3
Inflammatory cytokines
IFN-α
IFN-β
Antiviral effects
Cytokine-cytokine receptor interaction
Autocrine / Paracrine
IFN-β
IFN-α/βR
STAT1
JAK-STAT signaling pathway
IP-10
MIG
I-TAC
Chemotactic effects
(T cell)
04620 5/14/14
(c) Kanehisa Laboratories

(P) 129/SvJ (F)

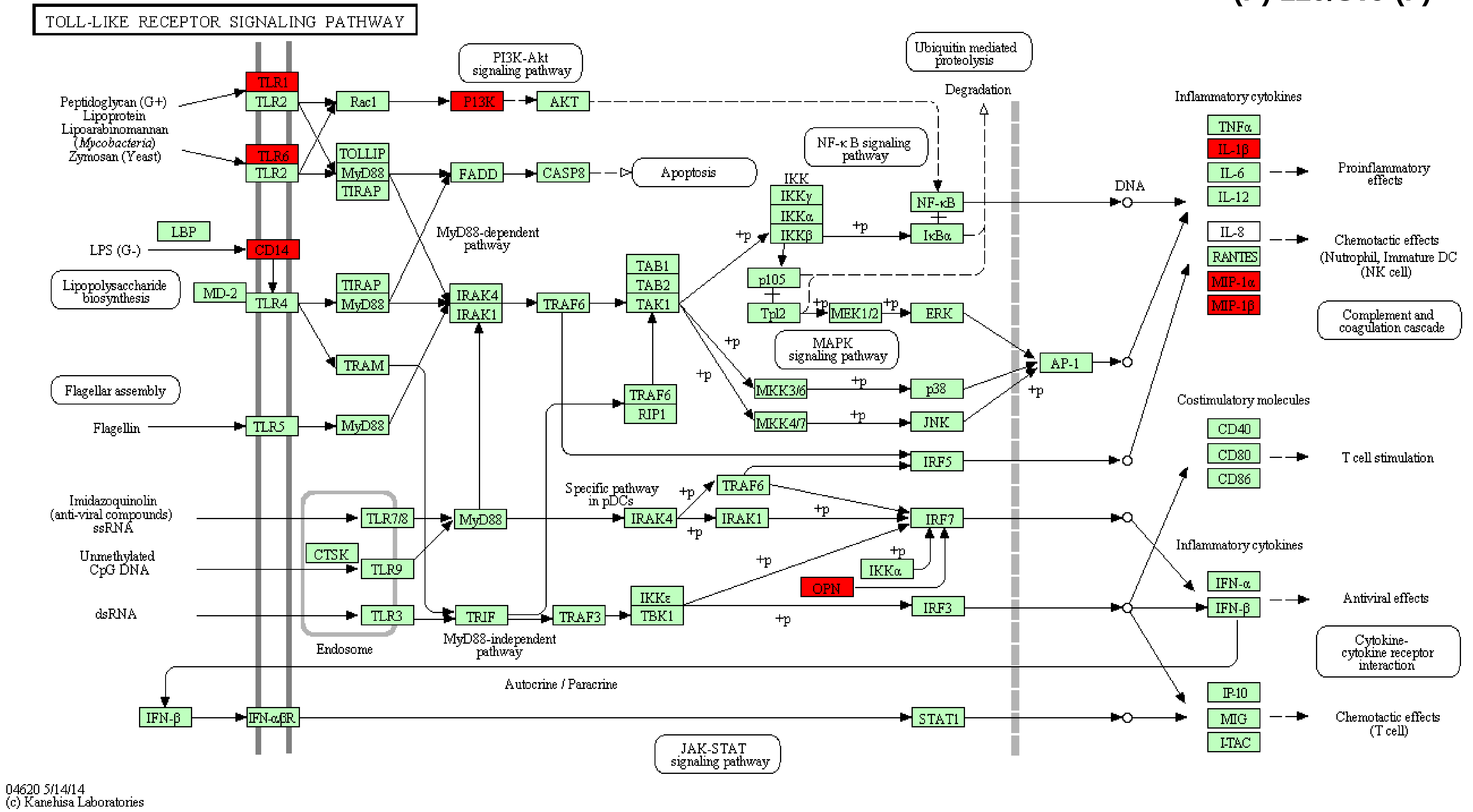
TOLL-LIKE RECEPTOR SIGNALING PATHWAY
Peptidoglycan (G+)
Lipoprotein
Lipoarabinomannan
(Mycobacteria)
Zymosan (Yeast)
TLR1
TLR2
TLR6
TLR2
Rac1
PI3K
AKT
PI3K-Akt signaling pathway
Ubiquitin mediated proteolysis
Degradation
TOLLIP
MyD88
TIRAP
FADD
CASP8
Apoptosis
NF-κB signaling pathway
IKK
IKKγ
IKKα
IKKβ
NF-κB
IκBα
DNA
LBP
LPS (G-)
CD14
MD-2
TLR4
Lipopolysaccharide biosynthesis
TIRAP
MyD88
MyD88-dependent pathway
IRAK4
IRAK1
TRAF6
TAB1
TAB2
TAK1
p105
Tpl2
MEK1/2
ERK
MAPK signaling pathway
TRAM
Flagellar assembly
Flagellin
TLR5
MyD88
TRAF6
RIP1
MKK3/6
p38
MKK4/7
JNK
AP-1
IRF5
+p
Imidazoquinolin
(anti-viral compounds)
ssRNA
TLR7/8
MyD88
Specific pathway in pDCs
IRAK4
IRAK1
TRAF6
IRF7
Unmethylated CpG DNA
CTSK
TLR9
IKKα
OPN
dsRNA
TLR3
Endosome
TRIF
MyD88-independent pathway
TRAF3
IKKε
TBK1
IRF3
Autocrine / Paracrine
IFN-β
IFN-α/βR
STAT1
JAK-STAT signaling pathway
Inflammatory cytokines
TNFα
IL-1β
IL-6
IL-12
Proinflammatory effects
IL-8
RANTES
MIP-1α
MIP-1β
Chemotactic effects
(Nutrophil, Immature DC
(NK cell)
Complement and coagulation cascade
Costimulatory molecules
CD40
CD80
CD86
T cell stimulation
Inflammatory cytokines
IFN-α
IFN-β
Antiviral effects
Cytokine-cytokine receptor interaction
IP-10
MIG
I-TAC
Chemotactic effects
(T cell)
04620 5/14/14
(c) Kanehisa Laboratories

(Q) MOLF (M)

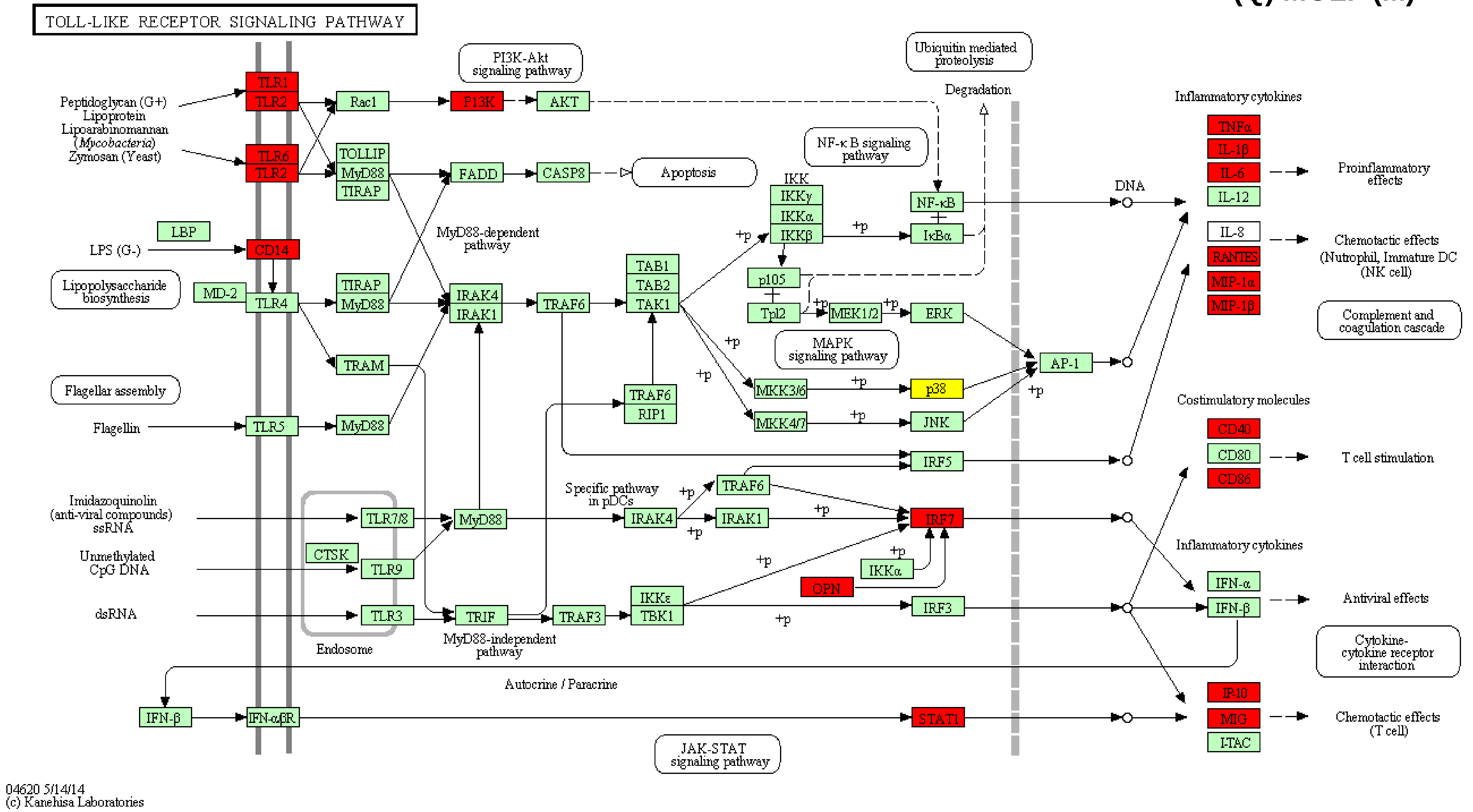
TOLL-LIKE RECEPTOR SIGNALING PATHWAY
PI3K-Akt signaling pathway
Ubiquitin mediated proteolysis
Degradation
Inflammatory cytokines
Peptidoglycan (G+)
Lipoprotein
Lipoarabinomannan
(Mycobacteria)
Zymosan (Yeast)
TLR1
TLR2
TLR6
TLR2
Rac1
PI3K
AKT
TOLLIP
MyD88
TIRAP
FADD
CASP8
Apoptosis
NF-κB signaling pathway
IKK
IKKγ
IKKα
IKKβ
NF-κB
IκBα
DNA
TNFα
IL-1β
IL-6
IL-12
Proinflammatory effects
IL-8
RANTES
MIP-1α
MIP-1β
Chemotactic effects
(Nutrophil, Immature DC
(NK cell)
Complement and coagulation cascade
LBP
LPS (G-)
CD14
MyD88-dependent pathway
Lipopolysaccharide biosynthesis
MD-2
TLR4
TIRAP
MyD88
IRAK4
IRAK1
TRAF6
TAB1
TAB2
TAK1
p105
Tpl2
MEK1/2
ERK
MAPK signaling pathway
AP-1
TRAM
Flagellar assembly
TRAF6
RIP1
MKK3/6
p38
MKK4/7
JNK
+p
Costimulatory molecules
CD40
CD80
CD86
T cell stimulation
Flagellin
TLR5
MyD88
IRF5
Imidazoquinolin
(anti-viral compounds)
ssRNA
TLR7/8
MyD88
Specific pathway in pDCs
IRAK4
TRAF6
IRAK1
IRF7
Inflammatory cytokines
Unmethylated CpG DNA
CTSK
TLR9
IKKα
OPN
IFN-α
IFN-β
Antiviral effects
dsRNA
TLR3
Endosome
TRIF
MyD88-independent pathway
TRAF3
IKKε
TBK1
IRF3
Cytokine-cytokine receptor interaction
Autocrine / Paracrine
IFN-β
IFN-α/βR
STAT1
IP-10
MIG
I-TAC
Chemotactic effects
(T cell)
JAK-STAT signaling pathway
04620 5/14/14
(c) Kanehisa Laboratories

(R) MOLF (F)

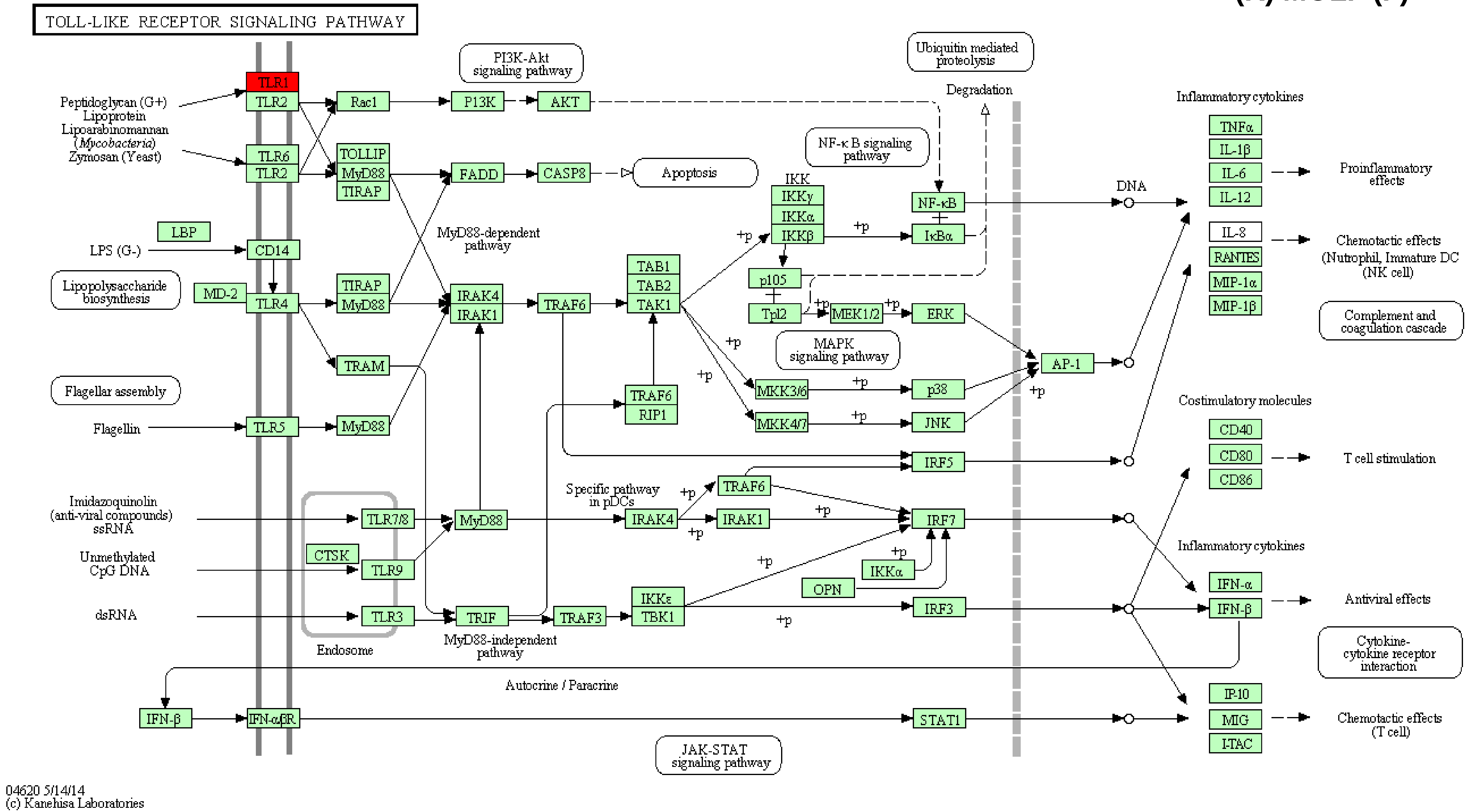

TOLL-LIKE RECEPTOR SIGNALING PATHWAY
TLR1
TLR2
Peptidoglycan (G+)
Lipoprotein
Lipoarabinomannan
(Mycobacteria)
Zymosan (Yeast)
TLR6
TLR2
Rac1
PI3K
AKT
PI3K-Akt signaling pathway
TOLLIP
MyD88
TIRAP
FADD
CASP8
Apoptosis
LBP
LPS (G-)
CD14
MD-2
TLR4
Lipopolysaccharide biosynthesis
TIRAP
MyD88
IRAK4
IRAK1
TRAF6
TAB1
TAB2
TAK1
MyD88-dependent pathway
TRAM
Flagellar assembly
Flagellin
TLR5
MyD88
TRAF6
RIP1
Ubiquitin mediated proteolysis
Degradation
NF-κB signaling pathway
IKK
IKKγ
IKKα
IKKβ
NF-κB
IκBα
p105
Tpl2
MEK1/2
ERK
MAPK signaling pathway
MKK3/6
p38
MKK4/7
JNK
AP-1
IRF5
+p
DNA
Inflammatory cytokines
TNFα
IL-1β
IL-6
IL-12
Proinflammatory effects
IL-8
RANTES
MIP-1α
MIP-1β
Chemotactic effects (Nutrophil, Immature DC (NK cell)
Complement and coagulation cascade
Costimulatory molecules
CD40
CD80
CD86
T cell stimulation
Imidazoquinolin (anti-viral compounds) ssRNA
TLR7/8
MyD88
Specific pathway in pDCs
IRAK4
TRAF6
IRAK1
IRF7
Unmethylated CpG DNA
CTSK
TLR9
IKKα
OPN
dsRNA
TLR3
Endosome
TRIF
TRAF3
IKKε
TBK1
IRF3
MyD88-independent pathway
Inflammatory cytokines
IFN-α
IFN-β
Antiviral effects
Cytokine-cytokine receptor interaction
Autocrine / Paracrine
IFN-β
IFN-α/βR
STAT1
JAK-STAT signaling pathway
IP-10
MIG
I-TAC
Chemotactic effects (T cell)
04620 5/14/14
(c) Kanehisa Laboratories
