# Supplementary material for: Imiquimod has strain-dependent effects in mice and does not uniquely model human psoriasis
Source: Genome Med. 2017 Mar 9;9:24. doi: 10.1186/s13073-017-0415-3 (PMC5345243; doi:10.1186/s13073-017-0415-3)
Supplement: Additional file 13: — Psoriasis-specific genes with altered expression in psoriasis lesions but not other human skin diseases. (PDF 1382 kb) [file 13073_2017_415_MOESM13_ESM.pdf]

**Additional File 13. Psoriasis-specific genes with altered expression in psoriasis lesions but not other human skin diseases.** (A) Psoriasis-increased DEGs with the most psoriasis-specific expression patterns. Psoriasis-specificity is measured by the psoriasis specificity index (horizontal axis), with larger values indicating more psoriasis-specific expression. Heatmap colors depict IMQ expression responses for homologous mouse genes. (B) Match scores. Scores represent average FC (IMQ/CTL) of the 30 genes shown in (A) (right margin: average score for each strain-sex group; red font: $P < 0.05$, Wilcoxon rank sum test). (C) *Atp1b1* expression (B6 and BALB/C). *Atp1b1* is the most psoriasis-specific gene with elevated expression in psoriasis lesions. (D) Psoriasis-decreased DEGs with the most psoriasis-specific expression patterns. Larger psoriasis specificity index values indicate more psoriasis-specific expression. Heatmap colors depict IMQ expression responses for homologous mouse genes. (E) Match scores. Scores represent average FC (CTL/IMQ) of the 30 genes shown in (D). (F) *Krt13* expression (B6 and BALB/C). *Krt13* is the most psoriasis-specific gene with decreased expression in psoriasis lesions. In (C) and (F), groups without the same letter differ significantly ($P < 0.05$, Tukey honest significant difference; Error bars: standard error of the mean).

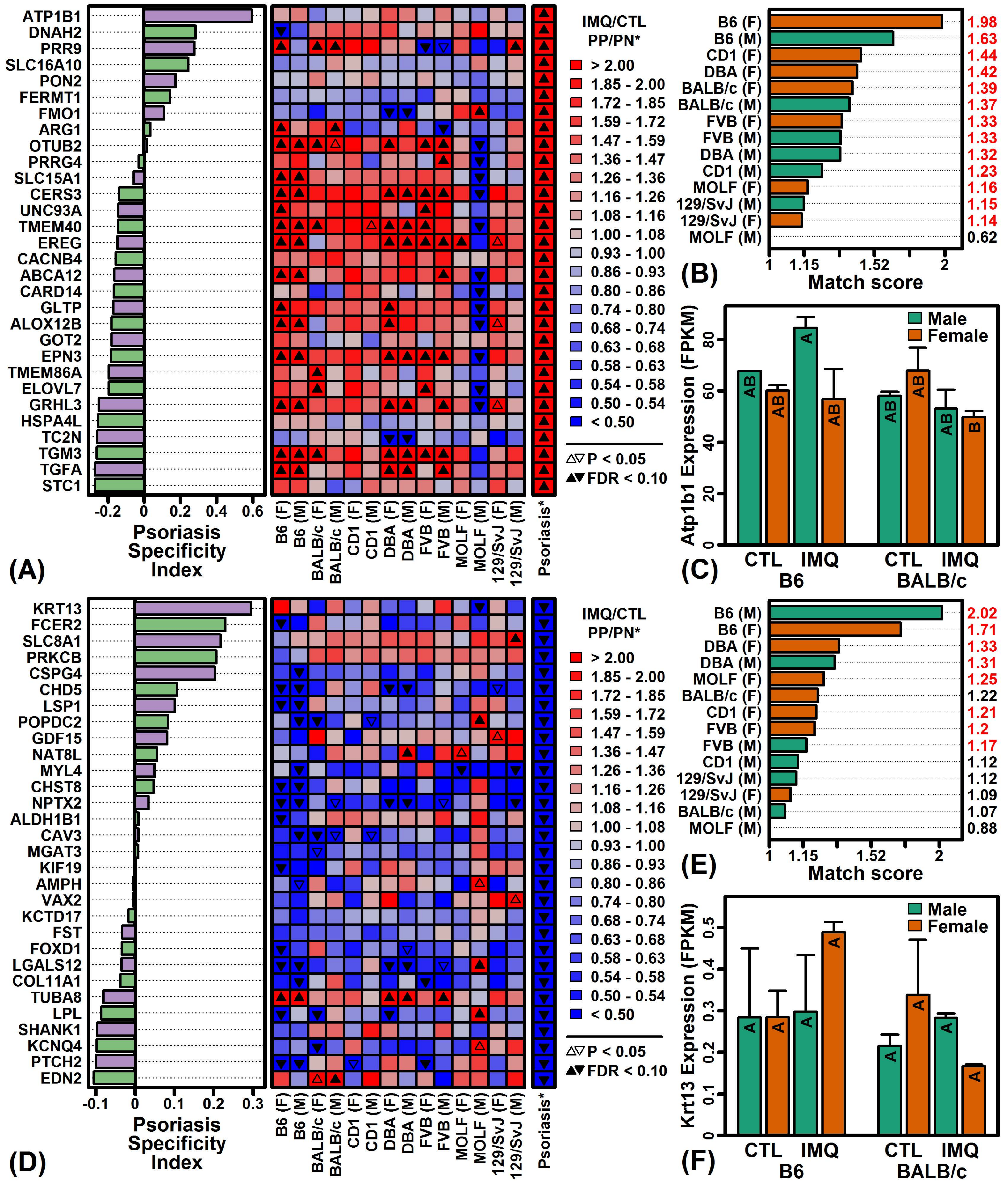
(A)
ATP1B1
DNAH2
PRR9
SLC16A10
PON2
FERMT1
FMO1
ARG1
OTUB2
PRRG4
SLC15A1
CERS3
UNC93A
TMEM40
EREG
CACNB4
ABCA12
CARD14
GLTP
ALOX12B
GOT2
EPN3
TMEM86A
ELOVL7
GRHL3
HSPA4L
TC2N
TGM3
TGFA
STC1
-0.2
0
0.2
0.4
0.6
Psoriasis Specificity Index
B6 (F)
B6 (M)
BALB/c (F)
BALB/c (M)
CD1 (F)
CD1 (M)
DBA (F)
DBA (M)
FVB (F)
FVB (M)
MOLF (F)
MOLF (M)
129/SvJ (F)
129/SvJ (M)
Psoriasis*
IMQ/CTL
PP/PN*
> 2.00
1.85 - 2.00
1.72 - 1.85
1.59 - 1.72
1.47 - 1.59
1.36 - 1.47
1.26 - 1.36
1.16 - 1.26
1.08 - 1.16
1.00 - 1.08
0.93 - 1.00
0.86 - 0.93
0.80 - 0.86
0.74 - 0.80
0.68 - 0.74
0.63 - 0.68
0.58 - 0.63
0.54 - 0.58
0.50 - 0.54
< 0.50
P < 0.05
FDR < 0.10
(B)
B6 (F) 1.98
B6 (M) 1.63
CD1 (F) 1.44
DBA (F) 1.42
BALB/c (F) 1.39
BALB/c (M) 1.37
FVB (F) 1.33
FVB (M) 1.33
DBA (M) 1.32
CD1 (M) 1.23
MOLF (F) 1.16
129/SvJ (M) 1.15
129/SvJ (F) 1.14
MOLF (M) 0.62
1
1.15
1.52
2
Match score
(C)
Atp1b1 Expression (FPKM)
0
20
40
60
80
Male
Female
AB
AB
A
AB
AB
AB
AB
B
CTL
IMQ
B6
CTL
IMQ
BALB/c
(D)
KRT13
FCER2
SLC8A1
PRKCB
CSPG4
CHD5
LSP1
POPDC2
GDF15
NAT8L
MYL4
CHST8
NPTX2
ALDH1B1
CAV3
MGAT3
KIF19
AMPH
VAX2
KCTD17
FST
FOXD1
LGALS12
COL11A1
TUBA8
LPL
SHANK1
KCNQ4
PTCH2
EDN2
-0.1
0
0.1
0.2
0.3
Psoriasis Specificity Index
B6 (F)
B6 (M)
BALB/c (F)
BALB/c (M)
CD1 (F)
CD1 (M)
DBA (F)
DBA (M)
FVB (F)
FVB (M)
MOLF (F)
MOLF (M)
129/SvJ (F)
129/SvJ (M)
Psoriasis*
IMQ/CTL
PP/PN*
> 2.00
1.85 - 2.00
1.72 - 1.85
1.59 - 1.72
1.47 - 1.59
1.36 - 1.47
1.26 - 1.36
1.16 - 1.26
1.08 - 1.16
1.00 - 1.08
0.93 - 1.00
0.86 - 0.93
0.80 - 0.86
0.74 - 0.80
0.68 - 0.74
0.63 - 0.68
0.58 - 0.63
0.54 - 0.58
0.50 - 0.54
< 0.50
P < 0.05
FDR < 0.10
(E)
B6 (M) 2.02
B6 (F) 1.71
DBA (F) 1.33
DBA (M) 1.31
MOLF (F) 1.25
BALB/c (F) 1.22
CD1 (F) 1.21
FVB (F) 1.2
FVB (M) 1.17
CD1 (M) 1.12
129/SvJ (M) 1.12
129/SvJ (F) 1.09
BALB/c (M) 1.07
MOLF (M) 0.88
1
1.15
1.52
2
Match score
(F)
Krt13 Expression (FPKM)
0
0.1
0.2
0.3
0.4
0.5
Male
Female
A
A
A
A
A
A
A
A
CTL
IMQ
B6
CTL
IMQ
BALB/c
